# Analysis of variance and its sources in UAV-based multi-view thermal imaging of wheat plots

**DOI:** 10.1016/j.plaphe.2025.100046

**Published:** 2025-04-30

**Authors:** Simon Treier, Lukas Roth, Andreas Hund, Helge Aasen, Lilia Levy Häner, Nicolas Vuille-dit-Bille, Achim Walter, Juan M. Herrera

**Affiliations:** a Cultivation Techniques and Varieties in Arable Farming Group, Agroscope, Route de Duillier 60, Nyon, 1260, Switzerland; b ETH Zürich, Institute of Agricultural Sciences, Universitätstrasse 2, Zürich, 8092, Switzerland; c Earth Observation of Agroecosystems Team, Agroecology and Environment Division, Agroscope, Reckenholzstrasse 191, Zürich, 8046, Switzerland

**Keywords:** Plant phenotyping, Aerial thermography, Thermal drift, Spatial correction, High throughput field phenotyping, Viewing geometry

## Abstract

Canopy temperature (CT) estimates from drone-based uncooled thermal cameras are prone to confounding effects, which affects the interpretability of CT estimates. Experimental sources of variance, such as genotypes and experimental treatments blend with confounding sources of variance such as thermal drift, spatial field trends, and effects related to viewing geometry. Nevertheless, CT is gaining popularity to characterize crop performance and crop water use, and as a proxy measurement of stomatal conductance and transpiration. Drone-based thermography was therefore proposed to measure CT in agricultural experiments. For a meaningful interpretation of CT, confounding sources of variance must be considered. In this study, the multi-view approach was applied to examine the variance components of CT on 99 flights with a drone-based thermal camera. Flights were conducted on two variety testing field trials of winter wheat over two years with contrasting meteorological conditions in the temperate climate of Switzerland. It was demonstrated how experimental sources of variance can be disentangled from confounding sources of variance and on average more than 96.5 ​% of the initial variance could be explained with experimental and confounding sources combined. Not considering confounding sources led to erroneous conclusions about phenotypic correlations of CT with traits such as yield, plant height, fractional canopy cover, and multispectral indices. Based on extensive and diverse data, this study provides comprehensive insights into the manifold sources of variance in CT measurements, which supports the planning and interpretation of drone-based CT screenings in variety testing, breeding, and research.

## 1 Introduction

Canopy temperature (CT) of wheat (*Triticum aestivum* L.) is a proxy-measurement of stomatal conductance (*e.g.,* [[1], [2], [3], [4]]) and transpiration [5] that is negatively correlated with yield in well-watered conditions [3,[6], [7], [8], [9]], *i.e.* a lower CT is generally associated with higher yield. CT is more sensitive to changes in the water status of plants than other optical measurements such as the Normalized Difference Vegetation Index (NDVI), and shows a faster response time to physiological changes in the plant [[10], [11], [12], [13]]. This makes CT especially interesting for measuring plant performance in dry and/or hot conditions. Therefore, it was proposed to be used in cereal breeding (*e.g.,* [[1], [2], [3], [4], [6], [14], [15], [16]]), in research and precision agriculture (*e.g.,* [[17], [18], [19]]), *e.g.*, to detect water stress. Thermal infrared (TIR) cameras mounted on drones allow the efficient measurement of many experimental units [2,15]. However, various sources of variance can adversely affect TIR measurements and increase uncertainties when estimating CT. Spatiotemporal and geometric patterns superimpose with the effects of specific genotypes or treatments (*e.g.,* [20]). Therefore, the measurement and interpretation of CT data is not trivial [15]. Elaborated measurement procedures and statistical methods are needed to disentangle the sources of variance that influence CT measurements.

The most important sources of variance and their main drivers/causes are summarized in Table 1. First, CT is sensitive to short-term changes in environmental conditions. Solar radiation, air temperature, relative humidity of the air, vapor pressure deficit (VPD), and cloud cover are all interlinked and affect CT measurements directly by changing the heat balance of the canopy, for example, by fluctuating radiation or indirectly by impacting stomatal conductance [3,6,8,15]. Such environmental effects might mask more subtle plant responses [11]. To reduce distortions by the environment, it is recommended to fly in stable conditions, *i.e.* when there are no clouds or haze and wind speeds are low with no gusts. However, also under stable conditions, solar radiation and VPD are constantly changing, and the conditions may differ at the beginning and the end of the flight, particularly for long-duration flights [27].

**Table 1:** Overview on most important sources of variance of drone-based thermal measurements.

Variance source	Variance driver/cause	Temporal behavior	Primary type of correction	Reference
Solar radiation	Weather	Dynamic (short term)	Temporal	Reynolds et al. [4]

VPD^a^				Idso et al. [21]
Wind	Reynolds et al. [4]
Thermal drift	Sensor temperature	Dynamic (short term)	Temporal	Nugent et al. [22]
Non-uniformity effects			–	Nugent et al. [22]
Field heterogeneity	soil water content, water logging, soil compaction etc.	Stable throughout single flights	Spatial	Perich et al. [15]
Treatment effects	Field management	Stable throughout single flights	Treatment	Reynolds et al. [4]
Plant height	Genotype/Field management	Stable throughout single flights	Genotype/Treatment	Prashar et al. [23]
Soil cover				Aasen et al. [24]
Stomatal conductance				Reynolds et al. [4]
Phenology				Prashar et al. [23]
Stay green				Anderegg et al. [1]
Rooting depth (water availability)				Reynolds et al. [4]
Vignetting	Sensor/Optics	Rather stable	Geometric	Aasen et al. [24]
BRDF^b^	Viewing geometry	Stable throughout single flights	Geometric	Schaepman et al. [25]
Apparent soil cover				Pask et al. [8]
Atmospheric effects				Berni et al. [26]

a Vapor pressure deficit (VPD).

b Bidirectional reflectance distribution function (BRDF).

Due to a limited payload of drones, uncooled thermal cameras are commonly used in field phenotyping. They often depend on Vanadium Oxide (VOx) microbolometers, which are arranged in focal plane arrays (FPA, *e.g.,* [[12], [15], [20], [28], [29], [30]]). Such cameras are prone to thermal drift, where the measured temperature varies as a result of short-term temperature fluctuations the FPA of the sensor and the camera optics are exposed to [12,22]. This holds true for both radiometrically calibrated and uncalibrated cameras. Thermal drift is known to be a significant confounding source of variance in CT measurements, and the literature proposes different approaches to correct for it in data pre-processing (*e.g.,* [[20], [27], [30], [31]] and analysis [29]. Kelly et al. [20] and Yuan et al. [30] examined the importance of wind conditions on the sensor as an important driver of sensor temperature and TIR readings. Kelly et al. [20], Malbéteau et al. [32] and Treier et al. [29] demonstrated how TIR readings change with relative motion along the main flight direction of the drone as a result of changing wind conditions the sensor is exposed to.

Thermal drift is not homogeneous throughout the FPA and leads to non-uniformity effects (*e.g.,* [22]). Other non-uniformity effects are caused by dark signal noise and vignetting [24]. The latter describes the alteration of the signal in dependence of the path of radiation through the lens optics, leading to distortions where the edges of the image appear darker (or cooler for thermography) than the central regions [20,24,30].

The viewing geometry also alters the TIR readings. The signal is subject to surface anisotropy, that is, the signal is altered depending on the direction from which it is emitted/reflected from the surface [15,24,33], which can be described with a bidirectional reflectance distribution function (BRDF) [25,34]. Additionally, viewing geometry alters the fraction of soil visible between rows in row crops. At a more nadir-oriented view, the fractional canopy cover (FCC) is at a minimum and increases with more oblique viewing geometry, mainly perpendicular to the sowing rows [35]. It is therefore recommended to measure at oblique angles [3,6,8]. However, with drone-based cameras, this is not always possible, and excluding nadir-oriented measurements comes with trade-offs. Just including measurements from oblique angles is more canopy specific and less related to FCC, but it also decreases the maximum number of measurements that can be taken per plot when less oblique measurements are excluded, which is deteriorating the consistency of the measurements [29].

While the sources of variance of the TIR measurements considered so far included instantaneous environmental conditions, the sensor, and the viewing geometry, the experiment at observation itself constitutes an important source of variance. In the case of wheat variety testing trials, different genotypes are arranged in the field in blocks of multiple randomly arranged replications which allow to disentangle effect of field heterogeneity from genotype effects. Field heterogeneity might be caused by differences in soil water content, soil depth, soil fertility, water logging, soil compaction, root disease, and other factors (*e.g.,* [[2], [3], [36]]). For some studies, different field management practices, *e.g.*, different irrigation or fertilization regimens, are applied to the genotypes. Genotypes, treatments, and field heterogeneity lead to distinct phenotypes, and phenotype-specific CT differences might be explained by different traits and not stomatal conductance alone, although phenotypic traits often are interlinked with each other. Quantitative trait loci have been shown to be often pleiotropic or co-located for CT and yield, above-ground biomass, plant height, and other traits (*e.g.,* [3], citet in [6],[37]). CT is strongly affected by above-ground biomass, morphological parameters such as plant height, FCC, leaf area index (LAI), rooting behavior, late senescence behavior, and consequently a larger green area during later stages, and even by the spatial orientation of leafs and spikes [1,3,4,15,23,38,39]. All of these sources are not independent. FCC for example might be caused by the genotype but also the field management or field heterogeneity, and an increased FCC might reduce the impact of the viewing geometry as also at nadir view, little soil is visible when FCC is saturated.

To observe the effects of genotypes and treatments on CT, uncertainties of CT estimates must be mitigated by estimating and correcting confounding sources of variance. For example Rebetzke et al. [3] applied a mixed model and included the time of CT sampling as a fixed linear effect. Treier et al. [29] proposed a multi-view approach in which CT estimates were derived from sequences of thermal images. Unlike approaches where CT estimates rely on orthomosaics, multi-view allowed for multiple CT estimates per plot and flight and to estimate covariates related to trigger timing and viewing geometry for each single measurement. The authors showed how the inclusion of trigger timing as a random effect in linear mixed models was allowing to increase consistency and genotype-specificity of the CT estimates. The aim of the study at hand was to empirically demonstrate how the multi-view approach can be used to disentangle multiple sources of variance and to separate undesired sources of variance from desired sources in a first step. A second aim was to show why these corrections matter with respect to the interpretability of the data. To that end, the multi-view method was applied in two wheat variety testing trials with contrasting field management regimens in two consecutive years of contrasting meteorological conditions. Complementary measurements were conducted to test hypotheses on wind conditions on the sensor, canopy cover, LAI, above-ground biomass, and plant height as important drivers of the thermal signal.

## 2 Methods

### 2.1 Field experiments and data acquisition

TIR measurements were conducted in two winter wheat variety testing experiments for two consecutive years (2020–2021 and 2021–2022) in the fields of the Agroscope agricultural research station, Changins, Switzerland [46°23′55.4″N 6°14′20.4″E, 425 ​m. a.s.l., the World Geodetic System (WGS) 84]. The soil of the experimental site is a shallow Calcaric Cambisol [40,41].

One trial comprised 30 modern registered European winter wheat varieties and is further referred to as the EuVar trial. The same varieties were seeded for the two years in three different treatment regimes. In the “maximal” regimen, one growth regulator and one fungicide treatment were applied. In the “medium” regimen, there was only the growth regulator application and not the fungicide application. In the “minimal” regimen, neither a growth regulator nor a fungicide was applied (see Tables S1 and S2 for more details). Fertilizers and herbicides were applied in three splits and at equal rates to all treatments according to the Proof of Ecological Performance (PEP) certification guidelines [42], which represent a minimal standard for best-practice for conventional agriculture in Switzerland. Each variety-treatment combination was repeated on three plots. Within single plots, eight sowing rows of the same wheat genotype were sown with a spacing of 15 ​cm between them, resulting in an observable canopy of about 1.25 ​m ​× ​6.7 ​m each. Within blocks of 3 by 10 plots, the genotypes were randomly distributed, and these blocks were randomly nested within three treatment replicates. Each replicate contained three blocks, and each block was treated with one of the three treatments. The 270 plots of the experiment span over 27 rows (which followed the tractor track direction) and 10 columns (Fig. S1). This experiment, the TIR data acquisition and multi-view processing were first described in Treier et al. [29], where the same authors demonstrated the robustness of the multi-view approach and the method was shown to outperform commonly used orthomosaic-based approaches. The Methods are partially described here and in the Supplementary Materials for clarity, but for more information, it is referred to the study mentioned.

The second trial, further denoted SwiVar, comprised modern winter wheat genotypes and mixtures of two genotypes. The genotypes included registered varieties and candidate lines for inscription in the Swiss list of recommended wheat varieties. In the first year, there were 34 pure genotypes and two genotype mixtures. In the second year, there were 35 pure genotypes and one mixture. 31 genotypes and one mixture stayed the same between the two years. This performance trial included two different nitrogen treatment regimens. In one regimen, nitrogen fertilization was carried out according to common local agricultural practice following the PEP guidelines. In the second fertilizer regimen, no nitrogen fertilizer was applied. Herbicides were applied in both treatments according to the PEP guidelines. Each genotype was repeated in each treatment three times, resulting in 216 plots with the same row spacing as in EuVar and a canopy of about 1.25 ​m ​× ​4.3 ​m each. Within the treatments, the plots were arranged in a randomized complete block design and the treatments were grouped into two separate blocks of 6 x 18 plots due to restrictions in available space and for simplifying nitrogen management (Fig. S2). In 2021, a sowing error occurred in three plots of one replication of SwiVar, which were seeded with the variety of the border plots and for these three genotypes, there were just two replications in the fertilized regimen (Fig. S2). The three plots were included in the analysis as genotype “border”. SwiVar22 received an irrigation of 30 ​mm on 2022-05-23 due to lack of rain (Fig. S5).

The different experiment-year combinations are further referred to as EuVar21, EuVar22, SwiVar21 and SwiVar22 according to year of harvest. Tables S1 and S2 give an overview on the different treatments, fertilizer applications and the most important field interventions while Table S3 displays details on the chemical products used.

Air temperature, rainfall, radiation, wind speed, wind direction, relative humidity and VPD were obtained by a weather station of Meteoswiss which was located about 800 ​m from the experimental site at Changins [46°24′3.7″N 6°13′39.6″E, 458 ​m. a.s.l., WGS 84].

2021 was a relatively cool year with almost 700 ​mm precipitation between the beginning of the year and harvest, while there were just 280 ​mm of precipitation for the same period in 2022. The temperature was on average 2.9 ​°C warmer from May to harvest for 2022 compared to 2021, and wheat developed faster in 2022 with the heading occurring 6 days earlier (Fig. S5). Harvest was 20 days earlier for EuVar22 compared to EuVar21. SwiVar22 was harvested 13 days before SwiVar21.

Flights were carried out between the onset of flowering and mid-senescence. In 2021, flights were conducted on two dates in each trial. In 2022, flights were conducted on four dates on EuVar22 and on six dates on SwiVar22 respectively. On specific dates, multiple flights were conducted at different time slots. To account for short-term variability, within each time slot at least two, mostly three flights were conducted with the same settings. A group of flights that were conducted at one time slot and date is further called a flight campaign. In total, 39 flights were performed on EuVar and 60 on SwiVar (for more details, see Supplementary Materials sections S4 & S5). Drone flights generally took place under close to optimal conditions with relatively low wind, although conditions in 2022 were more optimal than in 2021, when high and semitransparent cloud layers led to fluctuating light intensities for some flights in 2021 (Fig. S6 and S7).

The drone flew over the plots at a height of approximately 40 ​m, which allowed for a ground sampling distance (GSD) of about 5.2 cm/pixel. With a plot width of 1.5 ​m, this GSD resulted in more than 20 rows of pixels within the plots after excluding the border areas of the plots, while still allowing for relatively short flights. The heading of drone and TIR camera was set to remain stable throughout the flight and did not change with changes in flight path direction. The resulting flight duration was between 6 and 9 ​min depending on the wind conditions and the total area recorded. The settings used resulted in an image pattern in which each spot in the trial was recorded on at least nine images from different perspectives. The camera was pointing toward the ground orthogonally (*i.e.* in nadir orientation). An uncalibrated DJI Zenmuse XT TIR sensor (SZ DJI Technology Co. Ltd., China) was used and a detailed description of the equipment and the settings used and of flight planning can be found in Supplementary Materials section S6. The experiments were neighbored by border plots and other experiments. To increase the number of measurements available for trend estimation, the flights covered not just the experiments but all wheat plots in the respective field surroundings, that is, border plots and other experiments on the same field. This helped reduce border effects by improving temporal and spatial corrections, as described in Treier et al. [29]. Supplementary Materials section S10 summarizes the pre-flight procedure. In short, the camera was turned on at least 15 ​min before each flight to allow the temperature signal to stabilize. The TIR images were saved as radiometric JPEG format. Following the protocol of Treier et al. [29], no radiometric calibration was applied for later processing and only the internal calibration provided by the manufacturer was used.

For post-processing in the Structure-from-Motion-based photogrammetry software Agisoft Metashape (Agisoft LCC, St. Peterburg, Russia) and to allow time series analysis, thermal ground control points (GCPs) were distributed in the field in an evenly spaced shifted grid pattern (for more details, see Supplementary Materials section S11).

For the multi-view approach, digital elevation models (DEM) were needed on which the images could be projected. DEMs were based on both, TIR images and RGB images. For more details on the creation of DEMs, refer to Supplementary Materials section S7.

### 2.2 TIR image pre-processing

From radiometric JPEG format, 14-bit TIFF files were derived, representing temperature in °*C* x 1000 by using a Python 3.8 script [43], a modified version of the Flir Image Extractor (https://github.com/ITVRoC/FlirImageExtractor).

The 14-bit TIFF files of the radiometric images as well as the RGB images were aligned in the structure-from-motion-based software Agisoft Metashape Professional (Agisoft LLC, St. Petersburg, Russia) and georeferenced (for details, see Supplementary Materials section S12). Plot masks were created for each plot in Qgis 3.16 [44], to determine the regions of interest (ROIs) from which the data was used for analysis. A buffer of at least 25 ​cm was applied on plot width and length to account for inaccuracies in georeferencing.

The image information was reduced to a single value for each plot in each image by using the optimal percentile of all pixel values within each plot in each image. The procedure for finding an optimal percentile was described in Treier et al. [29]. In short, for each percentile, heritabilities were calculated from a mixed model with the R package SpATS [45]. The resulting percentile-heritability relations were plotted for graphical comparison and optimal percentile selection. The same percentile was used for the aggregation of all flights on one experiment within one year (for more details, see Supplementary Materials section S8).

### 2.3 Multi-view pre-processing

The single images were projected on the RGB DEMs by ray tracing as described in Roth et al. [35], Roth et al. [46] and Treier et al. [29]. This allowed the projection of geographic coordinates (*e.g.* EPSG:2056 reference system) to image coordinates. As a result, plot masks of ROIs were created for each trigger position (*i.e.* for each image), where at least one plot was entirely inside the field of view (FOV) of the camera. For each plot on each TIFF file, all percentiles were extracted with a Python 3.8 script.

**Fig. 1:**
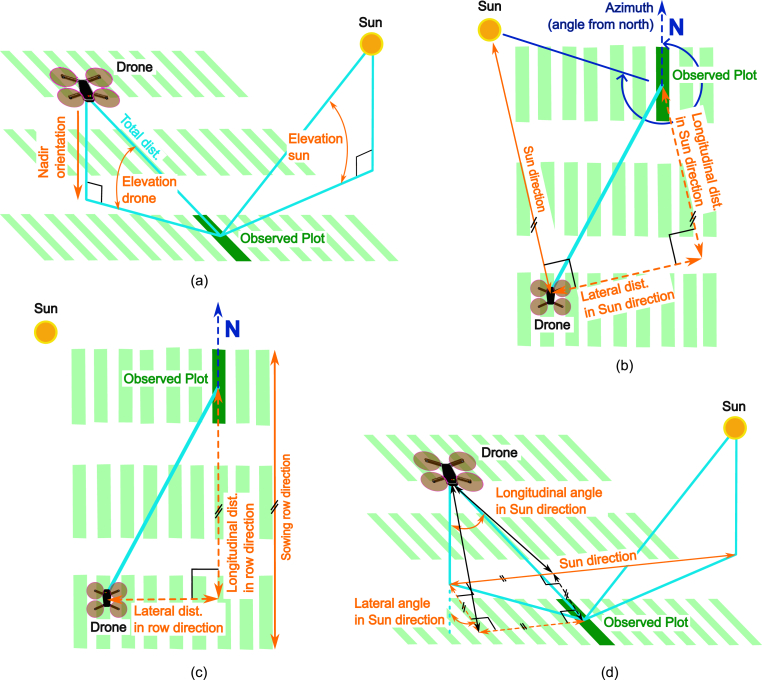
By knowing the position of the sun, the position of the plot and the position and orientation of the camera when an image is triggered (a), different geometric relations can be calculated. The position directly below the drone is in nadir orientation. The vertical angle at which drone and sun are seen from the observed plot are the elevations of drone and sun respectively. The azimuth of the sun is the clockwise horizontal angle at which the sun is seen from the observed plot from north (b). The position of the plot can be described as planar distance between drone and plot in direction of the sun (b) or in sowing row direction (c). Another option to describe the positon of the plot relative to the drone is by viewing angles as is shown for angles relative to sun direction (d), but not shown for the sowing row direction. Elements in the principal optical planes in drone or sun direction are in bright blue, cardinal direction in dark blue. The dimensions of interest and related covariates are in orange. Small black angle marks and short parallel black lines indicate perpendicularity and parallelism respectively.

As plot-wise data was extracted for each image, the trigger timing could be determined from image meta data. The trigger timing of each image and the position of the experiment was known while the position of the sun was determined for each measurement as azimuth and elevation angle in Python using a script by John Clark Craig (https://levelup.gitconnected.com/python-sun-position-for-solar-energy-and-research-7a4ead801777, 2021). As Cartesian (*i.e.* orthogonal) coordinates were used and the position of the sun, the position of the plot centers and the position and orientation of the camera at the moment when the image was triggered were known, this allowed to calculate the geometric relations between sun, plot and drone by trigonometry as listed in Table S5 and illustrated in Fig. 1 (for more details, see Supplementary Materials section S9).

### 2.4 TIR data post-processing

**Fig. 2:**
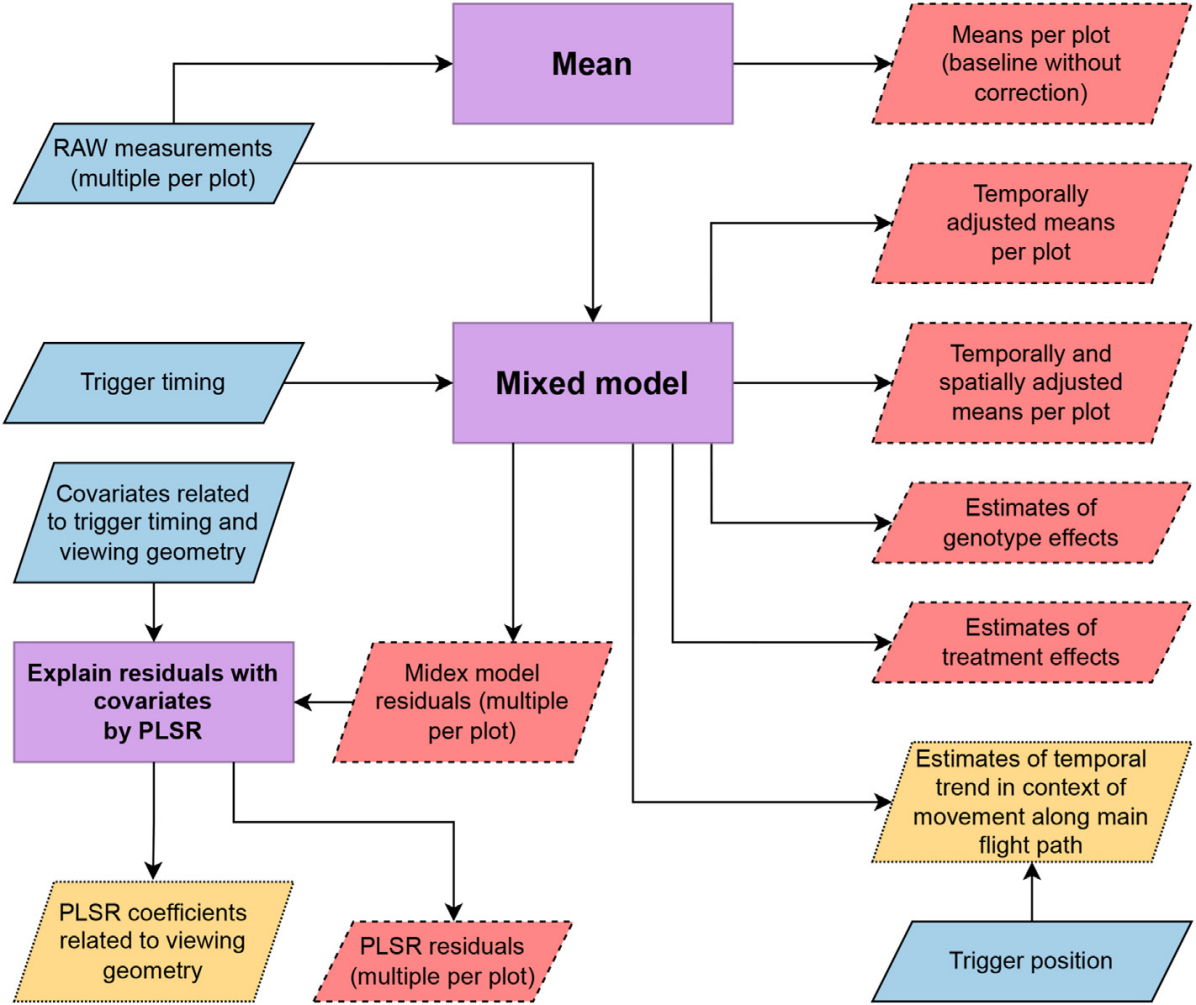
Flow-chart depicting the process of step-wise TIR measurement correction. TIR measurements and covariates (blue/solid-border parallelograms) were processed in different steps (purple rectangles) to derive estimates of plot-wise canopy temperature and residuals (red/dashed-border parallelograms) as well as trends related to trigger timing and viewing geometry (yellow/dotted-border parallelogram).

After data extraction, the contribution of the different sources of CT variance to the total CT variance was estimated and CT was corrected for confounding sources of variance. Although the sources of variance might differ, they might be corrected by the same type of correction (Table 1). For example, while variance sources related to weather are ideally avoided by flying without wind and clouds, they still might affect the measurements in a temporal pattern. Such temporal variation mixes with the thermal drift, and is thus corrected by the same type of correction [27]. Correction for the different types of correction was achieved in a two-step approach (Fig. 2), as the computational burden of a one-stage approach was too heavy for multi-view data [29], and stage-wise approaches are proposed for the analysis of complex agricultural trials [47]. In a first stage, the TIR measurements were corrected for non-geometric sources of variance. The residuals of the first stage were then analyzed to reveal the importance of geometric effects in a partial least squares regression (PLSR) analysis in a second stage. A plot-wise mean was calculated as a reference baseline. In the following, the two-stage approach is described in detail.

#### 2.4.1 Mixed model

The multi-view method provided several CT estimates for each plot (originating from different images). For each measurement, covariates related to trigger timing and viewing geometry were available which were used to analyze sources of variance and to correct the TIR measurements.

A mixed model (Eq. (1)) was fitted in ASReml-R [48] to correct for temporal and spatial trends and experimental design factors (experiments, genotypes, treatments, replications). ASReml-R was chosen over other mixed model software due to its capability to model complex variance structures, which was important for the best possible consideration of nested structures (*e.g.* border plots) and temporal trends in this study. This mixed model used was introduced and tested for robustness in Treier et al. [29], where the single terms are explained in detail and mentioned here for clarity. Plot-based repeated CT measurements *θ*_ijknp_ for the *i*th genotype, *j*th trigger event, *k*th treatment, *n*th replication, and *p*th plot were decomposed in factors related to genotypes (*θ*_i_), treatments (*τ*_k_), replications (*r*_n_) and plots (*ϕ*_*p*_) within a field. A temporal trend was modeled as a smooth spline *f*_spl_(*λj*) along the sequential trigger events *λ*_*j*_, where a trigger event *j* corresponds to a specific thermal image. A spatial model comprised two one-dimensional autocorrelation parts in row direction *f*_AR(1)_(*r*(*p*)) (following tractor tracks) and column direction *f*_AR(1)_(*c*(*p*)), where *f*_AR(1)_ is a first order autoregression function of respective rows and columns at positions of plots in row direction *r*(*p*) and column direction *c*(*p*). In addition, a two-dimensional spatial autocorrelation *f*_AR(1)×AR(1)_(*c*(*p*), *r*(*p*)) was included in the spatial model. *e*_ijknp_ are measurement specific residuals. Genotypes and treatments were given unique IDs for each experiment covered in one flight, so the same genotype or treatment ID did not appear in the experiment of interest (*i.e.*, either EuVar or SwiVar) and also surrounding experiments or border plots at the same time, reducing the complexity of data structure to be handled by the models. The *k* experiment-specific treatments therefore also implicitly describe the different experiments. An interaction between the *i*th genotype and the *k*th treatment (*θτ*)_*ik*_ was applied only to the experiments of interest. For parts of other surrounding experiments and border plots, a simple additive effect was assumed for genotype and treatment for simplicity and to reduce computational capacity needed. ASReml-R allows to specify model terms for subsets of data with the “at()” statement, and the data could be processed differently for plots belonging to different experiments and border plots with the same ASReml-R model. The interaction between the *k*th treatment and the *n*th replication (*τr*)_*kn*_ was just applied to EuVar, as the treatments were nested within the replications in EuVar, but not in SwiVar.

(1) $$\begin{array}{lll} \theta_{\mathrm{ijknp}} & =\left(\theta_{i}+\tau_{k}+\phi_{p}+r_{n}+{\left(\theta \tau \right)}_{ik}+{\left(\tau r\right)}_{kn}+\right) & \left(\left(\text{Design}-\text{Factors}\right)\right)\\ & \left(f_{\text{AR}\left(1\right)\times \text{AR}\left(1\right)}\left(c\left(p\right),r\left(p\right)\right)+f_{\text{AR}\left(1\right)}\left(c\left(p\right)\right)+f_{\text{AR}\left(1\right)}\left(r\left(p\right)\right)+\right) & \left(\left(\text{Spatial}-\text{Autoregression}\right)\right)\\ & \left(f_{\text{spl}}\left(\lambda_{j}\right)+\right) & \left(\left(\text{Temporal}-\text{Trend}\right)\right)\\ & \left(e_{\mathrm{ijknp}}\right) & \left(\left(\text{Residuals}\right)\right) \end{array}$$

#### 2.4.2 Estimate the temporal trend

Mixed models decompose variance into variance components and the different components can subsequently be included in models to predict the effects of individual variables. The temporal trend was estimated as the effect of the *j*th trigger event/image along the duration of a single flight, modeled with a smooth spline *f*_spl_(*λ*_*j*_) in Eq. (1).

#### 2.4.3 Plot-wise CT estimates

After fitting the models by Eq. (1), single plot-wise CT values $\left({\hat{\theta }}_{p}\right)$ were estimated with different prediction models to estimate the effect and importance of different variables within the mixed model.

To have a baseline for comparison, the mean plot temperature ${\hat{\theta }}_{p}^{\mathrm{mean}}$ was calculated on the measurements of the individual images *j* available for one plot *p* without applying the mixed model or considering any covariates,

(2) $${\hat{\theta }}_{p}^{\mathrm{mean}}=\mathrm{mean}\left(\theta_{jp}\right).$$

A first mixed model-based prediction model included all variance components of the mixed models except for the temporal trend *λj* (Eq. (3)). It estimated the individual plot-wise CT values as the sum of genotype effects (*θ*_*i*_), treatment effects (*τ*_*k*_), plot effects (*ϕ*_*p*_), row *r*_*p*_, column *c*_*p*_ and replication effects (*r*_*n*_) at the position of plot p,

(3) $${\hat{\theta }}_{p}^{t\text{\_}c}={\hat{\theta }}_{ikpr_{\left(p\right)}c_{\left(p\right)}n}=\left(\theta_{i}+\tau_{k}+\phi_{p}+r_{p}+c_{p}+r_{n}\right).$$

By discarding the temporal trend in the prediction, the plot-wise estimates were plot-wise means ${\hat{\theta }}_{p}^{t\text{\_}c}$ adjusted along the temporal dimension and therefore temporally corrected (*t*_*c*). In the next step, the spatial trends of row *r*_*p*_ and column *c*_*p*_ were discarded in prediction,

(4) $${\hat{\theta }}_{p}^{ts\text{\_}c}={\hat{\theta }}_{\mathrm{ikpn}}=\left(\theta_{i}+\tau_{k}+\phi_{p}+r_{n}\right).$$

The plot-wise estimates ${\hat{\theta }}_{p}^{ts\text{\_}c}$ of Eq. (4) were temporally and spatially corrected (*ts*_*c*). To consider possibly strong treatment effects, for each flight, the mean treatment temperatures were calculated and subtracted from ${\hat{\theta }}_{p}^{ts\text{\_}c}$,

(5) $${\hat{\theta }}_{p}^{t\text{\_}defl}={\hat{\theta }}_{\mathrm{ipn}}=\left({\hat{\theta }}_{\mathrm{ikpn}}-mean\left(\tau_{k}\right)\right).$$

The plot-wise estimates ${\hat{\theta }}_{p}^{t\text{\_}defl}$ represent the sum of a genotpye, a genotype-treatment interaction, a plot, and a replication effect after subtracting a mean treatment effect *mean*(*τ*_*k*_), leaving out all other effects of Eq. (1). They are temporally and spatially corrected, and treatment effects were deflated (*t*_*defl*), meaning that only a possible genotype-treatment interaction is left in the estimate, but not the main treatment effect.

Predictive models (Eq. (3), Eq. (4) & Eq. (5)) just comprised plots belonging to EuVar or SwiVar. As uncooled and uncalibrated TIR cameras provide a low absolute temperature accuracy, just relative temperature differences between the plots were analyzed from this stage onward [20,49].

For a comparison of the effects of the single variables, the variance of the plot-wise estimates derived from the different prediction methods (Eq. (2) - Eq. (5)) was calculated for all flights.

#### 2.4.4 Multispectral measurements

The trials were also monitored with an airborne Micasense RedEdge-MX Dual multispectral camera (MicaSense Inc., Seattle, Washington, USA) throughout the growing season. With multispectral data, vegetation indices (VI) were calculated to obtain approximative estimates of LAI and biomass. The images were aligned in Agisoft to generate 10 band orthophotos covering all the experiments. Details on the spectral properties of the 10 bands of the sensor are described in Table S6. Based on these bands, four VIs were calculated. DVI, SAVI and EVI (see Table 2 for full names and equations) are commonly used VIs to estimate the LAI of wheat [50] while SAVI was also shown to be correlated with above-ground biomass [51]. NDVI was calculated as a reference to the emissivity [52] of the plants. The same masks as for the TIR images were used to mark ROIs on the multispectral orthomosaics. The 50th percentile (median) was used to aggregate VI values within single ROIs to single values with a Python 3.8 [43] script for subsequent analysis (for more details, see Supplementary Materials section S15).

**Table 2:** Multispectral VIs used to approximate biomass (DVI, SAVI, EVI) and LAI (SAVI) and as a reference to the emissivity (NDVI).

Index	Full name	Formula	Reference
DVI	Difference Vegetation Index	$DVI=NIR_{842}-Red_{668}$ (6)	Tucker [53]
EVI	Enhanced Vegetation Index	$EVI=2.5\cdot \frac{NIR_{842}-Red_{650}}{NIR_{842}+6\cdot Red_{650}-7.5\cdot Blue_{444}+1}$ (7)	Huete et al. [54]
NDVI	Normalized Difference Vegetation Index	$NDVI=\frac{NIR_{842}-Red_{668}}{NIR_{842}+Red_{668}}$ (8)	Rouse et al. [55]
SAVI	Soil Adjusted Vegetation Index	$SAVI=1.5\cdot \frac{NIR_{842}-Red_{650}}{NIR_{842}+Red_{650}+0.5}$ (9)	Huete [56]

VIs were recorded on multiple dates and the VIs were correlated to CT that was measured at the date closest to the VI recording.

#### 2.4.5 Estimate the spatial trend

The spatial trend of the plots *p* across the field in row *c*_(*p*)_ and column *c*_(*p*)_ direction ${\hat{\theta }}_{r_{\left(p\right)}c_{\left(p\right)}}$ was estimated as the difference between the plot-wise CT estimates after a temporal correction ${\hat{\theta }}_{p}^{t\text{\_}c}$ and after a temporal and spatial correction ${\hat{\theta }}_{p}^{ts\text{\_}c}$,

(10) $${\hat{\theta }}_{r_{\left(p\right)}c_{\left(p\right)}}={\hat{\theta }}_{p}^{t\text{\_}c}-{\hat{\theta }}_{p}^{ts\text{\_}c}=\left({\hat{\theta }}_{ikpr_{\left(p\right)}c_{\left(p\right)}n}-{\hat{\theta }}_{\mathrm{ikpn}}\right).$$

Assuming the consistency of spatial effects between flights, these plot-wise spatial trends would be correlated between flights. As a larger number of observations per plot is assumed to increase the repeatability of the estimations [29], spatial trends were calculated for all flights individually, but also for all flights within a campaign simultaneously. With at least two flights per campaign, this was increasing the number of observations per plot at least two-fold.

### 2.5 Geometric effects

As shown in Table 1, multiple sources of variance have a geometric effect on CT readings. They can be caused by vignetting, viewing geometry-related effects, atmospheric effects, and geometric emission and reflectance patterns (*i.e.* BRDF).

Two different methods were applied to account for geometric effects. In a first approach, the covariance of the residuals *e*_*ijknp*_ of the mixed models (Eq. (1)) with geometric covariates was examined by PLSR with the R-package PLS [57].

The linear relations between geometric covariates (Table S5) and residuals were visually identified in an exploratory data analysis and where necessary, trigonometric transformations were applied to angular covariates for linearization. Covariates with an apparent linear relationship to the residuals (Table 3) were included in the PLSR model. In addition, the interaction between the longitudinal distance in the direction of the sun and the sine of the elevation angle of the drone was part of the PLSR analysis, as it describes the path of light from the sun to the drone. The inclusion of the two terms without interaction does not describe the path adequately as positions in front and behind the drone in the direction of the sun get the same values.

**Table 3:** Covariates with evident trends were identified among all orignial covariates and transformations were applied to linearize the trends. Several trends can describe the same spatial dimension (*e.g.* Lateral in direction of sowing rows).

Linearized covariates	Dimension	Transformation	Name in Model
Sine of the elevation angle of the drone	Elevation of the drone	Sine	Drone-Elevation-sin

Lateral distance in direction of sowing rows	Lateral in direction of sowing rows	None	RowDir-lat-Dist
Absolute lateral distance in direction of sowing rows		Absolute value	RowDir-lat-Dist-abs
Cosine of lateral angle in direction of sowing rows		Cosine	RowDir-lat-Angl-cos
Absolute value of lateral angle in direction of sowing rows		Absolute value	RowDir-lat-Angl-abs
Longitudinal distance in direction of sowing rows	Longitudinal in direction of sowing rows	None	RowDir-lon-Dist
Absolute longitudinal distance in direction of sowing rows		Absolute value	RowDir-lon-Dist-abs
Cosine of longitudinal angle in direction of sowing rows		Cosine	RowDir-lon-Angl-cos
Absolute value of longitudinal angle in direction of sowing rows		Absolute value	RowDir-lon-Angl-abs
Lateral distance in direction of the sun	Lateral in direction of the sun	None	SunDir-lat-Dist
Absolute lateral distance in direction of the sun		Absolute value	SunDir-lat-Dist-abs
Cosine of lateral angle in direction of the sun		Cosine	SunDir-lat-Angl-cos
Absolute value of lateral angle in direction of the sun		Absolute value	SunDir-lat-Angl-abs
Longitudinal distance in direction of the sun	Longitudinal in direction of the sun	None	SunDir-lon-Dist
Absolute longitudinal distance in direction of the sun		Absolute value	SunDir-lon-Dist-abs
Cosine of longitudinal angle in direction of the sun		Cosine	SunDir-lon-Angl-cos
Absolute value of longitudinal angle in direction of the sun		Absolute value	SunDir-lon-Angl-abs
Interaction between longitudinal distance in direction of the Sun and sine of the elevation angle of the drone	Interaction SunDir-Drone-Elevation	None	Interact.-SunDir-Drone

Trigger timing	Time	None	Trigger-time

Total distance between drone and plot	Distance	None	Dist-tot

The PLSR coefficients were calculated for each covariate and each flight to determine which covariate explained the most of the variance of the residuals *e*_ijknp_ from the pre-processing model (Eq. (1)). Several linearized covariates in the PLSR model described the same spatial dimension (Table 3). With the aims of avoiding redundancy and simplifying the model, the model was reduced to contain only relevant dimensions. Relative PLSR coefficient magnitudes *β*_rel,i_ were calculated within each flight and each covariate as:

(11) $$\beta_{\mathrm{rel,i}}=\frac{\left|\beta_{i}\right|}{\sum_{i=1}^{n}|\beta_{i}|},$$

where *β*_i_ denotes the PLSR coefficient of the *i*th of *n* covariates. To determine the least descriptive covariates, the medians of relative magnitude of the covariates *β*_i_ over all flights j were calculated.

(12) $$\beta_{\mathrm{med,i}}=med\left\{\left|\beta_{\mathrm{rel,i;j}}\right|\right\}$$

Covariates with the lowest median were skipped in a supervised backward feature elimination until the most descriptive transformation types and dimensions were left in the model (similar to methods summarized in [58]).

In a second approach to account for geometric effects, a generalized ex ante vignetting correction was applied as described in Treier et al. [29]. A generalized vignetting correction image was created in an indoor experiment, with its pixel values representing a mean vignetting effect as relative temperature difference within an image under controlled conditions. The pixel values of the correction image were then subtracted from the corrsponding pixels of all TIR images (for more details, see Supplementary Materials S16).

Subsequent analysis with mixed models and PLSR analysis was performed on TIR images with and without vignetting correction.

### 2.6 Reference measurements and complementary experiments to better understand phenotypic variability, viewing geometry and thermal drift as sources of CT variance

The mixed model allowed estimation of the contribution of genotypes, experimental treatment regimens, spatial trends, and thermal drift to the overall variance. With the PLSR models, the contribution of viewing geometry to the overall variance was examined. To demonstrate the relationship between CT and the phenotypic variability of genotypes and treatment regimens, reference measurements were made on wheat phenotypes similar to Das et al. [28]. CT was compared with grain yield, FCC, plant height, flag leaf rolling, flag leaf senescence, and multispectral indices as approximations of LAI and above-ground biomass (Table 2) by means of Pearson correlation. Complementary experiments were conducted to demonstrate the impact of apparent soil cover and wind on TIR readings qualitatively.

#### 2.6.1 In-field reference measurements of phenotypic traits

Grain yield was measured with a combine harvester. The water content of the grain was determined with a Dickey-John GAC 2100 grain moisture tester within 24 ​h after harvest and the grain yield per ha was noralized at 15 ​% gravimetric water content.

Plant height was measured with a measuring rod in five randomly chosen spots within each plot, and the mean taken as plot-wise plant height. It was measured from the soil to the tip of the ears without considering awns.

With dry conditions, leaf rolling was observed in season 2022 and visually rated in the field according to Pask et al. [8]. Leaf rolling ratings ranged from 0 to 3 where 0 corresponded to no rolling, 1 to a loosely rolled leaf (< 33 ​% of leaf rolled), 2 to a moderately rolled leaf (34–66 ​% rolled) and 3 to a tightly rolled leaf (> 67 ​% rolled). Flag leaf rolling was compared with the CT measurement performed on a date closest to the rolling scoring date, and the CT differences between the groups were examined with a Wilcoxon signed-rank test.

On the second flight date of EuVar21, senescence had already progressed. Therefore, flag leaf senescence ratings are presented for both EuVar21 measurements dates but not for the other trials. Flag leaf senescence was rated according to Chapman et al. [59] and the ratings correspond to the proportions of senescent yellow leaf area of the flag leaf. 0 ​% corresponds to a fully green leaf and 100 ​% corresponds to a fully senescent leaf.

#### 2.6.2 Qualitative demonstration of impact of apparent soil cover

A handheld calibrated high-resolution thermal camera (VarioCAM High Definition, Jenoptik, Jena, Germany) was used to demonstrate the influence of apparent soil cover qualitatively. This camera also included an RGB sensor which allowed a comparison of visible color images with thermal images of the very same scene.

#### 2.6.3 Multi-view analysis of FCC from RGB data to demonstrate the correlation with CT

To examine the relationship between apparent CT and apparent canopy cover, the FCC was estimated based on RGB images as proposed by Deery et al. [6]. On June 6, 2022, a flight with a DJI Air 2S drone (SZ DJI Technology Co. Ltd., China) was performed in both experiments. The flight height was 20 ​m and the speed was limited to 3 ​m ​s^−1^. The front overlap was 65 ​% and the side overlap was 85 ​%. These settings resulted in a GSD of ≈5.5 ​mm. While such a GSD may be considered too large for a very detailed examination of apparent soil cover, it is sufficient to demonstrate general trends.

Images were saved in 8-bit JPEG format and 16-bit DNG raw format. The DNG files were transformed to TIFF file format in Python 3.8 [43]. Using the interactive image analysis tool Ilastik [60], pixels of the TIFF images were segmented into three classes: green plant, senescent plant, and background. With these classes, FCC could be calculated as:

(13) $$FCC=\frac{PN_{\text{green ​plant}}+PN_{\text{senescent ​plant}}}{PN_{\text{green ​plant}}+PN_{\text{senescent ​plant}}+PN_{\text{background}}},$$

where PN denotes the number of pixels of a specific class in an area of interest. Multiple plot-wise FCC values were fitted with the same mixed model in ASReml-R as CT (Eq. (1)) but replacing CT by FCC. Adjusted means for plot-wise FCC were estimated, and the FCC residuals were analyzed with respect to viewing geometry.

#### 2.6.4 Geometric patterns of atmospheric effects

TIR readings are also affected by atmospheric effects which depend on the path length between the sensor and the target [26,61]. To demonstrate the geometric nature of this effect, a simple data simulation was performed. Assuming a perfect nadir orientation of the sensor, the point directly below the drone is closer to the drone than points toward the edges of the image, *i.e.* the path length between sensor and plot is increased, which increases attenuation of TIR radiation and decreases transmittance of the atmosphere. Taking a simplified assumption of an attenuation of 0.001 ​K ​m^−1^ through the atmosphere [61], we calculated the theoretical attenuation effect at two flight heights (40 ​m and 300 ​m).

#### 2.6.5 Fan experiment to determine the influence of wind

Kelly et al. [20] and et al. [30] described a strong relation between temporal drift of TIR measurements and wind on the sensor. To confirm this link for our sensor, we set up a fan experiment inspired by these two studies. The sensor was placed indoors in a dim environment at room temperature, pointing at a uniform hard foam PVC sheet. A fan and a lamp were used to cool and heat the sensor respectively. The apparent temperature of the PVC sheet and the standard deviation of the pixel-wise temperature were analyzed. To examine whether sudden and strong temperature gradients have a sustained influence on subsequent TIR readings, warm and hot disturbance objects (hands at body temperature and a water cooker with boiling water) were introduced into the scene several times for several seconds each (for more details, see Supplementary Materials S17).

### 2.7 Treatment deflation for correlation estimates

Strong treatment effects can be dominant and mask genotype effects, especially when values are compared by correlations, and the main driver of correlation is a treatment effect. To avoid inflated correlations of possibly dominant treatment effects, correlations were calculated on original data, on data after temporal and spatial correction, and on data after a treatment effect correction. The treatment effects were corrected for by subtracting the mean treatment effects from the plot-wise values after temporal and spatial correction.

### 2.8 Correction of reference measurement and correlation with CT

In-field reference measurements (yield, plant height, FCC, multispectral indices) were fitted with mixed models as done with CT. A model similar to Eq. (1), but without a temporal component was fitted in ASReml-R to correct for spatial trends. CT values before spatial correction (${\hat{\theta }}_{p}^{\mathrm{mean}}$ & ${\hat{\theta }}_{p}^{t\text{\_}c}$) were correlated with uncorrected reference measurements. CT after spatial and temporal correction ${\hat{\theta }}_{p}^{ts\text{\_}c}$ was correlated with spatially corrected reference measurements and treatment deflated CT ${\hat{\theta }}_{p}^{t\text{\_}defl}$ was correlated with treatment deflated reference measurements.

## 3 Results

### 3.1 Percentile choice to aggregate pixel values into uncorrected data

For EuVar21, EuVar22 and SwiVar21, the 50th percentile (median) was chosen to aggregate all pixel values within a ROI into a single value. For EuVar22, the biomass in the non-fertilized part of the experiment was low, leading to large proportions of visible soil in the thermal images. Therefore, the 25th percentile was chosen as it better represented CT, containing fewer background signal from the soil (Fig. S8). The resulting uncorrected plot-wise CT estimates ${\hat{\theta }}_{p}^{\mathrm{mean}}$ (Fig. S9 & Fig. S15) contained strong temporal and spatial trends.

### 3.2 Correcting for temporal and spatial trends

**Fig. 3:**
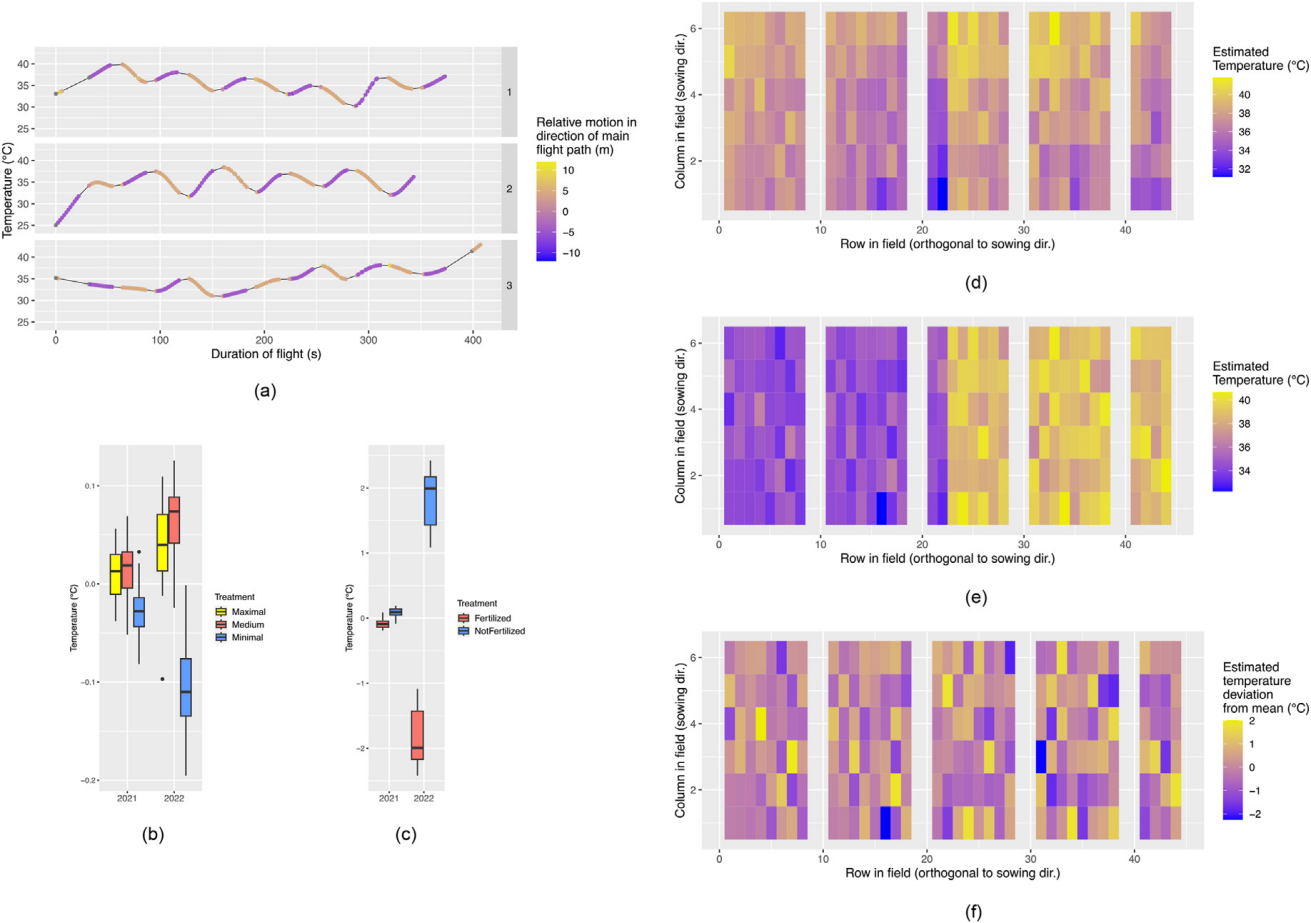
Sources of CT variance not related to viewing-geometry: Thermal drift of TIR measurements for the three flights of the campaign on 2022-06-14 ​at 13:00 was contextualized with the motion in the direction of the main flight path (a). The three rows are the three individual flights within the campaign. The colors indicate the motion in the direction of the main flight path. Purple indicates flights in one direction, and yellow indicates flights in the opposite direction of the flight path grid. For gray points, thermal drift was estimated on the basis of the mixed model, while there was no corresponding measurement of motion along the main flight path. For the estimation of the trends, all three flights were included in the same mixed model (Eq. (1)). The box plots indicate the mean treatment effects for all flights in both years for EuVar (b) and SwiVar (c). After correcting the first flight of the campaign shown in (a) for temporal trends, the adjusted estimates ${\hat{\theta }}_{p}^{t\text{\_}c}$ (Eq. (3)) still contain significant, apparently spatial trends (d). After correction, CT estimates for temporal and spatial trends (Eq. (4)), plot estimates ${\hat{\theta }}_{p}^{ts\text{\_}c}$ contained the genotype, treatment, and plot effects (e). When also subtracting mean treatment temperatures (${\hat{\theta }}_{p}^{t\_defl}$, Eq. (5)), just genotype- and plot-effects were left and the interaction effect between treatment and genotype (f).

The mixed model (Eq. (1)) allowed the estimation of the impact of sources of variance not related to viewing geometry. Fig. 3a shows an example of the temporal trends *f*_spl_(*λ*_*j*_) estimated for the three flights of the SwiVar22 campaign flown on 2022-06-14 ​at 13:00. All three flights of the campaign were processed with the mixed model at once. The color of the line indicates the motion of the drone in the direction of the main flight path. The pattern of increasing and decreasing temperature seemed to be switching with the direction of motion of the drone, but this trend did not seem to be persistent, as it can be seen especially with the third flight, where the patterns of temperature and flight direction did not coincide anymore. Temporal trend estimates for all flights can be looked up at Fig. S10 and Fig. S16 for EuVar and SwiVar respectively. The resulting estimates after removing temporal trends ${\hat{\theta }}_{p}^{t\text{\_}c}$ (Eq. (3)), still contain strong spatial patterns that are not consistent within campaigns (*e.g.* Fig. 3d for the first flight of the same campaign as in Fig. 3a, Fig. S11 and Fig. S17 for all estimates ${\hat{\theta }}_{p}^{t\text{\_}c}$ of EuVar and SwiVar, respectively).

### 3.3 Estimating the effect of experimental treatments

After correcting for temporal and spatial trends (Eq. (4)), plot estimates ${\hat{\theta }}_{p}^{ts\text{\_}c}$ containing genotype, treatment, and plot effects could be derived. When looking at ${\hat{\theta }}_{p}^{ts\text{\_}c}$ for the same flight as Fig. 3d, a strong treatment effect was evident between the left and right sides of the experiment, where the cooler left side corresponded to the fertilized part of the experiment and the hotter right part to the unfertilized part (see Fig. S12 and Fig. S18 for all estimates ${\hat{\theta }}_{p}^{ts\text{\_}c}$ of EuVar and SwiVar, respectively).

Mean treatment effects were estimated for all flights of EuVar (Fig. 3b, Fig. S14) and SwiVar (Fig. 3c, Fig. S20) as deviation from the mean experiment temperature. Within both experiments, the treatment effects were consistent for the two years, but stronger in 2022. However, for EuVar, the treatment effects were small, with a maximum difference of ∼0.15 ​°C in 2021 and ∼0.32 ​°C in 2022. The “minimal” regimen featured the lowest temperature, followed by the “maximal” and “medium” regimen. The differences between the cooler fertilized and the warmer non-fertilized treatment regimen of SwiVar were larger. In 2021, the maximum difference was around ∼0.38 ​°C while for 2022, strong treatment effects were observed with a maximum difference of approximately ∼4.8 ​°C.

### 3.4 Estimating the effect of genotypes and genotype-treatment interactions

When also removing mean treatment effects (Eq. (5)), estimates were corrected for spatial, temporal, and main treatment effects. On an experiment scale, estimates ${\hat{\theta }}_{p}^{t\text{\_}defl}$ did not contain strong spatial trends or treatment effects anymore and appeared relatively flat. The variance between the plot-wise estimates ${\hat{\theta }}_{p}^{t\text{\_}defl}$ as seen in Fig. 3f corresponded to genotypic effects and genotype-treatment interactions without the main treatment effects (see Fig. S11 S13 and Fig. S17 S19 for all estimates ${\hat{\theta }}_{p}^{t\text{\_}defl}$ of EuVar and SwiVar respectively).

### 3.5 Impact of correction for non-geometric trends on variance of estimates

Confounding sources of variance, mainly temporal and spatial trends, contributed significantly more to total variance than experimental sources of variance related to the phenotypes.

**Fig. 4:**
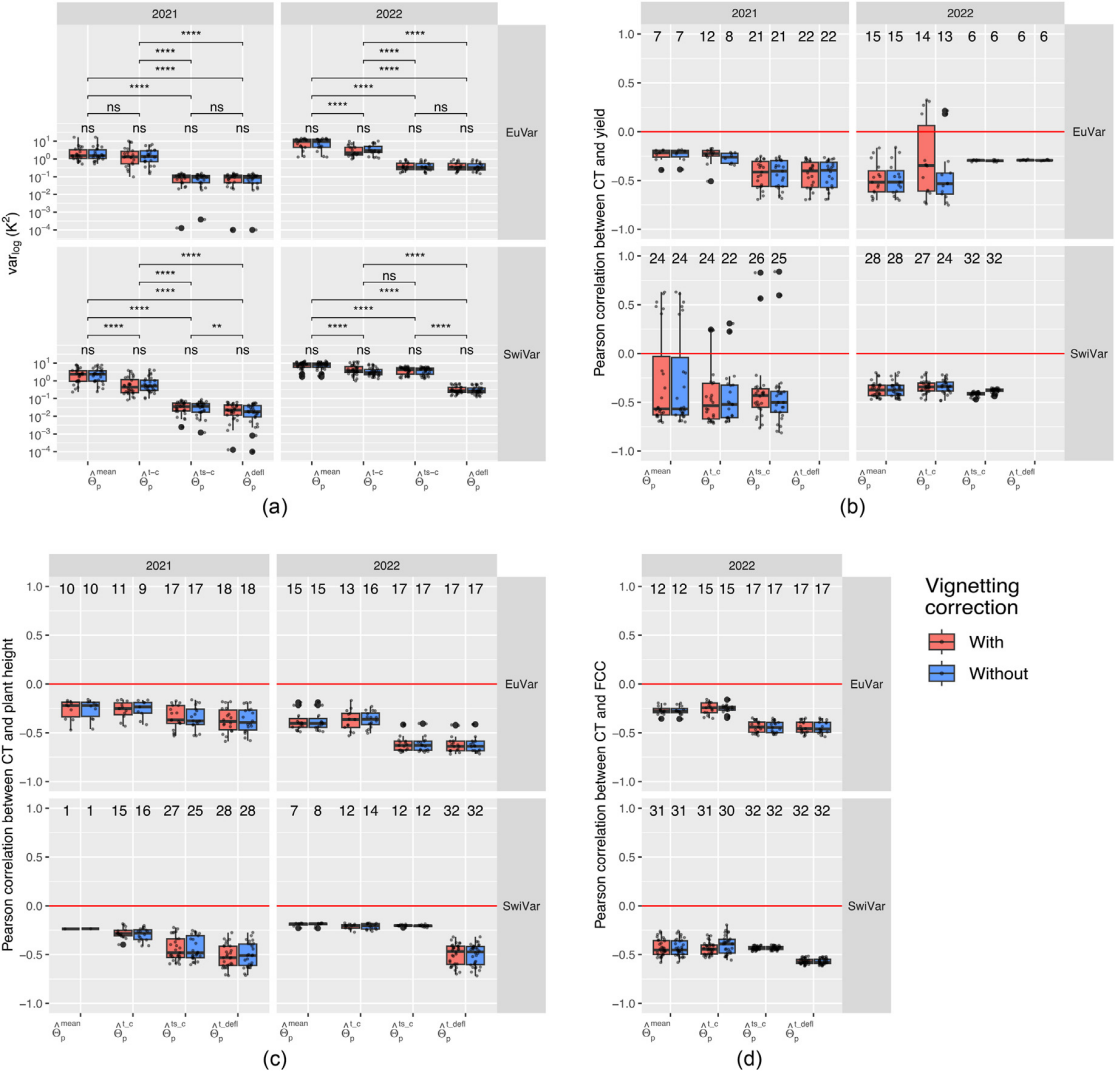
Variance of CT estimates after different corrections steps and relationship between CT and in-field reference measurements: (a) Comparison of the variance of uncorrected plot-wise estimates (Eq. (2)) over all flights with CT estimates after correcting with the mixed model (Eq. (3), Eq. (4) & Eq. (5)). Significant differences between correction steps are indicated based on a pair-wise *t*-test. Significance levels: ns: p > 0.05; ∗: p ​≤ ​0.05; ∗∗: p ​≤ ​0.01; ∗∗∗: p ​≤ ​0.001; ∗∗∗∗: p ​≤ ​0.0001. Note that a logarithmic scale is used! The CT estimates without and with correction were also correlated to in-field reference measurements, namely (b) yield, (c) plant height and (d) FCC. Just correlations significant at p ​≤ ​0.01 are shown. The number above the boxplots indicates the number of significant correlations included in the respective box plots.

When correcting plot-wise CT estimates for temporal effects $\left({\hat{\theta }}_{p}^{t\text{\_}c}\right)$, temporal and spatial effects $\left({\hat{\theta }}_{p}^{ts\text{\_}c}\right)$ and finally also deflating treatment effects $\left({\hat{\theta }}_{p}^{t\text{\_}defl}\right)$, the variance of the adjusted plot estimates was constantly decreasing (Fig. 4a). The variance of ${\hat{\theta }}_{p}^{t\text{\_}defl}$, which still comprised genotypic variance, variance of genotype-treatment interactions, and plot effects, was orders of magnitude smaller than the initial variance of uncorrected plot estimates ${\hat{\theta }}_{p}^{\mathrm{mean}}$. The mean variance decreased from 2.74 ​K^2^ to 0.09 ​K^2^ for EuVar21 and from 8.40 ​K^2^ to 0.42 ​K^2^ for EuVar22. For SwiVar21, variance decreased from 2.75 ​K^2^ to 0.02 ​K^2^ and from 7.68 ​K^2^ to 0.32 ​K^2^ for SwiVar22.

The greatest variance reduction occurred with the temporal and spatial correction, after which the variance was below 0.5 ​K^2^, except for SwiVar22. The variance was similar for ${\hat{\theta }}_{p}^{ts\text{\_}c}$ and ${\hat{\theta }}_{p}^{t\text{\_}defl}$ for all experiments but for SwiVar22, where the variance decreased a lot by treatment deflation, indicating a mild treatment effect for EuVar21, EuVar22 and SwiVar21 but a strong treatment effect for SwiVar22.

### 3.6 Impact of correcting CT for non-geometric trends on correlation between CT and phenotypic traits

**Fig. 5:**
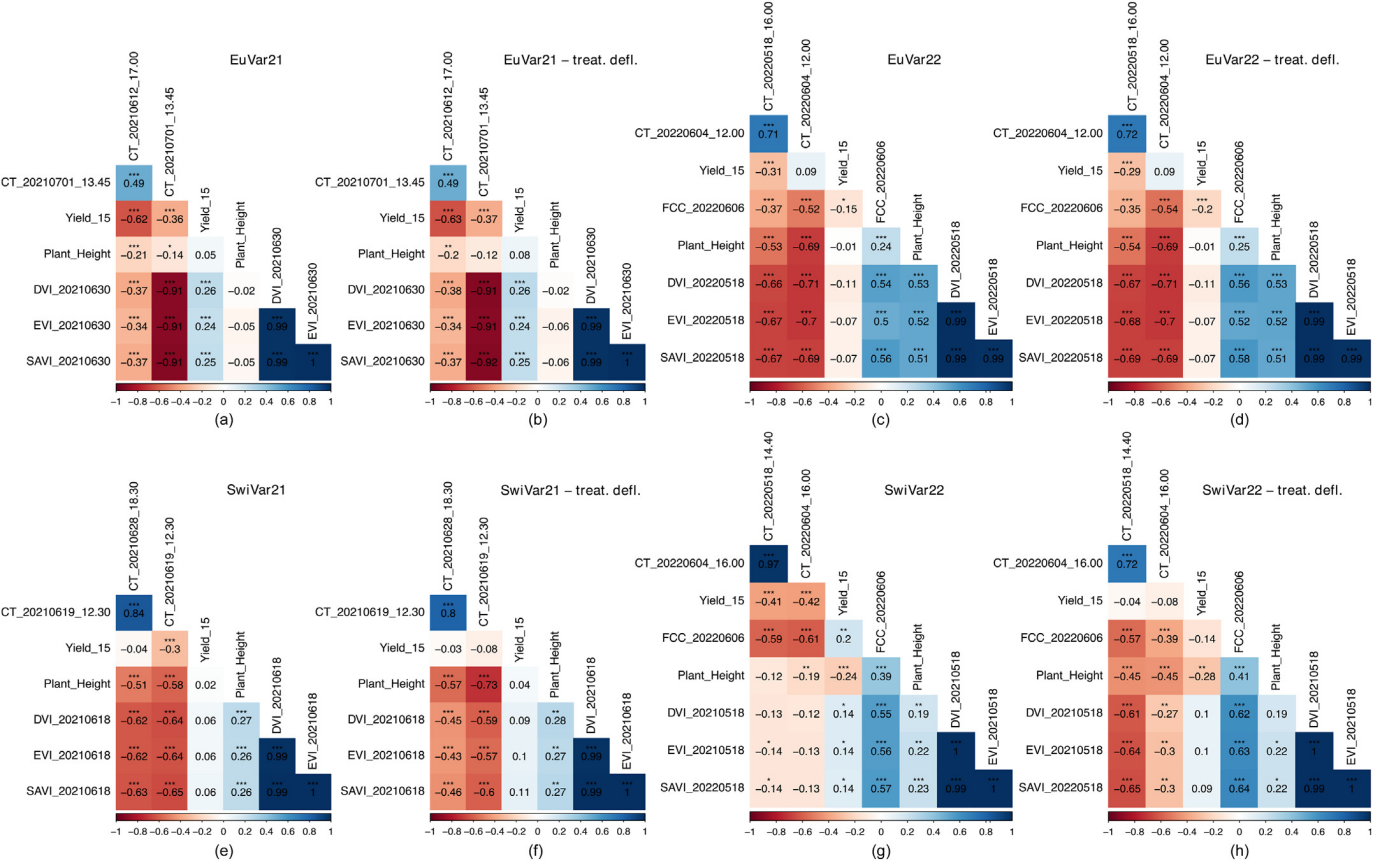
Pearson correlation of genotypic CT with genotype specific in-field reference measurements for EuVar (a–d) and SwiVar (e–h). For each experiment in each year, CT of two distinct dates was correlated with yield at 15 ​% gravimetric water content, plant height and three multispectral indices DVI, EVI and SAVI. Correlations were calculated on CT after a spatial and temporal correction according to Eq. (4) (a, c, e, g) and after deflating treatment effects on CT estimates as well as on reference measurement by subtracting mean treatment effects (b, d, f, h). Dates and flight times are indicated for CT measurements and dates for multispectral measurements and FCC estimates. FCC estimates were just done in 2022.

Yield, plant height, four multispectral indices (DVI, EVI, NDVI, SAVI) and in 2022 also FCC were measured as phenotypic reference traits, as they represent possible physiological sources of CT variance for EuVar (Fig. S21 and S22) and SwiVar (Fig. S23 and S24). In EuVar21, senescence ratings were performed. Flag leaf rolling was rated in 2022 as an indicator of drought stress. In-field reference measurements were compared with CT of corresponding flights by Pearson correlation. Uncorrected CT values were correlated with the uncorrected reference measurements. Corrected CT was correlated with corrected reference measurements and treatment deflated CT was correlated with treatment deflated reference measurements. For yield, plant height, and FCC, a general overview of the correlations with CT is presented in Fig. 4b–d. For each trial in each year, two flights conducted at two distinct dates were analyzed for each experiment before treatement deflation (Fig. 5a, c, e, g) and after (Fig. 5b, d, f, h).

#### 3.6.1 Correlation between CT and yield

Yield at 15 ​% gravimetric water content was correlated with CT in conditions with and without water limitation and in the presence of weaker and stronger treatment effects. Significant correlations tended to be more consistent over all flights after applying different corrections.

Correlations were increased by the different corrections in the wet year 2021 for the relatively heterogeneous set of genotypes of EuVar21. Uncorrected CT ${\hat{\theta }}_{p}^{\mathrm{mean}}$ was significantly correlated with yield only for 7 out of 22 flights and correlations were negative and weak to moderate (Fig. 4b). After correction $\left({\hat{\theta }}_{p}^{ts\text{\_}c}\right)$, correlations were weak to strong and significant for all 22 flights (p ​≤ ​0.01).

For the same genotypes in the dry year (EuVar22), uncorrected CT for 15 out of 17 flights was significantly and negatively correlated with yield with weak to strong correlations. After temporal and spatial correction, only 6 flights showed a weak significant correlation with yield. Therefore, the correlation between CT and EuVar22 yield was mainly driven by spatial trends. For both trials of EuVar, deflation of treatments $\left({\hat{\theta }}_{p}^{t\text{\_}defl}\right)$ had little effect.

For the less heterogeneous genotypes of SwiVar, the trends were similar for both years. Initially, SwiVar21 and SwiVar22 showed a very broad range of correlations between yield and uncorrected CT values ${\hat{\theta }}_{p}^{\mathrm{mean}}$. After correction $\left({\hat{\theta }}_{p}^{ts\text{\_}c}\right)$, more correlations were significant and mostly negative, except for SwiVar21, where two correlations were positive. For SwiVar22, all 32 flights were significantly and negatively correlated with yield. However, after deflating the treatment effects $\left({\hat{\theta }}_{p}^{t\text{\_}defl}\right)$, correlations were no longer significant for SwiVar in both years.

The differences between the data with and without vignetting correction were small, except for the ${\hat{\theta }}_{p}^{ts\text{\_}c}$ values of EuVar22, where correlations with yield were relatively random. To have a more robust estimate of the reliability of these correlations, CT was also estimated based on all flights within campaigns (Fig. S25) and then correlated with yield. The general pattern of correlations was similar to that based on individuals flights.

Correlations of selected flights (Fig. 5) are in accordance with this general pattern with strongest and most highly significant correlations for EuVar21 (p ​≤ ​0.001). The correlation in SwiVar was strongly driven by treatment effects, and the correlations were no longer significant after deflating treatment effects.

#### 3.6.2 Correlation between CT and plant height

Significant correlations between CT and plant height were negative for all flights (Fig. 4c). For all experiments, the correlations became stronger and more flights became significantly correlated with plant height after the corrections. After correcting for temporal, spatial and treatment effects, all flights were significantly correlated with plant height except four EuVar21 flights. Deflating treatment effects did not change the correlations much for EuVar, but led to more negative correlations for the less heterogeneous genotypes of SwiVar in both years, but especially during the hot season of SwiVar22.

Looking at selected flights (Fig. 5), the correlation between CT and plant height was weaker and less significant in the trial with heterogeneous genotypes during the wet year (EuVar21), compared to all other trials, which showed all highly significant correlations (p ​≤ ​0.001), except for SwiVar22, where this was the case only after treatment deflation.

#### 3.6.3 Correlation between CT and FCC

As for plant height, the correlations with FCC became more significant and stronger with the corrections applied (Figs. 4d and 5). For EuVar22 and for SwiVar22, CT of all flights was significantly correlated with FCC after temporal and spatial correction. For EuVar22, treatment deflation did not much change the correlations. For SwiVar22, correlations became stronger with treatment deflation, indicating a genotypic effect as the driver of the correlation between CT and FCC, partially masked by a strong treatment effect.

#### 3.6.4 Correlation between CT and multispectral vegetation indices

VIs were negatively correlated with CT for all trials (Fig. 5) and the correlations were highly significant (p ​≤ ​0.001) except for SwiVar21 before treatment deflation (p > 0.01). Correlations were always higher with the CT measurements taken closer to the date of the VI measurements.

#### 3.6.5 Impact of flag leaf rolling on CT

**Fig. 6:**
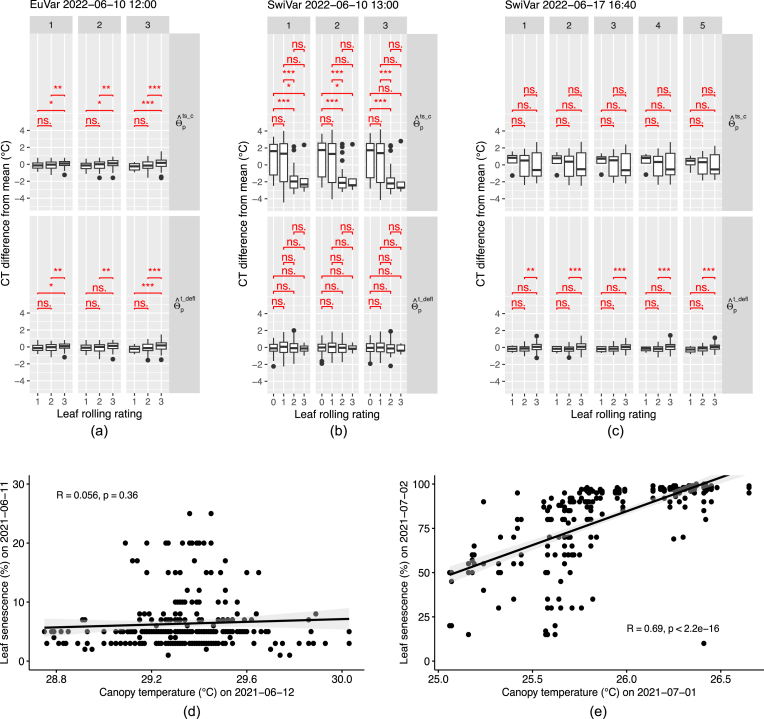
Impact of flag leaf rolling and senescence on CT. Corrected CT differences from mean were grouped for campaigns on specific dates and flight times by their flag leaf rolling rating for EuVar on 2022-06-10 (a) and SwiVar on 2022-06-10 (b) and on 2022-06-17 (c) before $\left({\hat{\theta }}_{p}^{ts\text{\_}c}\right)$ and after $\left({\hat{\theta }}_{p}^{t\text{\_}defl}\right)$ applying a treatment deflation on CT estimates. The numbers above the individual columns indicate the flight number of the flights within the campaigns of CT measurements. For EuVar on 2022-06-10 and SwiVar on 2022-06-17, all ratings were larger than 0. Leaf rolling ratings were conducted on the same day as flights or the day before. The significance of differences between groups of leaf rolling ratings was highlighted in red. Significance levels: ns: p > 0.05; ∗: p ​≤ ​0.05; ∗∗: p ​≤ ​0.01; ∗∗∗: p ​≤ ​0.001. Senescence ratings of EuVar21 for 2021-06-11 were compared with the CT of 2021-06-12 ​at 17.00 (d) and the senescence ratings for 2021-07-02 were compared with the CT of 2021-07-01 ​at 13.45 (e).

When grouping CT estimates according to flag leaf rolling ratings of the dry year 2022, significant differences of CT could be observed for some flights. For EuVar22 flights on 2022-06-10 ​at 12:00 (Fig. 6a), CT was significantly different between leaf rolling rating groups for CT estimates before and after applying a treatment deflation on CT values (${\hat{\theta }}_{p}^{ts\text{\_}c}$ & ${\hat{\theta }}_{p}^{t\text{\_}defl}$). For SwiVar flights on 2022-06-10 ​at 13:00 (Fig. 6b), differences were significant before treatment deflation $\left({\hat{\theta }}_{p}^{ts\text{\_}c}\right)$ but not after $\left({\hat{\theta }}_{p}^{t\text{\_}defl}\right)$ and lower flag leaf rolling ratings were associated with higher temperatures. The differences were only significant after treatment deflation for the flights on 2022-06-17 ​at 16:40 (Fig. 6c) but not before. The differences between the flag leaf rolling rating groups after treatment deflation were generally small (< 0.40 ​K). For most other dates, differences were not significant (Figs. S32–S34).

#### 3.6.6 Impact of senescence on CT

Flag leaf senescence was just rated for the two dates of EuVar21. The senescence ratings for 2021-06-11 were compared with the CT of 2021-06-12 ​at 17.00 (Fig. 6d) but the correlation was not significant. The senescence ratings for 2021-07-02 were strongly correlated (r ​= ​0.69, p ​≤ ​0.001) with the CT of 2021-07-01 ​at 13.45 (Fig. 6e).

### 3.7 Phenotypic correlations between reference measurements

Correlations between in-field reference measurements with CT were discussed above, yet possible correlations between reference measurements as summarized in Fig. 5 must also be considered.

Plant height and yield were never correlated except for weak but significant correlations in SwiVar22 prior to treatment deflation (p ​≤ ​0.01).

Yield was only weakly correlated with VIs for EuVar21 and for SwiVar22 before treatment deflation (p ​≤ ​0.001), but significant correlations were always weaker than correlations between yield and CT for corresponding dates.

FCC and yield showed a weak but significant correlation (p ​≤ ​0.001) in EuVar22 and in SwiVar22 before treatment deflation (p ​≤ ​0.01).

### 3.8 Spatial CT trends in the field

**Fig. 7:**
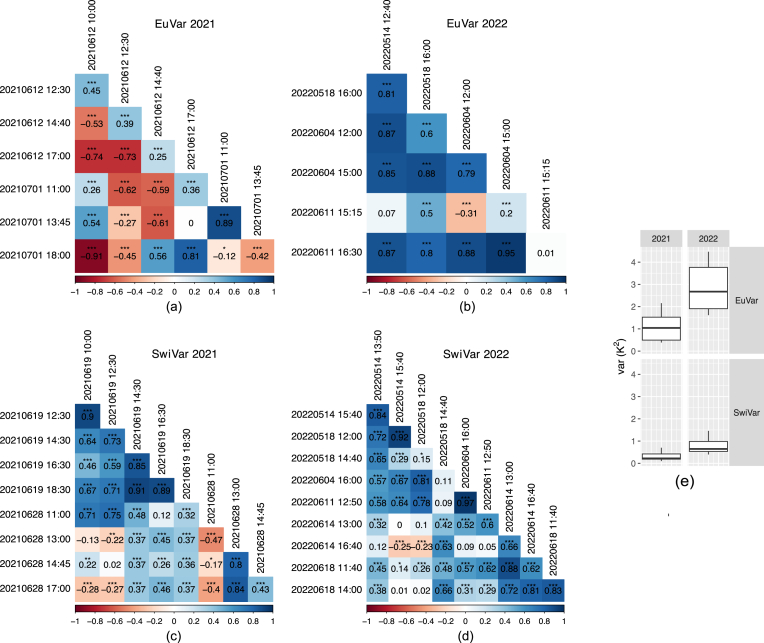
Pearson correlations between estimates of spatial trends ${\hat{\theta }}_{r_{\left(p\right)}c_{\left(p\right)}}$ for individual campaigns. Spatial trends were estimated according to Eq. ​10 for (a) EuVar21, (b) EuVar22, (c) SwiVar21 and (d) SwiVar22. Estimates were based on all flights within individual campaigns. The variance of estimates of spatial trends is summarized in (e). Significance levels:∗: p ​≤ ​0.05; ∗∗: p ​≤ ​0.01; ∗∗∗: p ​≤ ​0.001.

Based on estimates of single flights, the spatial field trend estimates ${\hat{\theta }}_{r_{\left(p\right)}c_{\left(p\right)}}$ were not consistent. The sign of the correlations between flights changed randomly, (Fig. S26 - Fig. S31). Spatial trend estimates based on all flights within campaigns appeared random for EuVar21 (Fig. 7a, Fig. S36a and S38) but more consistent for EuVar22, SwiVar21, and SwiVar22. For EuVar22 (Fig. 7b, Fig. S36b and S39) spatial field trends of campaigns were positively correlated except for the campaign on 2022-06-11 ​at 15:15 and correlations were highly significant. SwiVar21 flights (Fig. 7c, Figs. S37a and S40) showed moderate to very strong correlations within the 2021-06-19 flights. Within 2021-06-28, the correlations were positive and negative, while the positive correlations were stronger and more significant. The correlations between flights on 2021-06-19 and 2021-06-28 were positive for 16 out of 20 correlations and were weak to strong and highly significant in most cases. The four negative correlations were very weak to weak and significant at p ​≤ ​0.001 just in two cases. Within SwiVar22 (Fig. 7d, Fig. S37b and S41), the correlations ranged from strong to very strong (p ​≤ ​0.001) within days and from moderate to strong between different days. Weaker correlations were often not significant at p ​≤ ​0.05. Two correlations were negative but significant at p ​≤ ​0.001.

The variance of the spatial trend estimates within flights $var\left({\hat{\theta }}_{r_{\left(p\right)}c_{\left(p\right)}}\right)$ was much stronger in 2022 compared to 2021 for both trials (Fig. 7e). The mean $var\left({\hat{\theta }}_{r_{\left(p\right)}c_{\left(p\right)}}\right)$ was 1.09 ​K^2^ for EuVar21 and increased to 2.87 ​K^2^ for EuVar22. The mean $var\left({\hat{\theta }}_{r_{\left(p\right)}c_{\left(p\right)}}\right)$ was lower in SwiVar but also increased from 0.32 ​K^2^ for SwiVar21 to 0.76 ​K^2^ for SwiVar22.

### 3.9 PLSR modeling of TIR residuals to better understand geometric sources of variance of apparent CT

#### 3.9.1 TIR residuals and geometric trends

**Fig. 8:**
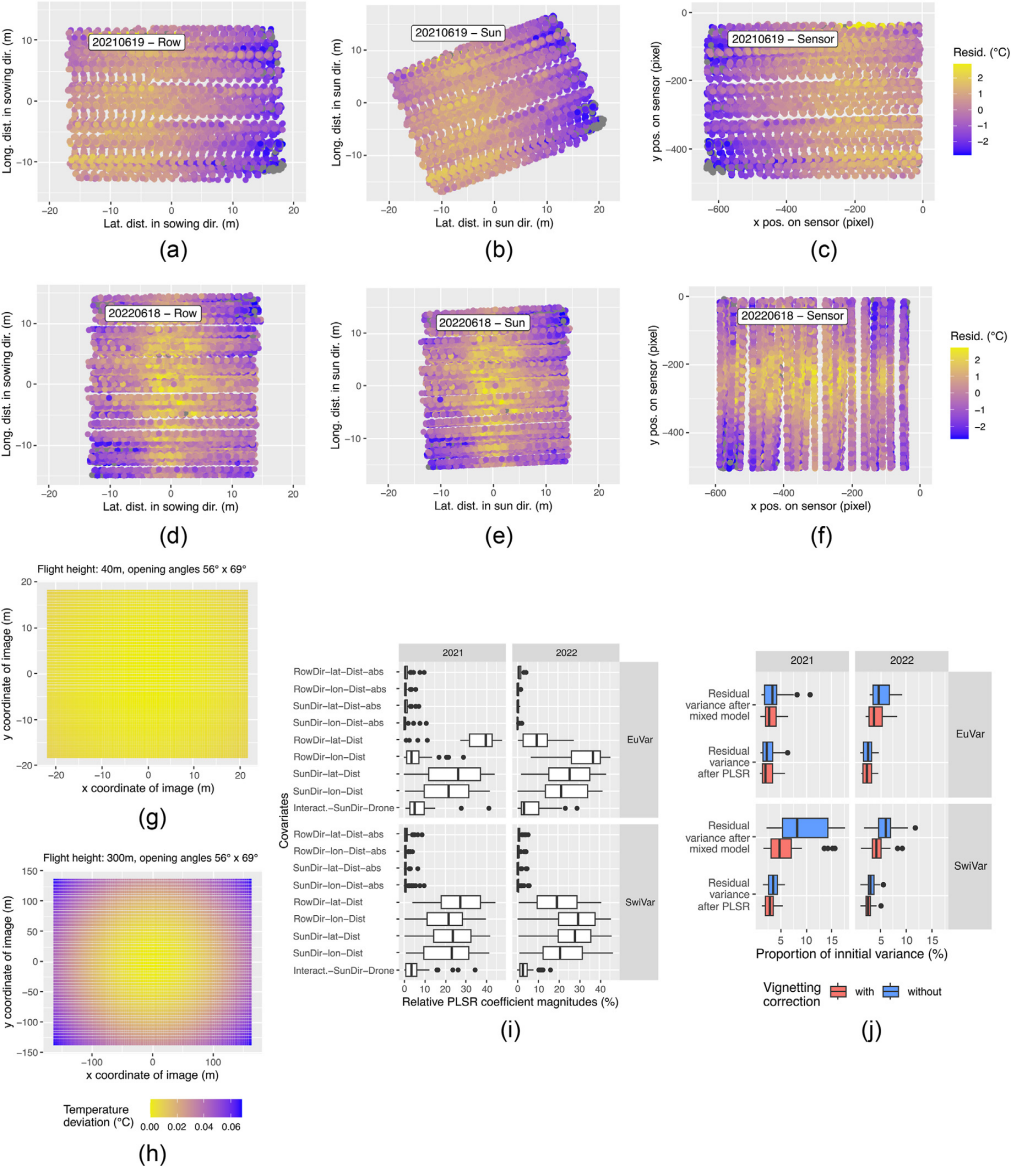
Geometric trends of CT estimates of first flights of SwiVar campaigns on 2021-06-19 ​at 12:30 (a–c) and on 2022-06-18 ​at 11:40 (d–f). CT residuals of the mixed model (Eq. (1)) are plotted with respect to lateral and longitudinal distance of the plot seen from the drone in sowing row direction (a & d), sun direction (b & e) and the position of the plot center on the focal plane array of the TIR sensor, *i.e.* the x/y coordinates of the thermal images (c & f). A theoretical atmospheric effect is shown for two different flight heights (g) 40 ​m and (h) 300 ​m. (i) Shows the PLSR coefficients of the 9 selected linearized geometric covariates which indicate the relative importance of the covariates in PLSR modeling to explain the variance of the CT residuals after the mixed models. (j) Summarizes the variance after the mixed models (multiple for each plot in each flight) and after PLSR modeling expressed as % of initial variance.

After pre-processing with the mixed model in ASReml, the residuals were analyzed for geometric patterns. Looking, for example, on the residuals of the flight of the SwiVar campaign on 2021-06-19 ​at 12:30 (Fig. 8a - c), a gradient along the lateral “distance in direction of sowing rows” (Fig. 8a) can be seen. The dimensions “distance in direction of sun” (Fig. 8b) and “distance on the sensor” (Fig. 8c) showed very similar patterns and the main difference was a rotation around the origin of the respective dimensions. For the first flight of the SwiVar campaign on 2022-06-18 ​at 11:40 (Fig. 8d - f), distinct patterns can be seen with respect to the dimensions “distance in direction of sowing rows” (Fig. 8d), “distance in direction of sun” (Fig. 8e) and “distance on the sensor” (Fig. 8f). The residuals were more positive below the camera and more negative with more oblique viewing geometries and patterns were very similar again between the dimensions with a rotation around the origin. Although these patterns were not always the same between the flights, they were always very similar between the three dimensions of one flight. Also, after vignetting correction, the patterns remained very similar to patterns before vignetting correction (not shown).

The theoretical atmospheric effect was almost zero for a flight height of 40 ​m (Fig. 8g) but became larger at a flight height of 300 ​m (Fig. 8h). The pattern at flight height 300 ​m was very similar to the geometric trends at 2022-06-18 (Fig. 8d - f) but also to the vignetting effect (Fig. S3). While the real atmospheric effect could not be described within this study, this demonstrates the point-symmetric nature of this effect but also its negligible order of magnitude at low flight heights.

### 3.10 PLSR modeling of geometric CT trends

The residuals *e*_ijknp_ of the mixed model (Eq. (1)) were used as input of the PLSR model. Table 4 summarizes how much of the variance of *e*_ijknp_ within single flights could be explained using geometric covariates in PLSR.

**Table 4:** Explained variance of residuals *e*_ijknp_ by PLSR fitting after pre-processing with the mixed model (Eq. (1)). PLSR fitting was done with all 20 lineralized covariates and a reduced set of nine selected covariates. Mean and median values were calculated over all PLSR models of the two experiments EuVar and SwiVar for data with and without vignetting correction (VC) over the two years.

	Number of covariates		Explained variance of residuals (%)			
			2021		2022	
			without VC	with VC	without VC	with VC
EuVar	20	mean	30.9	20.8	50.8	42.9
		median	30.4	21.5	47.5	39.2
	9	mean	24.4	17.8	48.6	41.1
		median	20.3	18.6	45.9	37.6
SwiVar	20	mean	62.6	45.3	51.3	40.9
		median	65.3	43.9	56.0	45.6
	9	mean	57.6	41.9	47.9	37.7
		median	59.2	41.1	52.3	42.3

Of the 20 initial covariates included in the PLSR models (Table 3), 9 were selected in a supervised selection for use in further processing. The relative PLSR coefficient magnitudes *β*_i_ of the selected covariates are shown in Fig. 8i. The four covariates “RowDir-lat”, “RowDir-long”, “SunDir-lat” and “SunDir-lon” were the most important in PLSR, followed by “Interact.-SunDir-Drone”. The absolute values of the four covariates (“RowDir-lat-abs”, “RowDir-long-abs”, “SunDir-lat-abs” and “SunDir-lon-abs”) were less important in PLSR for most flights with values around 0 ​%. However, for some flights, especially in 2021, they reached values of up to 10 ​%.

The median values of the explained variance ranged from 20.3 ​% to 59.2 ​% when just including 9 covariates and not applying vignetting correction. They were generally highest in SwiVar21 while they were lowest in EuVar21. EuVar22 and SwiVar22 showed intermediate values. When only using 9 instead of 20 covariates, the explained variance was 4.0 ​% lower on average. The explained variance without ex ante vignetting correction was on average 10.9 ​% higher compared to data with vignetting correction applied. The differences without and with vignetting correction were greater for SwiVar than for EuVar.

Fig. 8j compares the residual variance of mixed models and PLSR to initial variance of CT values and variance of initial CT values corresponds to 100 ​%. The proportion of variance explained with mixed models was always larger when ex ante vignetting correction was applied, while the variance of the initial CT values was very similar (Fig. 4a). This holds also true for the variance explained after PLSR but the differences between data with and without vignetting correction became smaller. The mean proportion of residual variance after mixed models ranged from 2.98 ​% to 9.61 ​%. After PLSR, the mean proportion of residual variance ranged from 2.46 ​% to 3.51 ​%, *i.e.* by combining mixed models and PLSR, 97.54 ​% – 96.49 ​% of initial CT variance could be explained on average. Details for the reduction in variance of single flights are shown in Fig. S42 - Fig. S45.

### 3.11 Reference measurement to better understand the sources of variance in apparent CT

#### 3.11.1 Fan experiment to determine the influence of wind

**Fig. 9:**
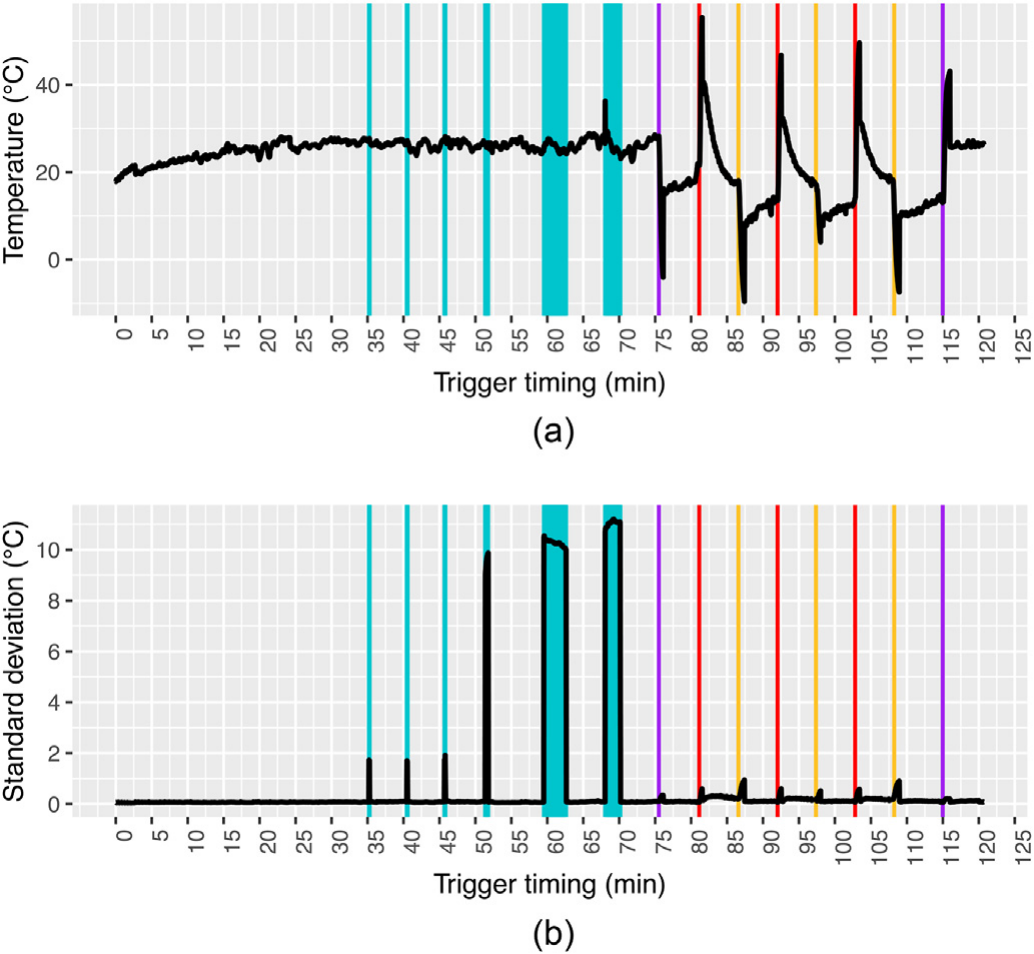
TIR drift (a) and standard deviation of pixels-wise temperature on the PVC sheet (b) during the fan experiment. During the stabilization period, warm objects were introduced into the FOV three times (first three vertical blueish shadings), and then hot objects were introduced into the FOV for three times (subsequent three larger shadings). At about 75 ​min, the heating lamp was turned on (first vertical purple line). The fan was then turned on (red lines) and off (yellow lines) three times before the lamp was turned off (second vertical purple line).

The fan experiment showed a strong reaction of the sensor to heating and cooling (Fig. 9). The apparent temperature of the PCV sheet dropped immediately by more than 20 ​°C upon switching on the lamp and rose again to a temperature of about 10 ​°C below the previous temperature. During the next 15 ​min, it slowly increased. As soon as the fan was turned off, the temperature rose by more than 30 ​°C and immediately decreased again and continued to decrease for 5 ​min until the fan was turned off and the temperature dropped again until the fan was turned on again. The same pattern was repeated three times until the lamp was finally turned off and the temperature stabilized anew. Strong temperature gradients between monitored objects themselves did not cause any drift. The introduction of warm and hot objects did increase the standard deviation of the pixel-wise temperature as long as the objects were within the FOV but did not appear to cause a drift of the apparent temperature or an increased standard deviation for any longer than the period during which disturbance objects were present inside the FOV.

#### 3.11.2 Qualitative demonstration of impact of apparent soil cover on CT

**Fig. 10:**
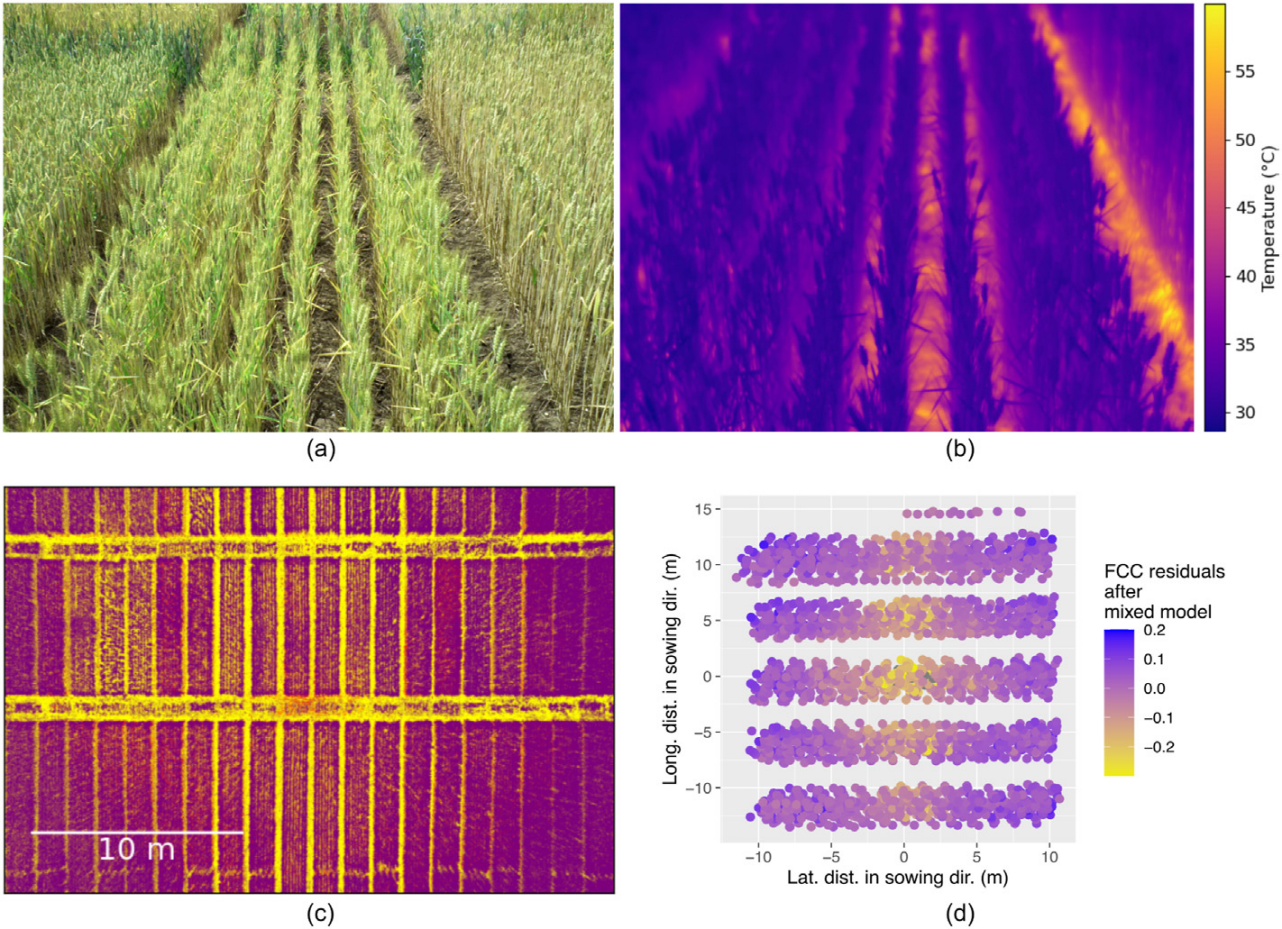
FCC trends in relation to viewing geometry: The same scenery is shown on an RGB image (a) and a TIR image (b). This shows how the soil is warmer than the plants. To demonstrate how the apparent fractional canopy cover (FCC) changes with viewing geometry, RGB images were labeled in Ilastik software to segment images into plant (purple) and background (yellow) (c). The resulting images were analyzed by the multi-view method to get FCC for each plot in each image. The FCC values were fitted with a mixed model (Eq. (1), but for CT instead of FCC) for design factors. The residuals of the model are shown in (d) in relation to the position of the plot relative to the sowing row direction for SwiVar22.

For most situations, soil was warmer than the vegetation which was especially evident when looking into the rows perpendicularly (*e.g.* Fig. 10a & b). From an oblique viewing angle, the FCC decreased and so did the average apparent CT in the respective area.

#### 3.11.3 Multi-view residuals of FCC from RGB data to demonstrate viewing-geometry dependency of CT

The apparent FCC showed a distinct pattern with a lower apparent FCC in the center and a higher apparent FCC toward the edges of the images (Fig. 10c) which is related to the more oblique viewing angles. After fitting the FCC values for design factors in a mixed model (Eq. (1), but for CT instead of FCC), the residuals showed a distinct pattern with regard to position relative to row direction (Fig. 10d). They were lowest when following a line parallel to row direction directly below the drone (lateral distance in the direction of sowing ​= ​0). When diverging perpendicularly from this line in both directions (*i.e.* with increasing lateral distance perpendicular to the direction of sowing), the residuals became more positive, *i.e.* FCC increased. A similar yet less distinct effect could be observed along this line with increasing residual values when diverging from the position on the soil directly below the drone (with increasing longitudinal distance parallel to direction of sowing). Areas with low FCC coincided with warm areas, and spatial trends were often very similar between the two traits (cf. Fig. 8, Fig. 10d–f).

## 4 Discussion

This study used the multi-view approach [29] to discuss the manifold sources of variance in airborne thermal imaging, based on data from two very different wheat variety testing trials followed over two seasons, characterized by very contrasting meteorological conditions. The discussion of the different sources is structured according to the primary type of correction (Table 1).

### 4.1 Temporal correction of CT

Temporal trends contributed the most to the total variance of CT estimates. Fig. 3a illustrated the magnitude of temporal trends, which can be several times larger than genotype-specific differences (*e.g.,* [[20], [27], [29]]). Temporal correction reduced the variance of CT estimates the most (Fig. 4a) which is in line with Wang et al. [27]. This demonstrates the importance of proper handling of temporal trends in thermal measurements, as has been highlighted in several publications (*e.g.,* [[20], [27], [29], [30], [32]]). Wang et al. [27] elaborated on the distinction between thermal drift and the temporal variation of land surface temperature (LST). Thermal drift is caused by the thermal camera when the temperatures of FPA, lens, and camera body change. The wind on the sensor cools them and exposure to sunlight as well as the sensor's electronic heats them, leading to fluctuation temperature readings even when facing toward a target with an actual constant temperature (*e.g.,* [[12], [20], [27], [29], [62]]). This interaction was confirmed for the sensor used in this study with a fan experiment (Fig. 9). In accordance with findings in Kelly et al. [20], the warming of the sensor led to a decrease in the apparent temperature of the target and vice versa. As internal processes of TIR cameras are proprietary information of the manufacturers, the reasons for this are difficult to determine [20,63]. The thermal signal reacted within seconds after a change of wind conditions (fan) or thermal radiation (heating lamp). In contrast to thermal drift, temporal variation corresponds to actual changes in the temperature of a given target that can be caused by wind, changing air temperature, VPD, solar illumination, changing water status of the plant and the plants physiological response to such changes (*e.g.,* [[3], [4], [15], [21], [27]]). The impact of temporal variation was reduced in this study by flying in weather conditions that were rather stable throughout single flights (Fig. S6 and S7) [20]. Nevertheless, also in stable conditions, LST changes, but these changes are comparably slow and if measurements are taken within a short interval, *e.g.*, within 30 ​min, the temporal variation in LST is relatively low [27]. A typical flight time in this study was 7–9 ​min, a 3 flight campaign lasted about 25 ​min, and therefore a large proportion of temporal trends can be assumed to be thermal drift, and temporal variation contributed relatively little to total variance of CT estimates.

### 4.2 Spatial correction of CT

Thermal imaging was proposed to estimate spatial field heterogeneity caused, for example, by variability of soils, soil water content, or soil-borne pathogens, and to improve the interpretability of other phenotypic measurements (*e.g.,* [[2], [6], [12]]). In contrast to hand-held infrared thermometers, many experimental plots and larger areas can be measured simultaneously and repeatedly in a short period with airborne thermography. Handheld infrared thermometers are also prone to thermal drift, but with just one measurement taken at a time, the temporal and spatial trends are challenging to separate from each other in a statistical analysis [6]. Revisiting the same spot multiple times in a short interval (*e.g.* 30 min) improves the estimation of the real relative temperature of the spot and thus of spatial trends, since the temporal variation of the CT can be assumed to be relatively small and temporal trends are mainly thermal drift [27]. When working with uncorrected images in orthomosaic approaches, each plot is measured multiple times. The temporal and spatial effects are reduced by leveling them out in orthomosaic blending. Perich et al. [15] accounted for the remaining temporal and spatial variance together in a mixed model, and they stated that it remains challenging to unravel the two. Multi-view offers an opportunity to alleviate this limitation as measurements are analyzed individually [29], but as shown in this study, a spatial trend estimate based on a single flight showed little reliability (Fig. S28 - Fig. S31). As claimed by Wang et al. [27], increasing the number of observations per plot led to a more consistent estimation of the spatial trends (Fig. 7a–d). To further improve the estimation of spatial trends, it is proposed to conduct at least two flights over the field with different flight paths, orthogonal to each other. This reduces the probability of artifacts due to the repeated occurrence of similar temporal patterns when following the same flight plan.

In 2022, the spatial trend ${\hat{\theta }}_{r_{\left(p\right)}c_{\left(p\right)}}$ was more pronounced than in 2021. Thus, the variance of the spatial trend was greater in 2022 than in 2021 (Fig. 7e), indicating a stronger expression of the spatial trend. 2021 was a wet year and a sufficient water supply can be assumed throughout the growing season. Spatial trends were therefore relatively weak. Such weak trends are more difficult to reproduce, as little differences in the estimation lead to different trends. In such homogeneous conditions, the multi-view approach might fail to detect the weak spatial trends reliably. At the same time, the correct estimation of weaker trends is also less important because their impact on final results becomes negligible. 2022 was hot and dry, and spatial trends in water status were observed in the field. A more pronounced spatial trend can be estimated more easily and reliably.

However, simultaneous accounting for temporal and spatial trends was shown to lead to highly consistent CT estimates even when based only on a single flight [29].

### 4.3 CT variance reduction by temporal and spatial trends

After the temporal and spatial correction, the experimental effects remained, *i.e.*, the effects of genotypes and treatments, as well as the effects of viewing geometry.

The added variance of these effects was much smaller than the initial variance of the temperature estimates, with the exception of SwiVar22, which showed a strong treatment effect (Fig. 4a). Agricultural research is usually interested in the effects of genotypes and treatments. This shows the importance of reducing the effects of unwanted sources of variance. Only through the appropriate consideration of large confounding influences, more subtle effects actually under observation within an experimental setup can truthfully be estimated [11].

### 4.4 Correlation of CT with in-field reference measurements of phenotypic traits

In accordance with the literature [3,4], yield and CT were negatively correlated in conditions without water limitation. This was only the case in 2021 as 2022 was a hot and dry season. The correlations were stronger and more significant in EuVar21 compared to SwiVar21. While the effect of fertilizer application in SwiVar21 was rather small, it appeared to be the main driver of the correlation between corrected CT and yield. In the EuVar trial, a relatively diverse set of European genotypes was tested, while in SwiVar, varieties of the Swiss variety list and candidates for registration in the variety list were tested. It can be assumed that the phenotypic variability between the varieties was greater in EuVar than in SwiVar. With more pronounced differences between estimates, stronger correlations are more easily achieved.

There was a consistently negative correlation between CT and plant height. This could in part be caused by effects related to canopy architecture, *e.g.* increased LAI and a stronger exposure to wind (*e.g.,* [27]), but also by genetic co-locations of quantitative trait loci for CT and plant height (*e.g.,* [3]). The correlations were more pronounced in SwiVar after treatment deflation, indicating a masking effect of fertilizer treatment on the genotypic correlation between CT and plant height.

Although just measured in 2022, the trends for FCC were similar to those of plant height, with stronger correlations after treatment deflation. The constant correlation between FCC and plant height also indicates that they can be interlinked. Furthermore, with decreasing FCC, the effect of mixed pixels can be expected to increase, especially if the GSD is larger than the size of the plant organs [64], shifting the CT estimate toward the temperature of the soil background.

The flag leaf rolling is a protective mechanism of wheat to reduce transpiration losses. It reduces the amount of incident radiation intercepted by the plant and traps air within the leaf, reducing the VPD at the border layer [8]. It was used as an indicator of the level of drought and heat stress to which the wheat was exposed. Although CT differences between groups of different flag leaf rolling ratings were significant for some dates, these differences remained relatively small (< 0.40 ​K) after treatment deflation. Differences before treatment deflation were large for SwiVar on 2022-06-10 (Fig. 6b) and lower flag leaf rolling ratings were associated with higher CT estimates, which is counter-intuitive. To understand this, the interaction between CT estimates, water use, and above-ground biomass must be analyzed. In 2022, it was evident from field observations that the above-ground biomass in the unfertilized part of SwiVar was much lower than in the fertilized part. The lower biomass was confirmed by reference measurements, as FCC but also multispectral indices that approximated above-ground biomass and LAI were lower in the unfertilized part (Fig. S24). At the same time, flag leaves expressed stronger rolling in the fertilized part compared to the unfertilized part (Fig. S35), indicating the plants experienced a stronger water deficit in the fertilized part [8]. The lower biomass presumably led to a lower total transpiration in the unfertilized part and saved soil water, which in turn allowed plants to maintain unrolled leaves longer into the season compared to the fertilized part, where available water was exhausted earlier. This illustrates well the complex interactions between phenotypes, water status, transpiration, and CT. At the same time, this highlights the importance of environments for the contextualization of the expression of CT as a trait. In 2021 almost the same set of genotypes was sown as in 2022 and the treatments were identical, but led to a much more pronounced treatment effect in 2022 with lower FCC, above-ground biomass and LAI.

The correlation between CT estimates and reference measurements was strongest between CT and multispectral vegetation indices (Fig. 5). This correlation was strongest in EuVar21, when the correlations between CT and yield were also strongest. CT was often negatively correlated with yield and plant height, but yield and plant height were not correlated except for a weak correlation in SwiVar22. The impact of the treatments on correlations was small for EuVar21, EuVar22 and SwiVar21. These results support the findings of Pask et al. [8], Rebetzke et al. [3] and Roche et al. [9], that CT and yield are especially correlated when conditions are not water limited.

Multiple sources of phenotypic variability, genetic or related to treatment, are associated with CT, and this must be taken into account in the analysis [3,18]. It was demonstrated how correlations can be driven or masked by the treatment effect. For example, yield was only correlated with CT before treatment deflation in SwiVar22 but the genotypic correlation between plant height and CT only became evident after treatment deflation. The correlation of CT with plant height and FCC was consistently stronger than the correlation with yield, except for EuVar21. This might indicate that a plant height effect was masking the yield effect on CT in many cases. It remains unclear why plant height and CT showed a weaker correlation in EuVar21 but the FCC measurement of 2022 were consistently correlated with plant height. FCC was not estimated in 2021 but canopies were observed to be very dense in this season. This might have led to saturated FCC with values near 1 (*i.e.* 100 % canopy cover), which might have reduced the effect of plant height on CT, unmasking the correlation between CT and yield.

Although FCC and CT were correlated, the treatment effect of FCC in SwiVar22 was not as evident as for CT (cf. Fig. S24g and Fig. S15). This was possibly caused by saturation of the FCC where the canopy appears largely closed even on the unfertilized part, with an FCC near 1, but is still less dense than the canopy of the fertilized part. Through the less dense canopy, the soil background could have a larger impact on CT [2,8,65], making the nadir-oriented measurements appear hotter compared to the more oblique measurements [15]. The interactions of CT and soil-background can change with increasing temperatures throughout the day. The soil may be cooler than the plant in the morning and warmer later in the day [6].

For the second EuVar21 flight date on 2021-07-01, senescence had progressed for some genotypes while it was still in early stages for other genotypes (Fig. 6e). The strong correlation between senescence ratings and CT underlines the importance of considering phenology in the timing of CT estimates [3,38,66]. However, the 2021 season was characterized by frequent precipitation, and days with optimal conditions for CT estimates (no clouds, little wind) were rare. For logistical reasons, it was therefore not possible to conduct the second measurement day earlier and with a less pronounced senescence. Such meteorological and logistical constraints avoiding optimal measurement timing are a common problem in agricultural research, breeding, and variety testing. However, CT measurements during intermediate leaf senescence stages also showed similar correlation patterns, notably with yield, and with measurements taken earlier in the season [29]. Although measurements taken at the same phenological stage are optimal, this is indicating that conclusions drawn from CT show a certain robustness, even when the sample population shows some phenological heterogeneity, *e.g.* in cases where measurement before onset of senescence is not possible.

The correlation with yield was always stronger for CT than for the multispectral indices (DVI, EVI, SAVI). Now, the indices were chosen as approximate measurements of above-ground biomass and LAI and not yield. In addition, correlation with NDVI was not shown, yet NDVI was closely associated with the indices used (Figs. S21–S24). Nevertheless, this underscores the potential of airborne CT for yield prediction in remote sensing, also for temperate climates.

### 4.5 Estimating geometric effects by PLSR modeling

Geometric effects on CT within one image were cited to be as high as 3.5 ​°C [15] and the range of residual values with geometric patterns were larger than 4 ​°C in this study (Fig. 8). The geometric patterns of the residuals were in some cases point-symmetric (*e.g.* Fig. 8d–f) and sometimes looked similar to those of vignetting (Fig. S3) and path-length dependent atmospheric effects (Fig. 8h) or FCC (Fig. 10d). These three effects were very similar in shape and they are all possible causes for these patterns, however, they cannot be disentangled further with this method. The causes and effects of vignetting are well presented in literature (*e.g.,* [[20], [24], [30]]). Atmospheric effects might be negligible when flown at low altitudes [12,67], however, at higher altitudes they might become important [26], as shown in Fig. 8g & h. This study assumed an oversimplified length-dependent model. For higher altitudes, the attenuation could be estimated based on MODTRAN radiative transfer models [26,[68], [69], [70]]. In addition to flight height, the strength of the atmospheric effect on the measured temperature depends primarily on atmospheric pressure, air temperature, and humidity [61,71]. FCC residuals showed a similar spatial pattern as CT residuals after processing with a mixed model (Eq. (1)) and it is likely that FCC also contributed to CT variance, where CT associated with a lower FCC appeared higher. FCC therefore affected the genotypic variability of CT as was shown with correlation between CT and FCC, but also the residual FCC pattern. Geometric effects on CT can be expected to be more pronounced for canopies with lower FCC, as their apparent FCC changes from low to almost closed canopy for oblique viewing geometries. In contrast, for almost closed canopies with an almost saturated FCC towards 1, this change is very limited (Fig. 10c). Like plant height, FCC is a structural trait of the wheat canopy, and structural traits interact with CT. Other structural traits not considered in this study but with a potential impact on CT include LAI or leaf angle [2,6,13,18].

Often, the residuals also contained more axisymmetric and continuous trends. Such trends could be caused by BRDF or unilaterally warmed spikes. However, such trends usually feature a gradient parallel to the principal plane of the sun [15,72]. This was not always the case (see, *e.g.*, Fig. 8a–c). This could possibly be caused by an interaction of sowing row direction and incident sunlight, where the spacing between the sowing rows allows light to penetrate the canopy and warm the plant from one side, but not from the other (*e.g.,* [73]). Another possible explanation is the camera orientation not being perfectly nadir. With a slightly tilted camera, some geometric effects would still be concentric with the image center (*e.g.* vignetting), while other effects like FCC would not align with the center of the image anymore. The concentric and eccentric patterns would then combine into a less point-symmetric pattern with a more continuous appearance.

In PLSR modeling, covariates without absolute value transformation are better suited to describe continuous effects, while covariates after absolute value transformation rather correspond to point-symmetric effects. Based on PLSR coefficient magnitudes, continuous effects (initial covariates without absolute value transformation) were generally more important in explaining residual variance than point-symmetric effects (absolute covariate values) and PLSR coefficient magnitudes for point-symmetric effects were close to zero for most cases (Fig. 8i).

PLSR modeling allowed the explanation of a significant proportion of residual variance (Table 4) as geometric effects. It should be noted that the proportion of variance that can be explained by PLSR also depends on the magnitude of the initial variance. However, in this study no clear correlation between initial variance and variance explained by PLSR could be shown. Yet, it is hypothesized that the relatively large proportion of residual variance explained by PLSR in SwiVar2021 was due to the relatively low overall variance in this trial, which increased the proportion of residual variance in overall variance. The proportion of explained variance was consistently greater on data without vignetting correction, indicating that vignetting correction and PLSR were reducing initial variance of the same geometric dimensions, *i.e.* PLSR was also modeling vignetting. The proportion of variance that was accounted for by vignetting correction could therefore not be explained by PLSR, which was decreasing the proportion of residual variance explainable by PLSR.

However, the contribution of residual variance to total variance was relatively small (Fig. 8j). Especially point-symmetric effects such as vignetting and FCC seemed to have little impact on total variance, as demonstrated by the low importance of absolute coefficient values in PLSR modeling. The relatively small impact of vignetting correction was also supported by the low difference of the proportion of residual variance explained by PLSR between the data with and without vignetting correction. This difference in explainable residual variance was only 10.9 ​% and it is hypothesized that this percentage is also an approximation of the total importance of vignetting correction. Furthermore, vignetting correction had little impact on total variance (Fig. 4a) but also on the correlation of CT with other phenotypic traits (Fig. 4b–d). The contribution of residual variance to total variance might vary depending on the cropping system under observation. A row crop with a larger inter-row spacing or a poor plant development associated with a lower FCC might feature more pronounced FCC patterns and therefore stronger geometric trends of CT. Kelly et al. [20] and Perich et al. [15] report that such geometric effects are more important when analyzing CT based on single images. When CT analysis uses multi-view or orthomosaics, plot estimates are based on multiple images or selected for most nadir-oriented views, both reducing the geometric impact on plot-wise estimates.

### 4.6 Unexplained residual variance of CT

The sequential application of mixed models and PLSR models could explain a large proportion of variance. But there will always remain unexplained residual variance and though the contribution of residual variance to total variance might be negligible, some possible causes of residual variance are mentioned in the following. Residual variance could be caused by non-geometric non-uniformity effects that neither the vignetting correction nor the PLSR could account for. Also, non-continuous effects impacting CT, like temporal CT inconsistencies due to gusts, might not be accounted for as well as the sensor noise beyond thermal drift, *i.e.*, dark signal noise Aasen et al. [24]. The canopy may also feature holes, caused, for example, by heterogeneous emergence, damage from rodents, or previous sampling events, which could have different impacts on CT estimates depending on viewing geometry [6].

### 4.7 Emissivity and CT variance

An important determinant of CT variance that is often ignored in airborne thermography of crops is emissivity. Emissivity compares the TIR radiation emitted by a surface with the TIR radiation emitted by a black body at the same temperature [12,74,75]. Two objects of different materials can have the same temperature, but if they have different emissivities, they appear to have different temperatures in thermal images. Messina et al. [12] summarizes multiple factors that influence emissivity: color, chemical composition, surface roughness, moisture content, field of view, viewing angle, spectral wavelength, etc. [[75], [76], [77]]. The emissivities cited in the literature vary, but in general, for healthy leafy vegetation, an emissivity of 0.99 can be assumed [52], where for dry vegetation, emissivity from 0.88 to 0.94 were reported. Water has an emissivity of 0.99 and dry soil an emissivity of around 0.92 [61,75,78]. Stressed vegetation generally has a lower emissivity than healthy vegetation, and plant emissivity is highly sensitive to water content [17]. Diaz et al. [52] assumed an emissivity of 0.99 when the NDVI of the respective pixel was above 0.5. NDVI was below 0.5 for some measurements at the last measurement date of EuVar21 (Fig. S21c) and SwiVar21 (Fig. S23c). Therefore, different emissivities would have to be assumed for different plots of the same measurement flight. This would come with the necessity of estimating the correct emissivity for the specific plot, which can lead to large differences in CT estimates. For example, when an object has a temperature of 20 ​°C, under the assumption of an emissivity of 0.99, it would appear to be 293.15 ​K∗0.99 ​= ​290.22 ​K or 17.07 ​°C. Assuming an emissivity of 0.98, the apparent temperature would be 293.15 ​K∗0.98 ​= ​287.29 ​K or 14.14 ​°C. The difference between the two emissivity assumptions of only 0.01 corresponds to 2.93 ​°C, which is about the range of genotype-specific differences in the experiments of this study and is therefore far too large to study genotype-specific differences of CT.

To avoid the introduction of errors by estimating erroneous emissivity values for individual plots, it might thus be more appropriate to assume a constant emissivity for all measurements, when no absolute CT values are needed. The absolute value of CT is particularly important for physiological investigations, where absolute values are needed to approximate physiological quantities such as transpiration rate or gas exchange. If, on the other hand, relative CT is compared, the absolute value plays a lesser role. For this study, for example, an emissivity of 1 was assumed. A stressed vegetation, in most situations, would have a higher CT and at the same time a lower emissivity. The effect of assuming a too high emissivity would thus lead to a too low estimate of temperature on the thermal image, and the question remains whether differences of apparent CT on thermal images arise from differences in CT or from a varying emissivity.

In addition, emissivity might also be affected by FCC and LAI. The emissivity of soil can be significantly lower than the emissivity of healthy vegetation, and low FCC, or low LAI, even at a relatively high FCC, might impact the emissivity of a plot, biasing the CT estimates. Cheng et al. [79] demonstrated for satellite data that the error of emissivity estimates is lower when the emissivity of the soil background is closer to the emissivity of the vegetation, and when the LAI of the vegetation is higher. Sorbino et al. [80] explored the dependence between emissivity and viewing angle and described that the level of the angular dependency is related to LAI.

However, measuring emissivity in the field is a very tedious task that cannot be easily implemented [81]. It must be measured at night [82], or by shielding the vegetation with boxes to exclude environmental radiation from the surroundings [83]. Thus, in many field studies, the emissivity is ignored [1,2,6,15] while other assume a fixed emissivity (often 1), as in this study [81,84,85].

For satellite-based estimates of LST, model-based approaches to determine emissivity were proposed [79,80,86], *e.g.* based on NDVI estimates. To the best of the authors knowledge, there are no similar studies for drone-based CT estimates. Yet, the study of Treier et al. [29] provides the tool to estimate CT in dependence of viewing geometry. In addition, Roth et al. [35] used the multi-view approach to determine the LAI of soybean. These two approaches could be combined with emissivity estimates to promote a more robust understanding of the interaction of CT, emissivity, viewing geometry, and LAI.

## 5 Conclusions

Canopy temperature is affected by manifold sources of variance which interact with each other. Multiple sources of variances were reviewed based on extensive field data and by using the previously suggested multi-view approach in this study. Experimental sources of variance (genotypes and treatments) were impacted by meteorological conditions in the growing season. To reveal the relation between CT and other traits, corrections for confounding sources of variance (*e.g.* thermal drift, spatial trends, geometric effects) were applied. Temporal trends were consistently the most important confounding source of variance, followed by spatial trends. Estimation of spatial trends and their disentanglement from temporal trends remain a challenge, but a path to an improved estimation of the spatial trends by flying multiple times with different flight paths was proposed. Phenotypic relationships can be masked or result from artifacts of random but concurrent instantaneous trends. After correction for disturbing trends, the correlation between phenotypic traits was accentuated. Not applying such corrections might thus entail misleading conclusions on phenotypic relationships with CT. Plant height and FCC were shown to be important phenotypic drivers of CT in many situations and were more correlated with CT than yield, except for well-watered conditions and a diverse set of genotypes. However, CT was constantly more correlated with yield than multispectral proxy measurements of above-ground biomass and LAI. Although other CIs may be better suited to estimate yield, this highlights the potential of CT to enhance in-season yield estimates in temperate climates, for example, to avoid losing all the information of an experiment due to a hail storm close to harvest. Flag leaf rolling had a relatively small but significant impact on CT. Complex interactions of above-ground biomass, flag leaf rolling as drought symptom, water use by the canopy, and CT were demonstrated. Treatment effects can be considerable and modify other phenotypic traits and their interaction with CT. Geometric trends were shown to have distinct patterns for flights and campaigns, but they explained a relatively low proportion of total variance. Temporal, spatial, genotypic, treatment related and geometric effects together explained the largest part of the initial variance, leaving just a small proportion unexplained. It is hypothesized that many insights on the sources of variance of uncalibrated airborne thermography that were gained in this study are transferable to other crops and other climatic conditions (especially hotter). In cooler conditions, the correlation between CT and yield might be limited due to lower transpirational demands of the plants, leading to lower genotype specific differences of CT. As the study was conducted with wheat, a row crop with relatively large inter-row spaces, following the rationales outlined in this study should also lead to meaningful results in the analysis of other crops with low FCC. At the same time the rather ephemeral character of CT and its strong interaction with the environment should always be kept in mind, as they entail a limited transferability of CT information between different environments. Nevertheless, within the different environments in this study, multi-view thermography served as a means to foster a comprehensive and empirically backed understanding of variance components in drone-based CT estimates. This facilitates the planning, conduct, and interpretation of drone-based CT screenings in variety testing and breeding.

## Authors’ contribution

Simon Treier: conceptualization, methodology, software, formal analysis, visualization, writing – original draft. Lukas Roth: conceptualization, supervision, methodology, review & editing. Juan M. Herrera: project administration, funding acquisition, conceptualization, supervision, methodology, acquisition, writing – review & editing. Achim Walter, Nicolas Vuille-dit-Bille, Lilia Levy Häner, Helge Aasen, Andreas Hund,: writing – review & editing.

## Funding

This study was financed by 10.13039/501100022575Agroscope and the work of Simon Treier was in part supported by the two H2020 projects InnoVar and Invite.

## Declaration of competing interest

The authors declare that they have no known competing financial interests or personal relationships that could have appeared to influence the work reported in this paper.
