# Supplementary material for: Analysis of variance and its sources in UAV-based multi-view thermal imaging of wheat plots
Source: Plant Phenomics. 2025 Apr 30;7(2):100046. doi: 10.1016/j.plaphe.2025.100046 (PMC12709991; doi:10.1016/j.plaphe.2025.100046)
Supplement: Multimedia component 1 [file mmc1.pdf]

Analysis of variance and its sources in UAV-based multi-view thermal imaging of wheat plots - Supplementary Materials

S1 Experimental design - EuVar

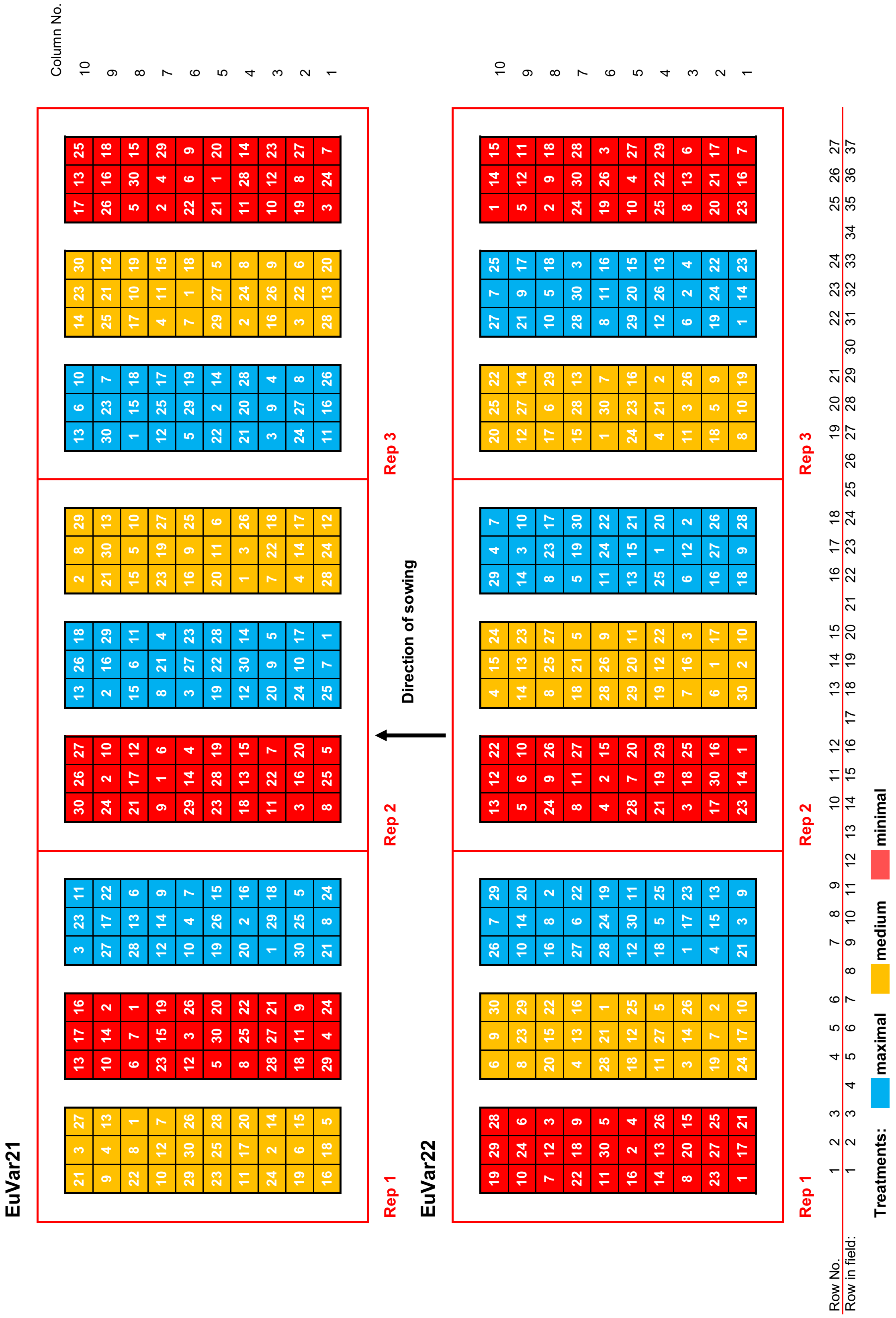

Figure S1: The experimental design of EuVar for the two years. The numbers inside the blocks indicate the genotypes.

S2 Experimental design - SwiVar

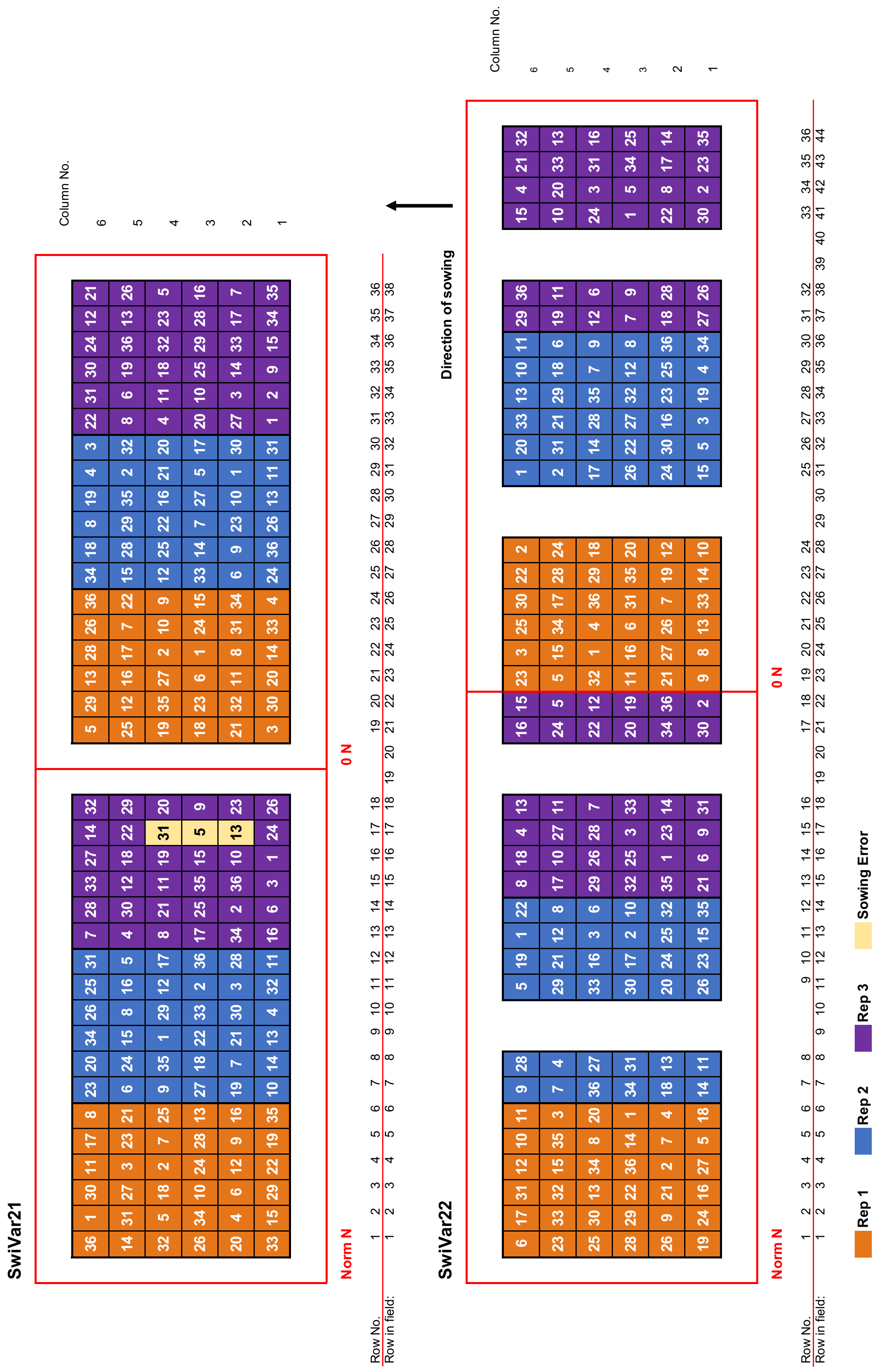

Figure S2: The experimental design of SwiVar for the two years. The numbers inside the blocks indicate the genotypes.

S3 Details on Field treatments

Table S1: Overview of trial treatments and most important field interventions for all trials. "too wet" indicates that treatments were intended but could no be applied as conditions were too wet and heavy machinery could not enter the field.

				Herbicides					Fertilization (kg/ha)			
**Experiment**	**Treatment**	**Sowing date**	**Harvest date**	Monocot. 1st	Monocot. 2nd	Dicot.	**Growth regulator**	**Fungicide**	N	CaO	MgO	SO_3
	Minimal						-	-				
**EuVar21**	Medium	2020-10-22	2021-07-20	Archipel®	too wet	too wet	Moddus®	-	140	15	32	30
	Maximal						Moddus®	Amistar®				
**SwiVar21**	Fertilized	2020-11-07	2021-07-20	Archipel®	too wet	too wet	-	-	140	15	32	30
	Not fertilized						-	-	-	-	23	30
	Minimal						-	-				
**EuVar22**	Medium	2021-10-15	2022-06-30	Archipel®	Othello Star®	Cleave®/ Express Max®	Moddus®	-	140	23	37	30
	Maximal						Moddus®	Amistar®				
**SwiVar22**	Fertilized	2021-10-15	2022-07-06	Archipel®	Othello Star®	Cleave®/ Express Max®	-	-	140	5	26	30
	Not fertilized						-	-	-	-	23	30

Table S2: Details on fertilizer application with split N applications.

				Fertilization (kg/ha)			
	**Treatment**	**N Application split**	**Date**	N	CaO	MgO	SO_3
**EuVar21**	All	-	2021-02-22		-	23	30
		1	2021-02-23	50	-	-	-
		2	2021-03-24	60	10	6	-
		3	2021-04-20	30	5	3	-
**SwiVar21**	Fertilized	-	2021-02-22	-	-	23	30
		1	2021-02-23	50	-	-	-
		2	2021-03-24	60	-	-	-
		3	2021-04-20	30	-	-	-
	Not fertilized	-	2021-02-22	-	-	23	30
**EuVar22**	All	1	2022-02-08	50	8	5	-
		-	2022-03-03	-	-	23	30
		2	2022-02-23	60	10	6	-
		3	2022-04-28	30	5	3	-
**SwiVar22**	Fertilized	-	2022-03-03	-	-	23	30
		1	2022-03-11	50	-	-	-
		2	2022-03-30	60	-	-	-
		3	2022-04-28	30	-	-	-
	Not fertilized	-	2022-03-03	-	-	23	30

Table S3: Chemical compositions of field treatments and quantities applied.

**Procuct**	**Active ingredients**	**Application rate (g/ha)**	**Producer**
Archipel®	Iodosulfuron-methyl-sodium	9	Syngenta
	Mesosulfuron-methyl	9	
	Mefenpyr-diethyl	27	
Moddus®	Trinexapac-ethyl	125	Syngenta
Amistar®	Azoxystrobin	200	Syngenta
	Cyproconazole	80	
Othello Star®	Iodosulfuron-methyl-sodium	9	Bayer
	Mesosulfuron-methyl	9	
	Mefenpyr-diethyl	27	
	Thiencarbazone-methyl	7.5	
Cleave®	Fluroxypyr	90	Syngenta
	Fluroxypyr-meptyl	130	
	Florasulam	23	
Express Max®	Metsulfuron-methyl	5	Syngenta
	Tribenuron-methyl	5	

S4 Overview on flights

Table S4: Overview on number of thermal measurements per date and project

Year	Project	Date	No. of flights
2021	EuVar	2021-06-12	13
	SwiVar	2021-06-19	15
	SwiVar	2021-06-28	13
	EuVar	2021-07-01	9
2022	EuVar<br>SwiVar	2022-05-14	3<br>6
	EuVar<br>SwiVar	2022-05-18	3<br>6
	EuVar<br>SwiVar	2022-06-04	6<br>3
	EuVar<br>SwiVar	2022-06-11	5<br>3
	SwiVar	2022-06-14	8
	SwiVar	2022-06-18	6

S5 Flight campaigns

Optimal conditions for TIR imaging surveys are a clear blue sky, warm temperatures, and little or no wind (Perich et al., 2020). In 2021, the weather conditions were often suboptimal for flying (rain and cloud cover throughout the growing season, Fig. S5a). On rare days with suitable weather, as many flights as possible were conducted on one experiment before, during, and after solar noon.

2022 was a very hot and dry year (Fig. S5b). The phenological window suitable for flying was short, but within this window conditions were often suitable for TIR imaging. Consequently, in season 2022, flights were conducted on more days but on individual days, with fewer flights per day than in 2021. Flights were carried out between late morning and mid-afternoon as suggested by Deery et al. (2016), and Perich et al. (2020), with only some exceptions, where flights were also taken later in the day. Based on the results of 2021, flights were restricted to times after 12:00 in 2022. Fig. S5 provides an overview on when TIR measurements were conducted within the growing season, while Figs. S6 and S7 show, at what time of day, the flights were conducted during the single days. In total, 99 flights were performed (Table S4).

S6 Camera settings and flight planning

The flights were carried out with a DJI Matrice 200 drone (SZ DJI Technology Co. Ltd., China) that carried a DJI Zenmuse XT TIR sensor equipped with a 9 mm, f/1.4 lens. Pixel resolution of 640 x 512 was achieved by individual uncooled VOx microbolometers arranged in a focal plane array. The field of view (FOV) was 69° x 56°. The temperature was measured in the wavelength range of $7.5-13.5\,\mu m$ and the thermal sensitivity was $< 50\,mK$. In high gain mode, the camera could measure the temperature in the range of -25 °C to +135 °C. The absolute measurement accuracy was ± 10 °C according to the manufacturer. The external parameters were set in the DJI Pilot software (SZ DJI Technology Co. Ltd., China) to the same values for all flights using the default DJI settings. The scene emissivity was set to 100 %, background and air temperature were set to 22 °C. The sensor

provided the option of periodical flat-field correction throughout measurement to reduce the noise of non-uniformity effects. This periodic compensation would reduce the comprehensibility of drift effects. In addition, Kelly et al. (2019) showed that the non-uniformity correction alone was not sufficient to correct for the drift effect during flights. The flat field correction was therefore deactivated following Mesas-Carrascosa et al. (2018).

The flights were planned with DJI Pilot software. The exposure interval was 2 s. While mission planing often is done by defining a minimal front- and side overlap, this was not possible due to software restrictions for the drone−sensor combination used. In addition, using way-point flight planning with a fixed exposure interval allowed for a manually defined camera heading throughout the flight. Therefore, the heading of drone and TIR camera remained relatively stable throughout the flight and did not change with flight path direction changes. The flight speed was limited to $4\,\mathrm{m\,s^{-1}}$.

S7 DEM creation

TIR images often do not provide enough spatial detail to generate DEMs of sufficient quality (*e.g.* Malbéteau et al., 2021; Treier et al., 2024). Thermal images have lower pixel resolution and contrast compared to RGB images (Boesch, 2017). TIR based DEMs may therefore appear flat with no distinct plot pattern. Thus, DEMs were also based on the RGB data of Micasense RedEdge-MX Dual camera (MicaSense Inc., Seattle, Washington, USA) which allows for more spatial detail.

DEMs were created on the basis of aligned images in Agisoft Metashape and were derived from thermal data in 2021, but not in 2022, when DEMs were generated from RGB data. Both methods allowed generating DEMs of sufficient positioning precision (positioning RMSE vertical: 2.5 cm, horizontal: 1.5 cm based on Agisoft alignment error estimates for ground control points). For each year, a representative DEM was chosen that was created from images taken after the wheat stem elongation phase and before early senescence, when the canopy height remained stable. The quality of the DEMs was checked by visually inspecting the plausibility of the positioning of the masks projected on single images in multi-view pre-processing. The projected masks needed to be centered within plots and rectangular in shape. For EuVar21, the DEM was based on the second flight of the thermal campaign flown on 2021-06-12 at 12:30 and for SwiVar21, the DEM was based on the third flight of the thermal campaign flown on 2021-06-19 at 16:30 with a flight height of 40 m for both flights. The ground sampling distance (GSD) of the TIR images was 5.15 cm/pix and the spatial resolution of the DEMs was 41 cm/pix and 16 cm/pix for EuVar21 and SwiVar21 respectively. With this coarse resolution, inconsistencies such as holes in the DEM could be leveled out. The DEMs used in 2022 were based on flights with the Micasense sensor at a flight height of 40 m at 2022-06-04 and 2022-05-18 for EuVar22 and SwiVar22 respectively. The GSD of the images was 2.71 cm/pix. The DEMs of 2022 did not exhibit holes, and the spatial resolution of the DEM was set to 2.71 cm/pix too.

S8 TIR image pre-processing

Radiometric JPEG format contains an 8-bit gray scale JPEG image as well as a 14-bit array with digital numbers (DN), which represent the magnitude of TIR radiation (Kelly et al., 2019). The DNs in the 14-bit arrays of the radiometric JPEGs were transformed to TIFF files representing temperature in $^{\circ}C$ x 1000 by using a Python 3.8 script (van Rossum, Guido and Drake, Fred L., 2009) and a modified version of the Flir Image Extractor (`https://github.com/ITVRoC/FlirImageExtractor`), which allowed for batched processing.

Plot masks were created for each plot in Qgis 3.16 (QGIS Development Team, 2022), to determine the ROIs from which data was used for analysis. To account for border effects in the field and for inaccuracies of georeferencing and superimposition of different flights, a border buffer of 25 cm was applied to all masks on plot width. On plot length, the buffer was up to 1 m, leaving at least a surface of $2.1\,\mathrm{m}^2$ to be analyzed in each plot. The plot masks were saved to GeoJSON format.

Imaging techniques deliver pixel values in a 2-D space. In order to evaluate experimental units, pixels within ROIs in this 2-D space must be analyzed. Usually, this is done using zonal statistics,

that is, the pixels within ROIs are reduced to single values using statistical aggregation functions. In this work, an empirically determined specific percentile for each year was used.

The procedure for finding an optimal percentile was described in Treier et al. (2024). In short, for each percentile, heritabilities were calculated in a simplified mixed model in SpATS (Rodríguez-Álvarez et al., 2018). The resulting percentile-heritability relations were plotted for graphical comparison. Two quantitative criteria were used to select the percentiles: Select a percentile in the center of a percentile region where (1) the heritability is close to the maximum, and (2) closely adjacent percentiles have similar heritabilities, *i.e.* the heritability is stable in the respective percentile region. For each experiment in each year, the optimal percentile was determined. The values within the ROIs were reduced to a single value by using the optimal percentile. One value per measurement (for multiple measurements per plot) was then used as plot-wise CT value in further analysis. The same percentile was used for the aggregation of all flights on one experiment within one year.

S9 Multi-view pre-processing

The camera positions (longitude, latitude, height) and orientations (pitch, roll, yaw) at the moment of triggering of individual images were estimated in an indirect sensor orientation approach (Benassi et al., 2017) in Agisoft Metashape after aligning images. Using the estimated trigger positions, the single images were projected onto the DEMs by ray tracing as described in Roth, Aasen, et al. (2018), Roth, Camenzind, et al. (2020) and Treier et al. (2024). This allowed for the projection from geographic coordinates (*e.g.* EPSG:2056 reference system) to image coordinates. As a result, plot masks of ROIs were created for each trigger position (*i.e.* for each image) where at least one plot was entirely inside the field of view (FOV) of the camera. As coordinates were identical for 8-bit JPEG images and 14-bit intensity value arrays, the image-wise masks could be directly applied to the temperature TIFF files. This approach of identifying the ROIs for each plot on every single image is referred to as multi-view. For each plot on each TIF file, all percentiles were extracted with a Python 3.8 script and saved to a CSV file.

S10 Flight operation

In 2021, the camera was turned on at least 15 min before each flight to allow the temperature signal to stabilize. In 2022, an additional set of batteries was used and the stabilization period was increased to 30 min. In situations where the battery was not sufficient anymore to complete all flights, the temperature stabilization was not repeated after a rapid battery change. After the first flight campaigns in 2021, a rather strong drift of apparent temperature was noticed that seemed to be particularly strong during the beginning of flights. To further reduce initial drift, the drone was hovered above the wheat field for about one minute in addition to the previous temperature stabilization on the ground before the measurement flight sequence was started.

S11 Thermal ground control points

GCPs were produced following Perich et al. (2020) by gluing triangles of 2 mm thick aluminum sheets on polystyrene foam plates. These plates had an extent of 1 m x 0.5 m or 0.5 m x 0.5 m. Unlike in Perich et al. (2020), the aluminum sheets were left blank as was done in other TIR surveys (*e.g.* Mesas-Carrascosa et al., 2018; Aragon et al., 2020) and not painted black. This avoided large temperature gradients in the FOV and reduced possible adjacency effects of hot objects (Aragon et al., 2020; Zheng et al., 2019).

S12 Georeferencing images

The 8-bit JPEGs of the radiometric image as well as the RGB images were aligned in the structure-from-motion-based software Agisoft Metashape Professional (Agisoft LLC, St. Petersburg, Russia).

TIR images feature a low spatial resolution and are therefore difficult to georeference. No precise GPS device was available to measure GCP positions, and an indirect referencing approach was used. One RGB project served as a reference project and was referenced by the positioning information of the drone available for each image in the meta-data. The GCP coordinates were extracted from this project and used to reference all other projects of one year. Conventional GCPs are difficult to detect in TIR images, and in one RGB project, the RGB GCPs were visible together with the thermal GCPs. The locations of the thermal GCPs were then extracted from this RGB project and used to reference the thermal projects. This allowed for a correct geographic orientation and a absolute positioning precision within 2 m horizontally and vertically according to a quality check in Qgis (QGIS Development Team, 2022). With this procedure all TIR flights were georeferenced in the Cartesian Swiss coordinate system EPSG:2056 (CH1903 + LV95), which allowed one to precisely superimpose the aligned images of the different flights. The relative positioning precision between flights was estimated to be 15 cm or smaller based on marker position error estimates in Agisoft.

The 8-bit JPEGs were preferred over the 14-bit TIFF images in the process of aligning images, as they provide better contrast and contain meta information on TIR camera position and orientation during triggering, which was allowing for a valid alignment more reliably. However, these 8-bit JPEG are just a nonlinear, visually augmented interpretation of TIR with a value range of 0 to 255 and could not be used for analysis of temperature. As the pixel position remained consistent between the two formats, 8-bit JPEG images were replaced by 14-bit TIFF files after alignment for further temperature analysis.

S13 Covariates related to viewing geometry

Table S5: List of all covariates calculated from multi-view data

**Covariate**	**Description**
Angle Sun-Plot-Drone	The angle between sun, plot and drone
Azimuth drone	The Azimuth of the drone, seen from the plot (horizontal planar clockwise angle from north)
Azimuth sun	The Azimuth of the sun, seen from the plot
Azimuth diff	Difference between the two Azimuth angles of sun and drone
Elevation sun	The vertical angles from the horizon to the sun
Elevation drone	The vertical angles from the horizon to the drone
Lateral angle row dir.	Lateral angle of the plot relative to the drone in sowing row direction
Lateral angle sun dir.	Lateral angle of the plot relative to the drone in sun direction
Longitudinal angle row dir.	Longitudinal angle of the plot relative to the drone in sowing row direction
Longitudinal angle sun dir.	Longitudinal angle of the plot relative to the drone in sun direction
Lateral dist row dir.	Lateral distance of the plot relative to the drone in sowing row direction
Lateral dist sun dir.	Lateral distance of the plot relative to the drone in sun direction (i.e. orthogonal to principal plane of the sun)
Longitudinal dist row dir.	Longitudinal distance of the plot relative to the drone in sowing row direction
Longitudinal dist sun dir.	Longitudinal distance of the plot relative to the drone in sun direction (i.e. in the principal plane of the sun)
Trigger timing	The time stamp when each TIR image was taken
Sensor x	X coordinate of the plot center on the sensor plane (image coordinates)
Sensor y	Y coordinate of the plot center on the sensor plane (image coordinates)
Total dist.	Total distance between drone and plot center

S14 Spectral properties of the Micasense RedEdge-MX Dual Camera System

Table S6: Specification of the ten bands of the Micasense RedEdge-MX Dual Camera System

Micasense band-name	Band variable	Center wave-length (nm)	Band width (nm)	Micasence Band Suffix
Coastal Blue	$Blue_{444}$	444	28	6
Blue	$Blue_{475}$	475	32	1
Green	$Green_{531}$	531	14	7
Green	$Green_{560}$	560	27	2
Red	Red_{650}	650	16	8
Red	Red_{668}	668	14	3
Red Edge	Red_Edge_{705}	705	10	9
Red Edge	Red_Edge_{717}	717	12	5
Red Edge	Red_Edge_{740}	740	18	10
Near IR	NIR_{842}	842	57	4

S15 Multispectral measurements

The sensor was carried by a DJI Inspire 2 drone (SZ DJI Technology Co. Ltd., China). The flight height was 60 meter in 2021 and 40 meter in 2022 resulting in a ground sampling distance (GSD) of 3.98 cm and 2.71 cm, respectively. The side overlap was set to 80 %, the flight speed was limited to 5 m s^{-1} and an image was taken every 2 s, resulting in a front overlap of approximately 70 % and 60 % for the two flight heights, respectively.

S16 Vignetting correction

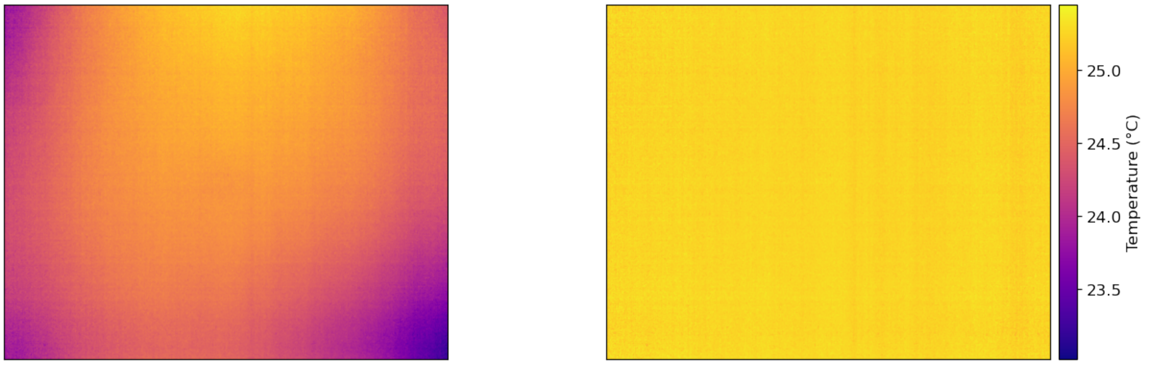

(a) Before correction (b) After correction

Figure S3: (a) shows an image of a homogeneous PVC sheet that was part of the set used to create a vignetting correction image. A vignetting pattern is clearly visible with a cooling trend toward the edges. (b) shows the same image after correction was applied. Vignetting was clearly mitigated and the image of the PVC sheet now appears flat with almost no trends visible.

This procedure was explained in detail in Treier et al. (2024) and for the sake of clarity, the method is described here again. To generate a vignetting correction image, the drone was located indoors with the thermal sensor pointing to a hard foam PVC sheet. The distance between the sensor and the sheet was about 40 cm and the sheet completely covered the FOV of the camera. The ambient temperature was 22 °C and there was no direct light on the PVC sheet. The ambient light in the room was reduced (turned off in the respective section of the room) to mitigate artifacts of light. The PVC sheet was placed inside the room 5 hours prior to use to reach temperature equilibrium.

The camera was started to stabilize. After 1 h, TIR images of the PCV sheet were taken at an interval of 5 s for more than 30 min. A vignetting correction image was then calculated as the

pixel-wise mean of these 413 images in Python 3.8. The pixel valvues of the resulting correction image were subtracted from corresponding pixel values of all TIR images of all flights to obtain vignetting-corrected images. Fig. S3 shows an image that was taken after the camera was running for more than 70 min before and after correction.

S17 Fan experiment to determine the influence of wind

The drone with the thermal camera was placed indoors at an ambient air temperature of 20 °C and the thermal camera pointed to a hard foam PVC sheet (Fig. S4) similar to what was done for the vignetting correction. The distance between the sensor and the sheet was about 120 cm. At a distance of 90 cm and an angle of about 45° a fan was placed, pointing in the direction of the camera. The fan generated a wind speed of about 3 - 3.3 $m\,s^{-1}$ at the sensor. At a distance of 67 cm and an angle of 90°, a Philips Attralux spot (230V, 150W) pointed to the camera as an artificial source of heat. The spot did not point inside the FOV of the camera but just heated it up from the side. The ambient light in the room was reduced to minimize disturbances from other sources of light.

The camera was started for stabilization and images were taken from the beginning. To examine whether sudden and strong temperature gradients have a sustained influence on subsequent TIR readings, warm and hot disturbance objects (hands at body temperature and a water cooker with boiling water) were introduced into the scene for several seconds 35 min after camera startup. Each disturbance was repeated three times with a period of 5 min for stabilization after each disturbance. 75 min after camera startup, the heating lamp was started and after another 5 min, the fans was turned on and off at an interval of 5 min. The heating lamp was turned off again 5 min after the last fan iteration.

On the TIR images, a polygon was defined, covering just the PVC sheet. From within this polygon, the mean temperatures and standard deviation of pixel-wise temperatures were extracted with a Python 3.8 script.

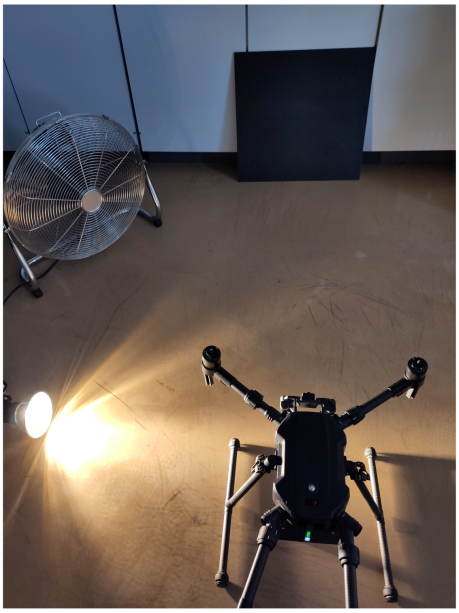

Figure S4: Setup of fan experiment. The drone was pointing at a PVC sheet. From an oblique frontal angle, the fan was blowing in the direction of the sensor. From the side, a lamp was heating the sensor without directly pointing into the FOV of the camera.

S18 Environmental conditions and timing of measurements

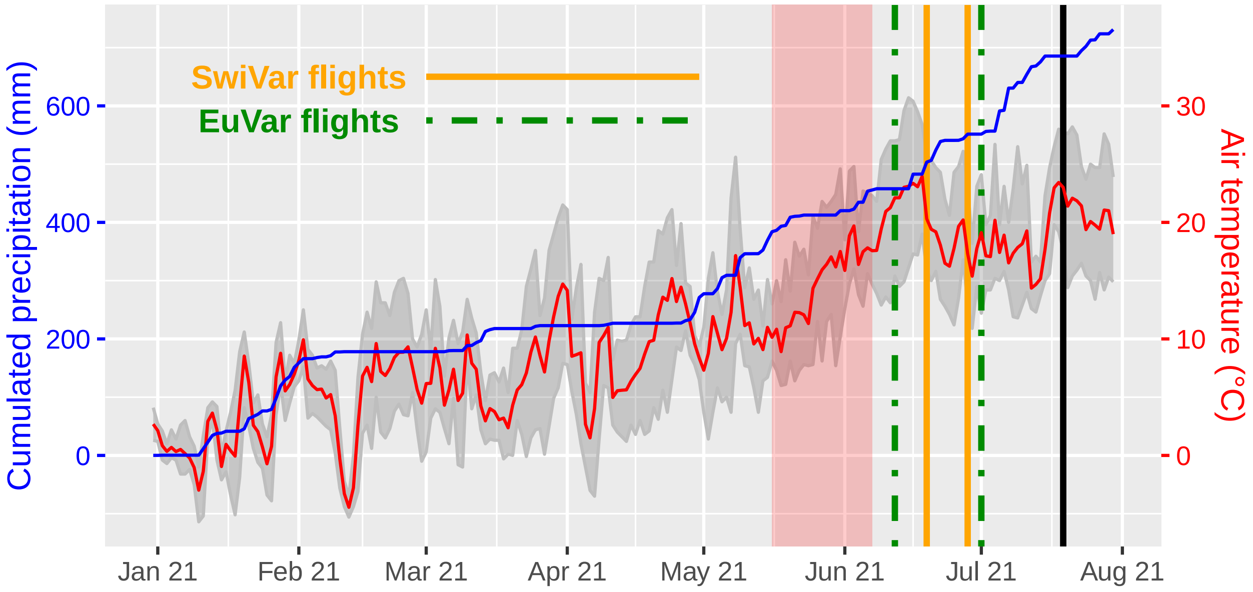

(a) Weather overview of growing seasons 2021

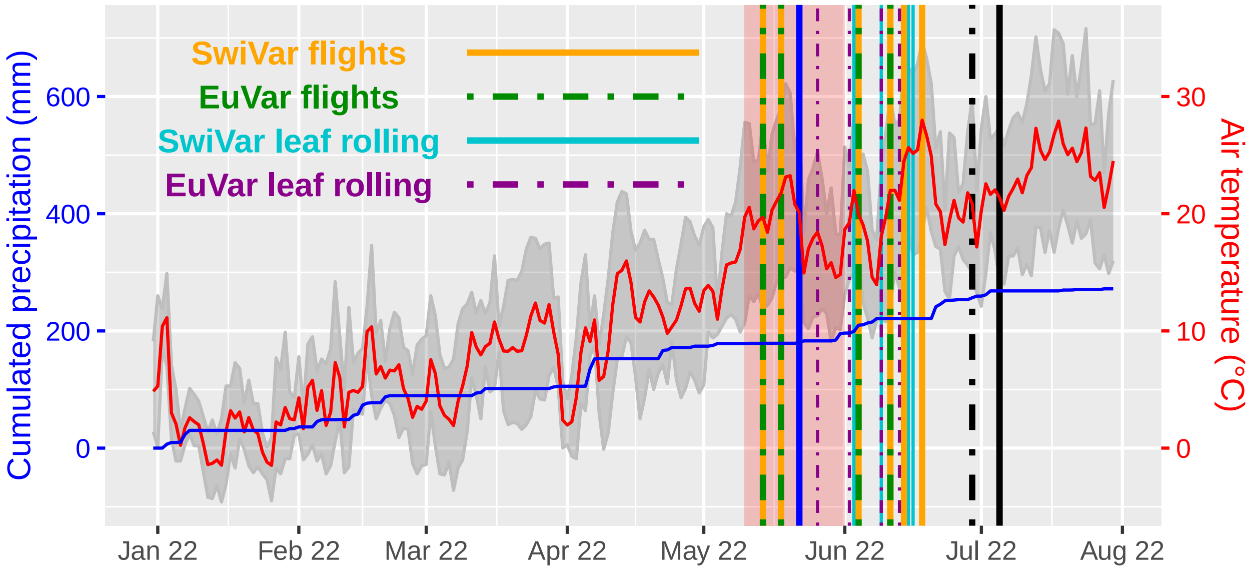

(b) Weather overview of growing seasons 2022

Figure S5: (a) and (b) show the general weather conditions during the growing seasons of 2021 and 2022 from January until after harvest. Red shows the mean air temperature, and the shades indicate daily temperature minima and maxima. The orange lines and green dashed lines represent the flight dates of SwiVar and EuVar, respectively. Cumulative precipitation is shown as a rising blue line, and the vertical blue line indicates an irrigation intervention for SwiVar22 (30 mm of water). During the period shaded in red, heading was observed in the field. Cyan and purple lines indicate flag leaf rolling ratings in 2022. Harvest dates are marked by black lines (the dashed black line in 2022 is the harvest date of EuVar22 which was harvested before SwiVar22).

S19 Weather data

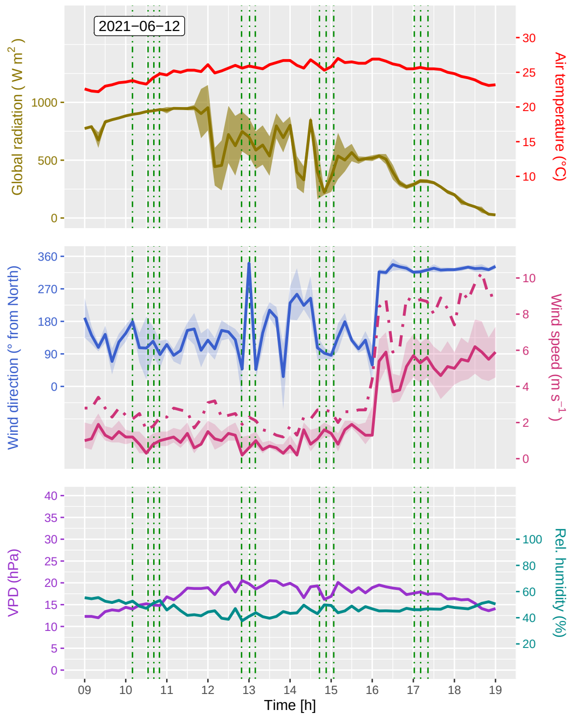

(a) 2021-06-12

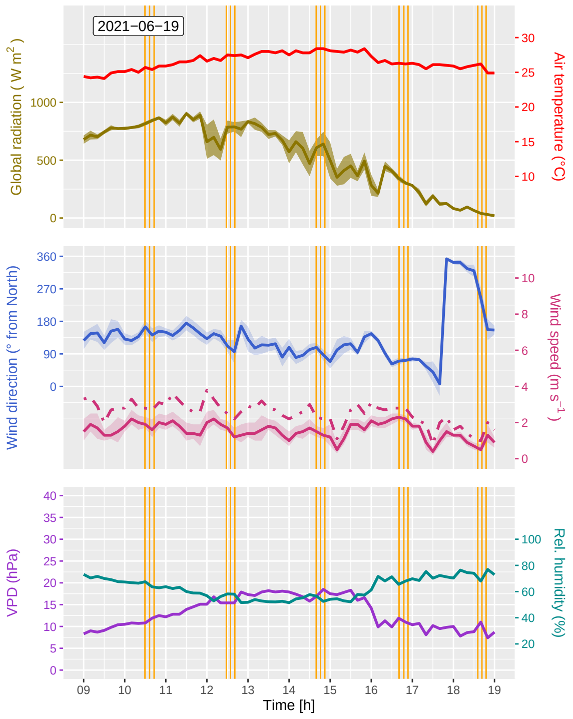

(b) 2021-06-19

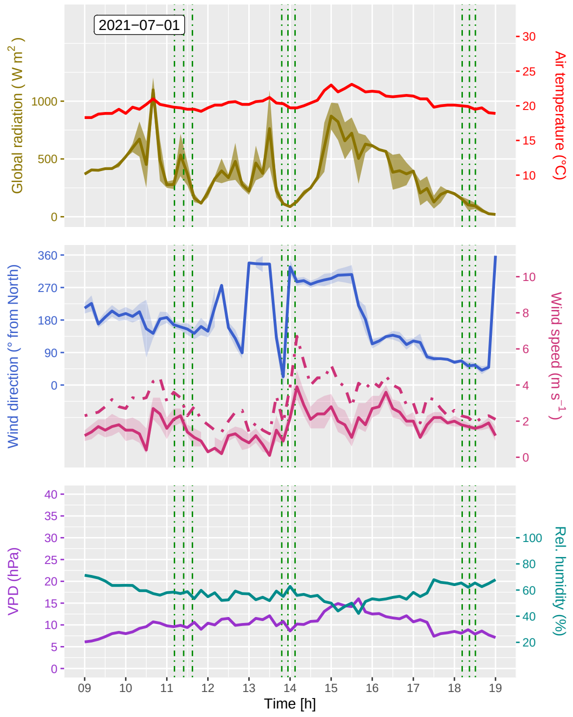

(c) 2021-07-01

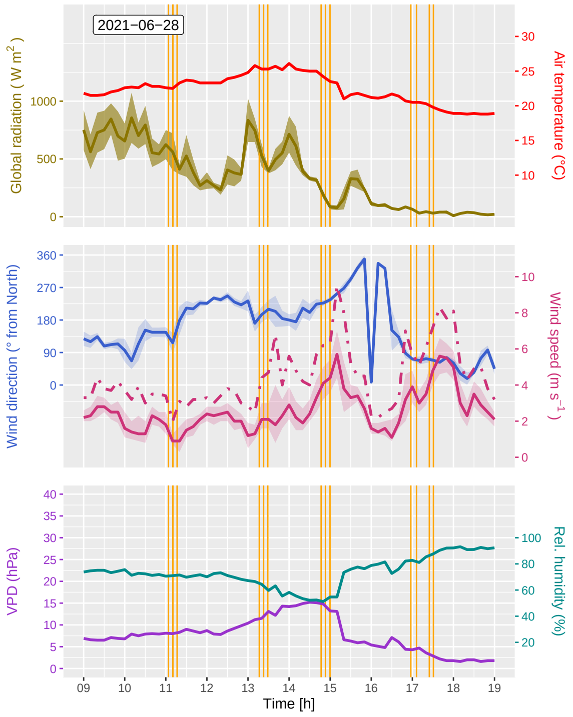

(d) 2021-06-28

Figure S6: (a - c) show the detailed weather conditions on measurement days in 2021. Solid lines show means, shades are means ± SD and dashed lines show the maxima for 10 min intervals. The vertical lines indicate the different flights of EuVar (green) and SwiVar (yellow).

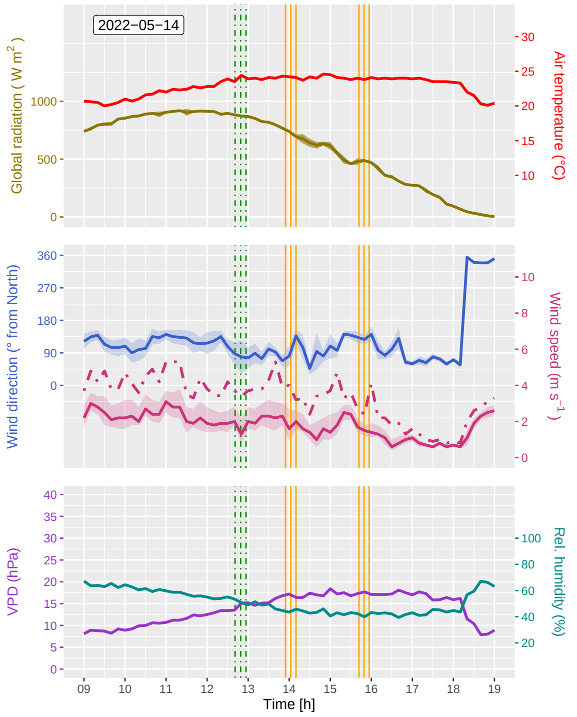

(a) 2022-05-14

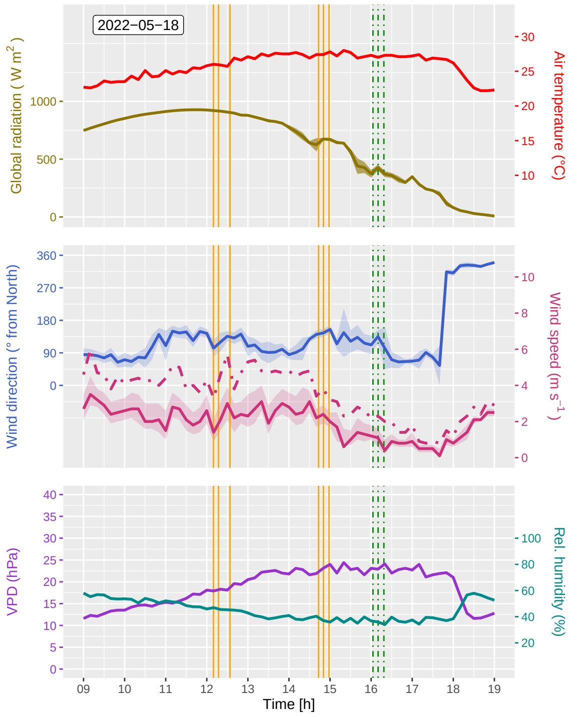

(b) 2022-05-18

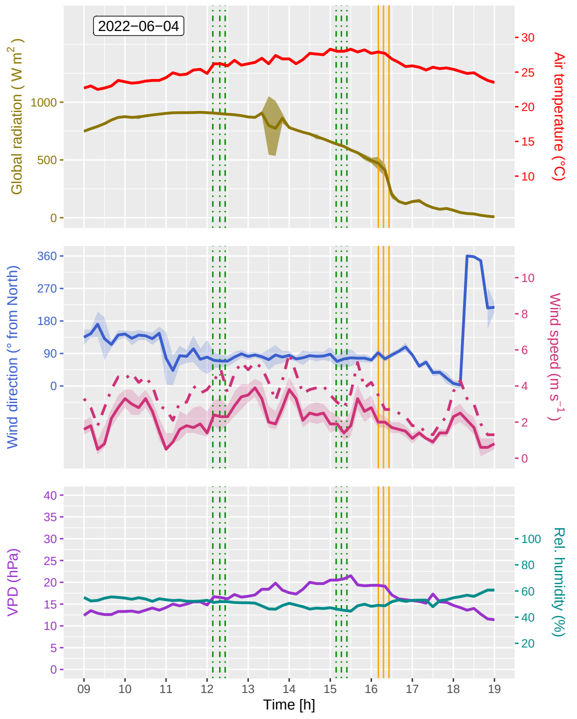

(c) 2022-06-04

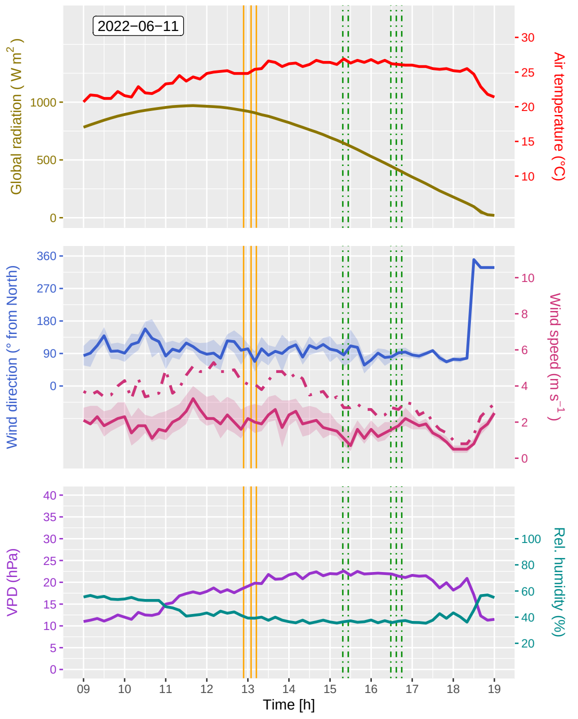

(d) 2022-06-11

Figure S7: (a - f) show the detailed weather conditions on days of measurements in 2022.

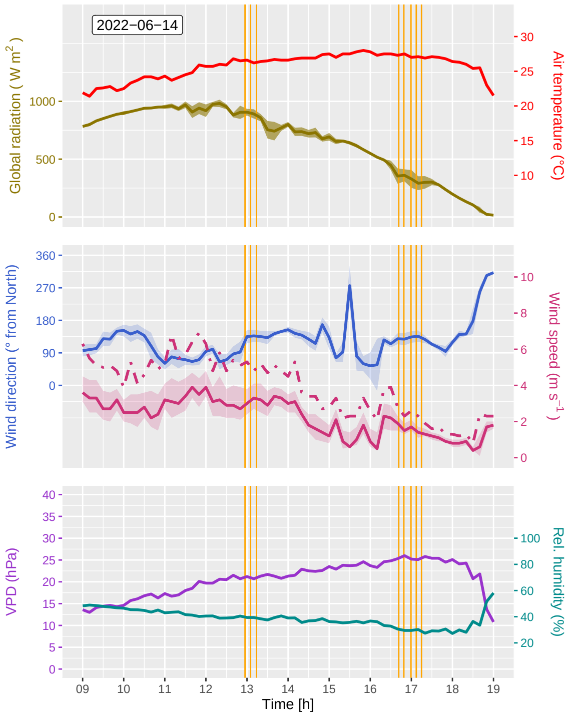

(e) 2022-06-14

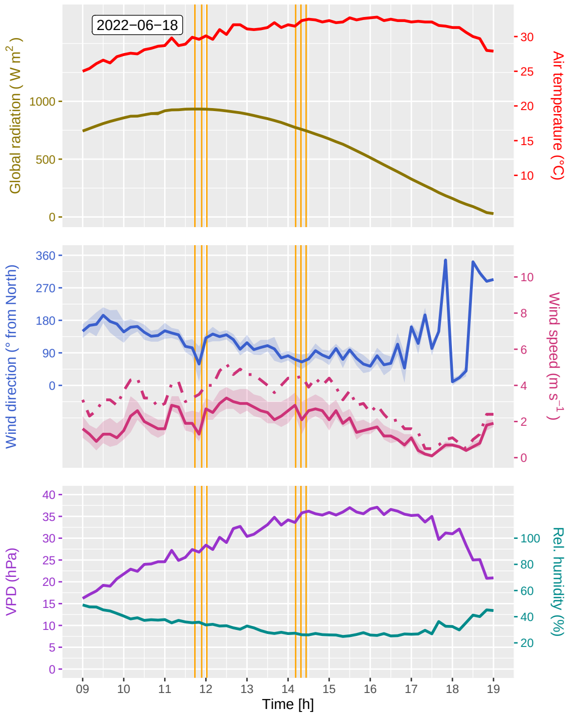

(f) 2022-06-18

Figure S7: (a - f) show the detailed weather conditions on days of measurements in 2022. (cont.)

S20 Multi-view percentile selection

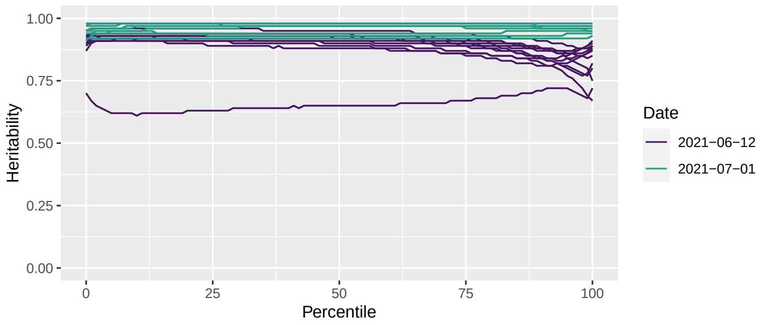

(a) EuVar21

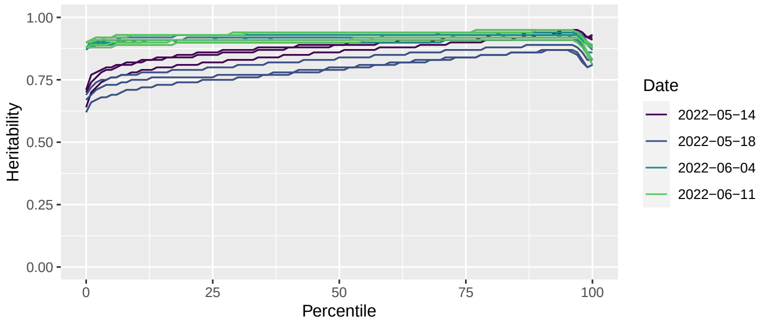

(b) EuVar22

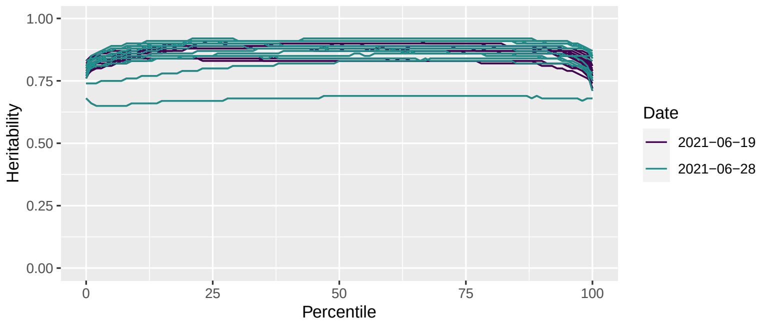

(c) SwiVar21

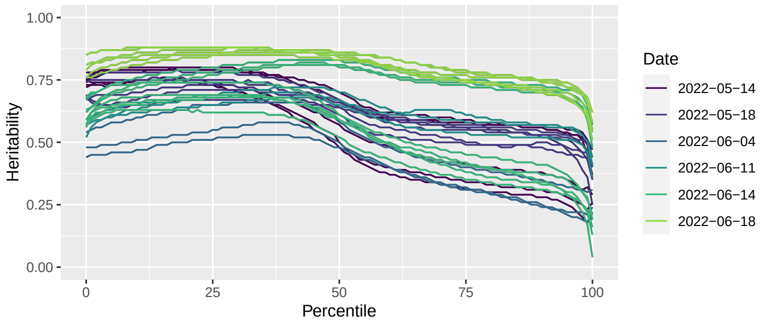

(d) SwiVar22

Figure S8: (a - d) show the heritability of the multi-view method for each pixel value percentile for each flight conducted on EuVar and SwiVar

S21 Correction steps EuVar

S21.1 No correction applied - plot-wise means

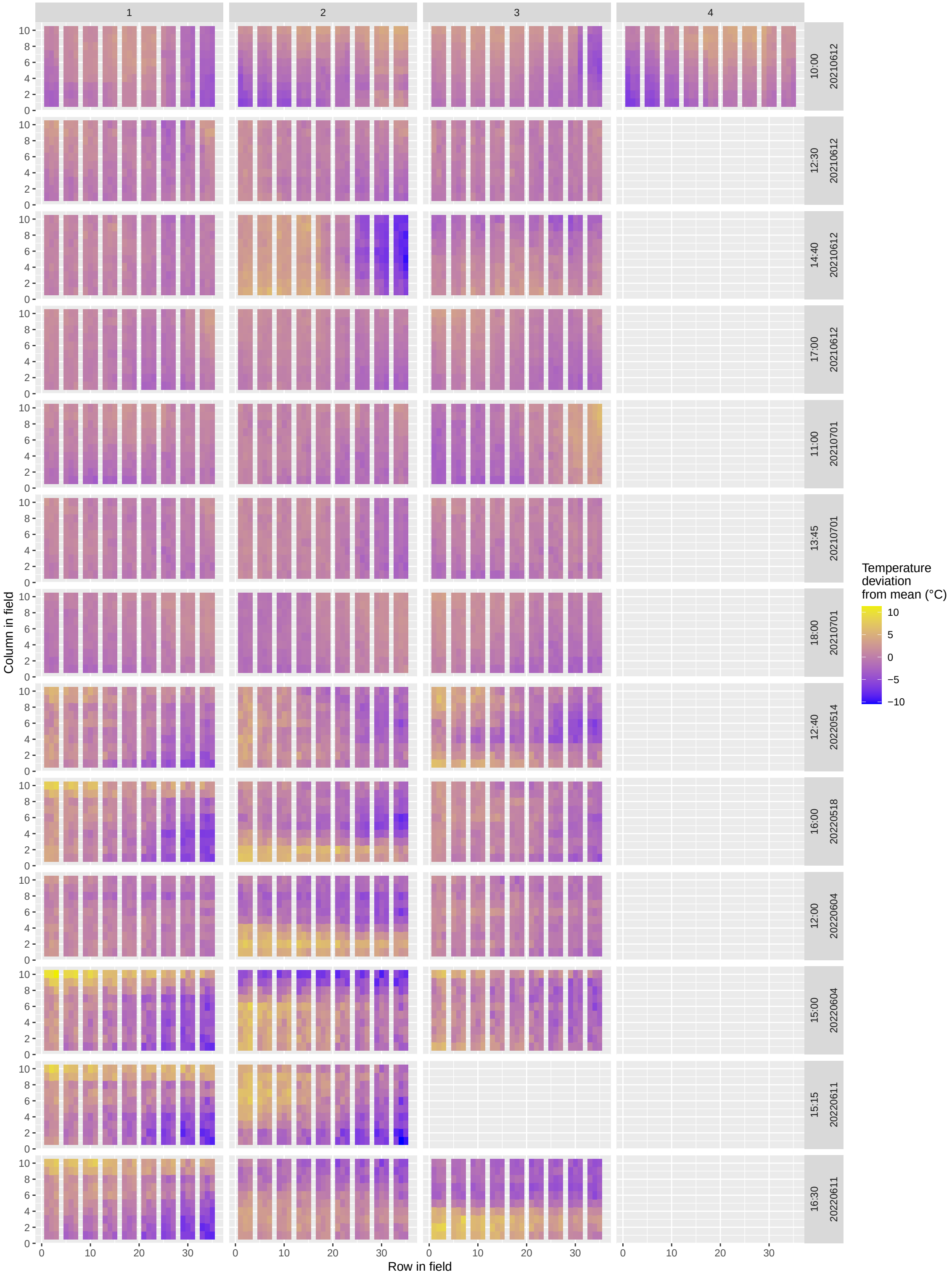

Figure S9: Unadjusted plot-wise means of EuVar. Flights are horizontally grouped by dates and flight times. Each row corresponds to a campaign. Columns indicate the flight order within campaigns. "Column in field" and "Row in field" indicate the spatial position of the plot in in the field where column increases along the tractor track direction. To allow for a meaningful representation of contrasting temperature ranges, flight-wise temperature deviations from flight-wise mean values are shown.

S21.2 Temporal trend estimation

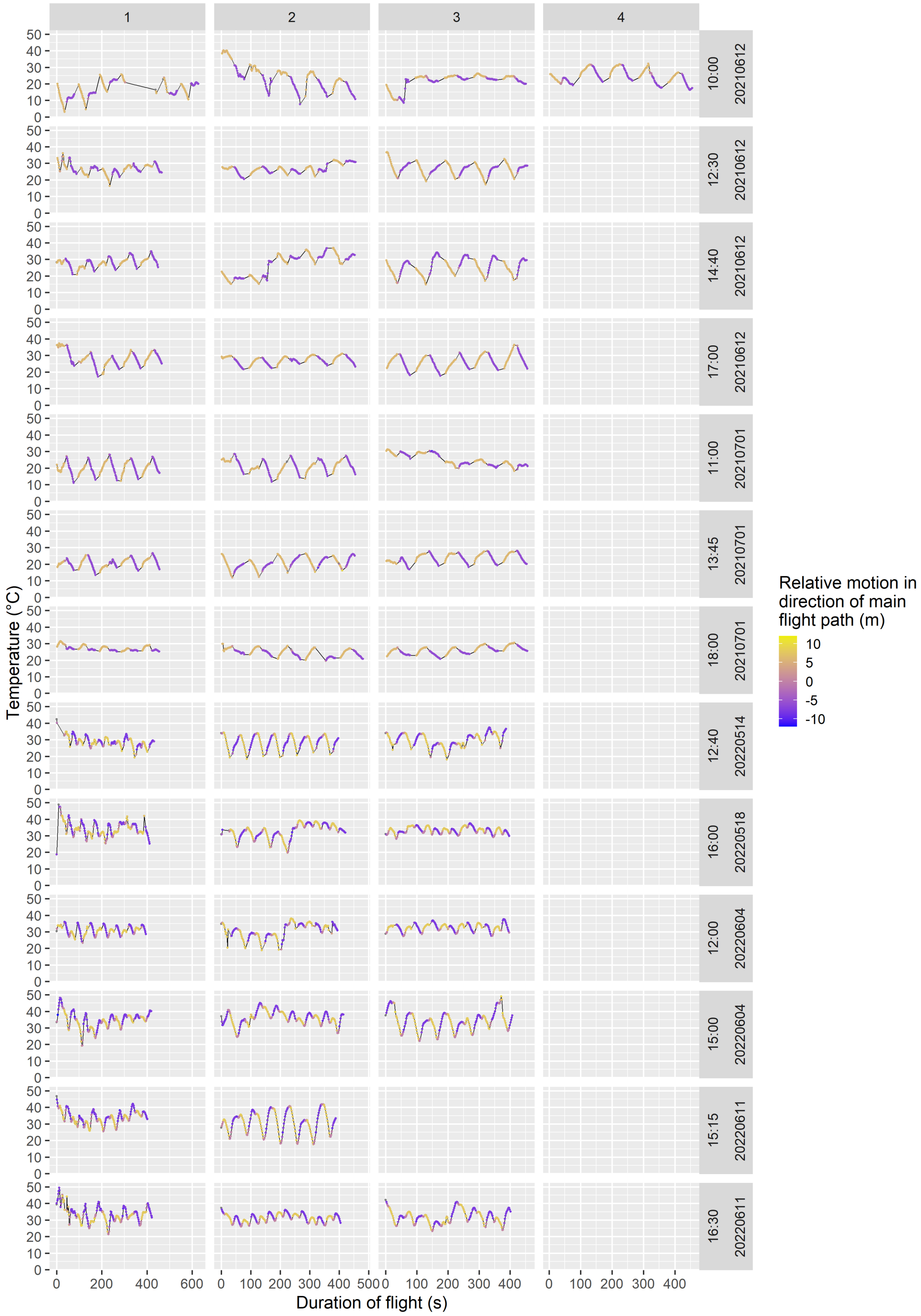

Figure S10: Estimated thermal drift of TIR measurements throughout the duration of fights for all flights of EuVar. Flights are horizontally grouped by dates and flight times. Each row corresponds to a campaign. Columns indicate the flight order within campaigns. The colors indicate the motion in direction of the main fight path. Purple indicates fights in one direction and yellow in the opposite direction of the flight path grid. For gray points, temporal drift was modeled but there was no corresponding measurement of motion along the main fight path.

S21.3 Temporal correction applied

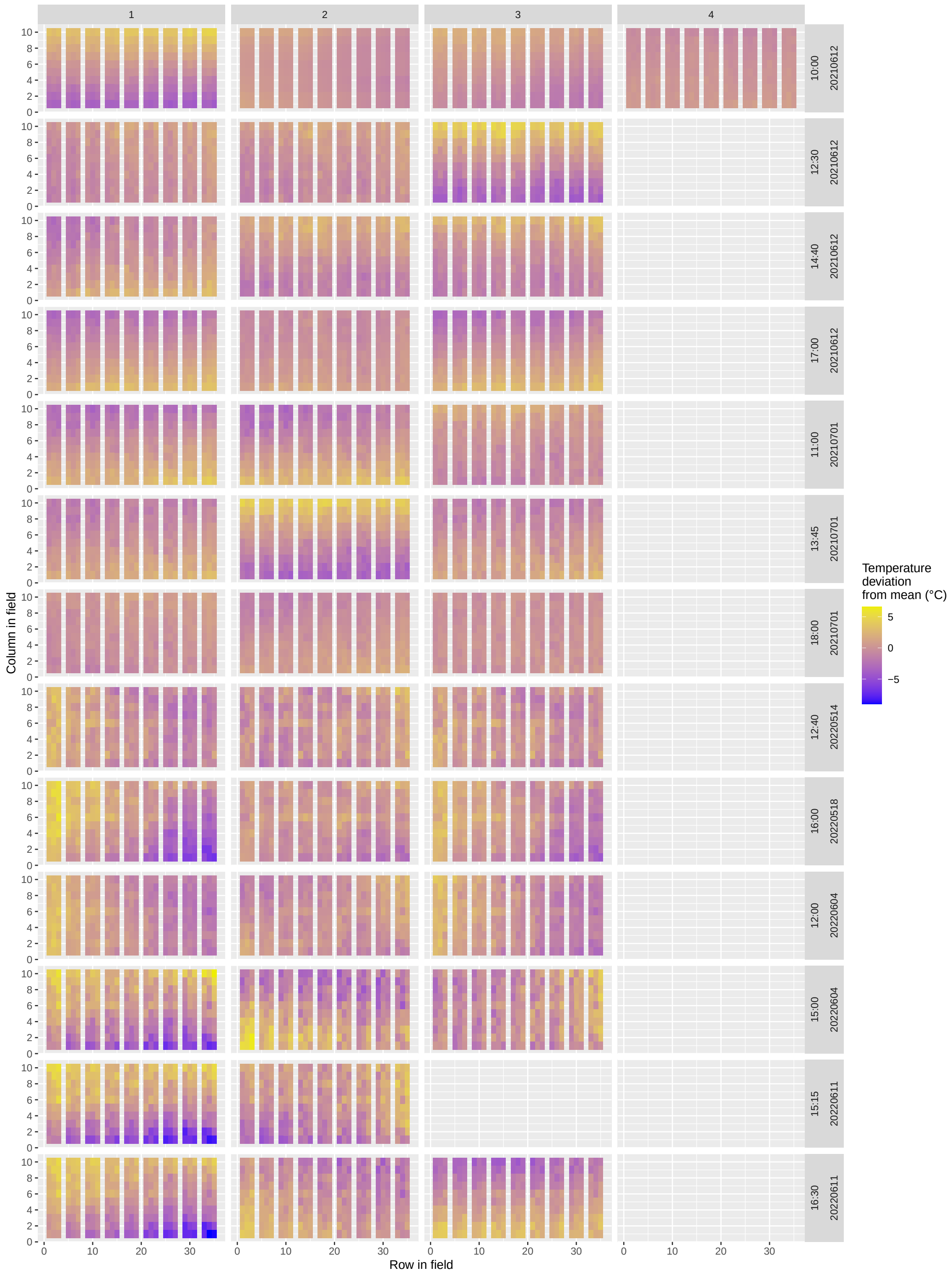

Figure S11: Adjusted plot-wise means of EuVar after a temporal correction. Flights are horizontally grouped by dates and flight times. Each row corresponds to a campaign. Columns indicate the flight order within campaigns."Column in field" and "Row in field" indicate the spatial position of the plot in in the field where column increases along the tractor track direction. To allow for a meaningful representation of contrasting temperature ranges, flight-wise temperature deviations from flight-wise mean values are shown.

S21.4 Temporal and spatial correction applied

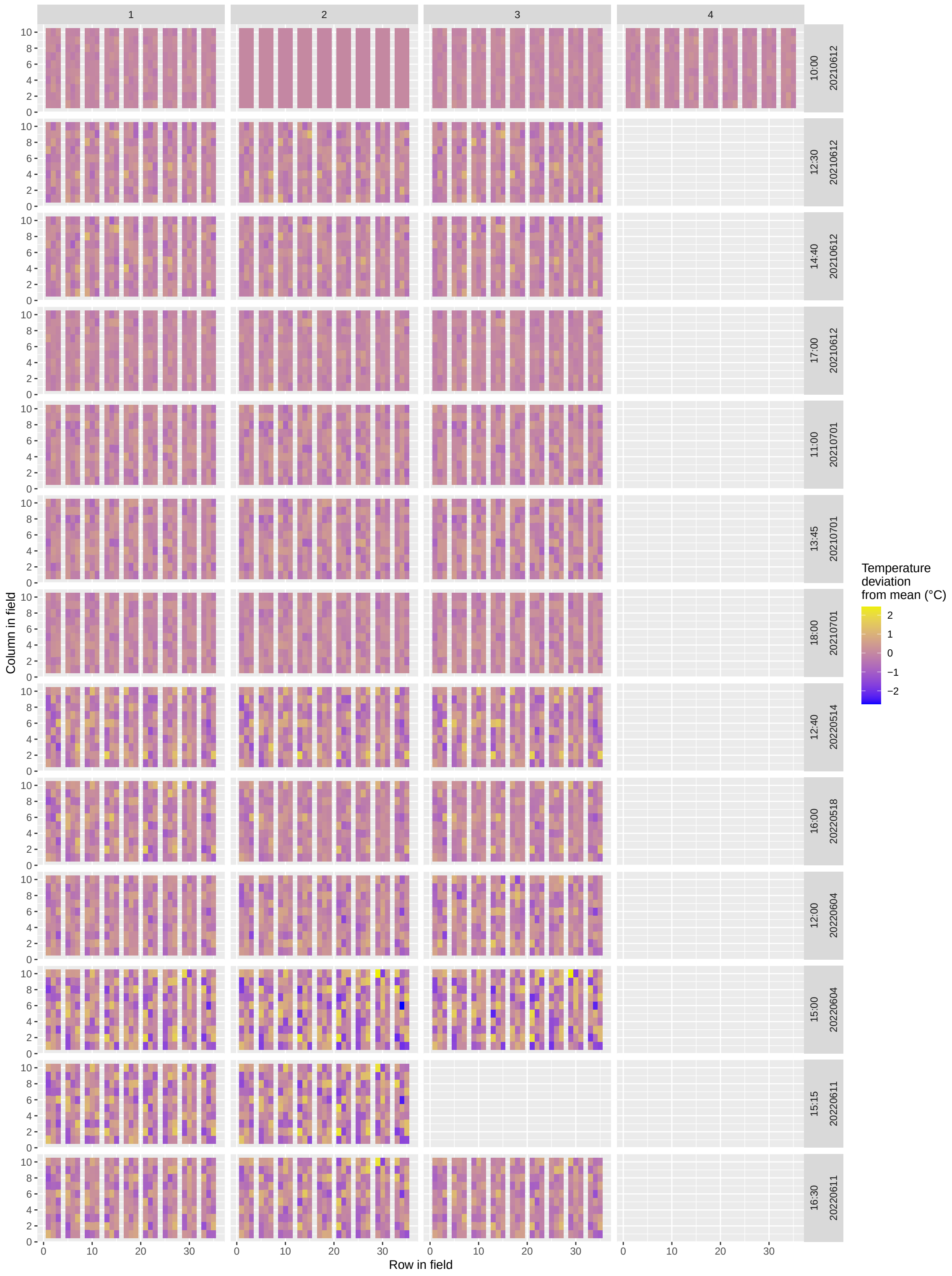

Figure S12: Adjusted plot-wise means of EuVar after a temporal and spatial correction. Flights are horizontally grouped by dates and flight times. Each row corresponds to a campaign. Columns indicate the flight order within campaigns. “Column in field” and “Row in field” indicate the spatial position of the plot in in the field where column increases along the tractor track direction. To allow for a meaningful representation of contrasting temperature ranges, flight-wise temperature deviations from flight-wise mean values are shown.

S21.5 Genotypic effect (correction applied for effects of trigger timing, field heterogeneity, plots and treatment regimens)

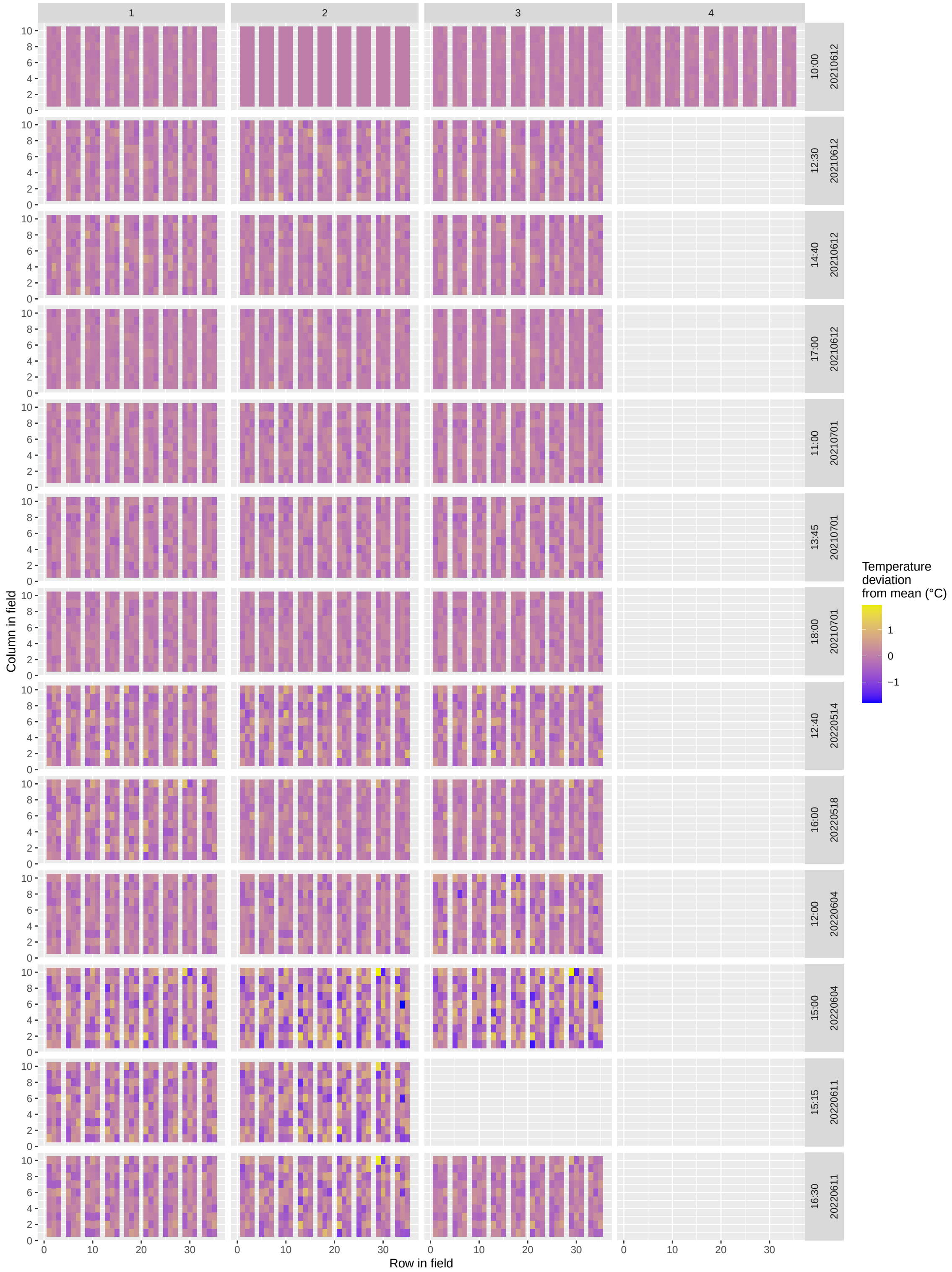

Figure S13: Estimated effect for the single genotypes and genotype-treatment interactions for all flights flown on EuVar. Temporal correction, spatial correction and treatment deflation were applied. Flights are horizontally grouped by dates and flight times. Each row corresponds to a campaign. Columns indicate the flight order within campaigns. "Column in field" and "Row in field" indicate the spatial position of the plot in in the field where column increases along the tractor track direction. To allow for a meaningful representation of contrasting temperature ranges, flight-wise temperature deviations from flight-wise mean values are shown.

S21.6 Treatment effect (correction applied for effects of trigger timing, field heterogeneity, plots and genotypes)

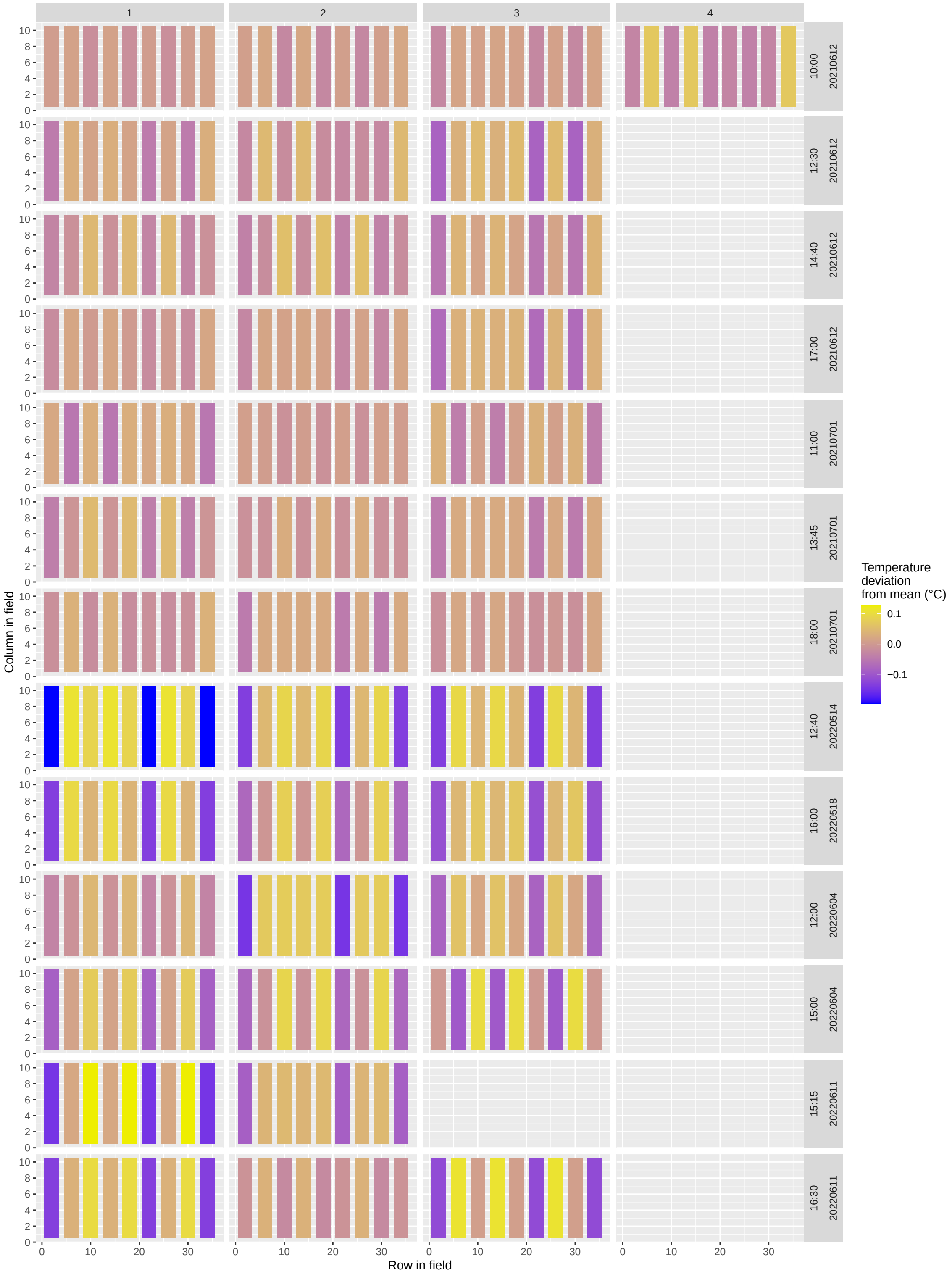

Figure S14: Estimated effect for the treatment regimens for all flights flown on EuVar. Flights are horizontally grouped by dates and flight times. Each row corresponds to a campaign. Columns indicate the flight order within campaigns. "Column in field" and "Row in field" indicate the spatial position of the plot in in the field where column increases along the tractor track direction. To allow for a meaningful representation of contrasting temperature ranges, flight-wise temperature deviations from flight-wise mean values are shown.

S22 Correction steps SwiVar

S22.1 No correction applied - plot-wise means

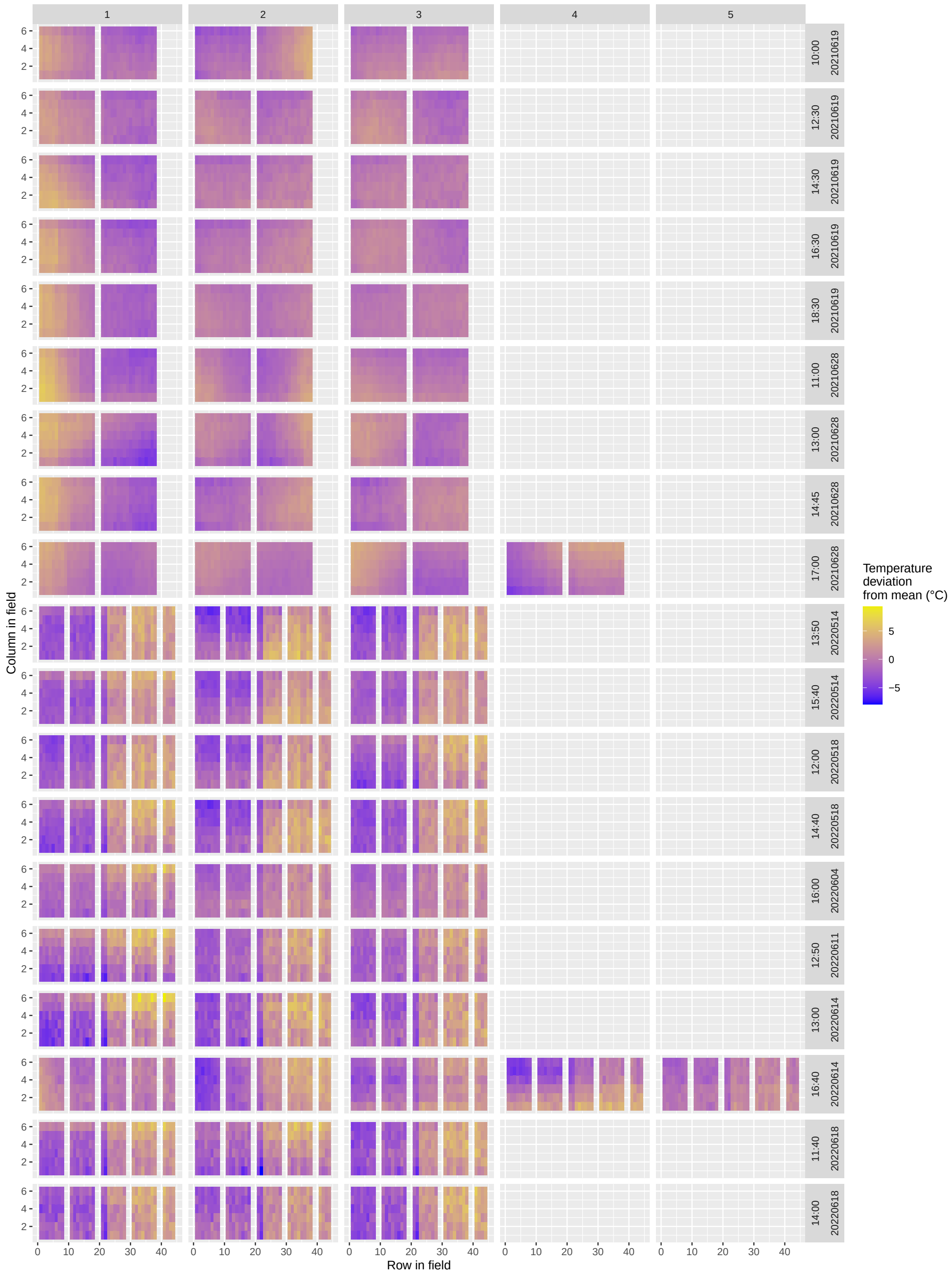

Figure S15: Unadjusted plot-wise means of SwiVar. Flights are horizontally grouped by dates and flight times. Each row corresponds to a campaign. Columns indicate the flight order within campaigns. “Column in field” and “Row in field” indicate the spatial position of the plot in in the field where column increases along the tractor track direction. To allow for a meaningful representation of contrasting temperature ranges, flight-wise temperature deviations from flight-wise mean values are shown.

S22.2 Temporal trend estimation

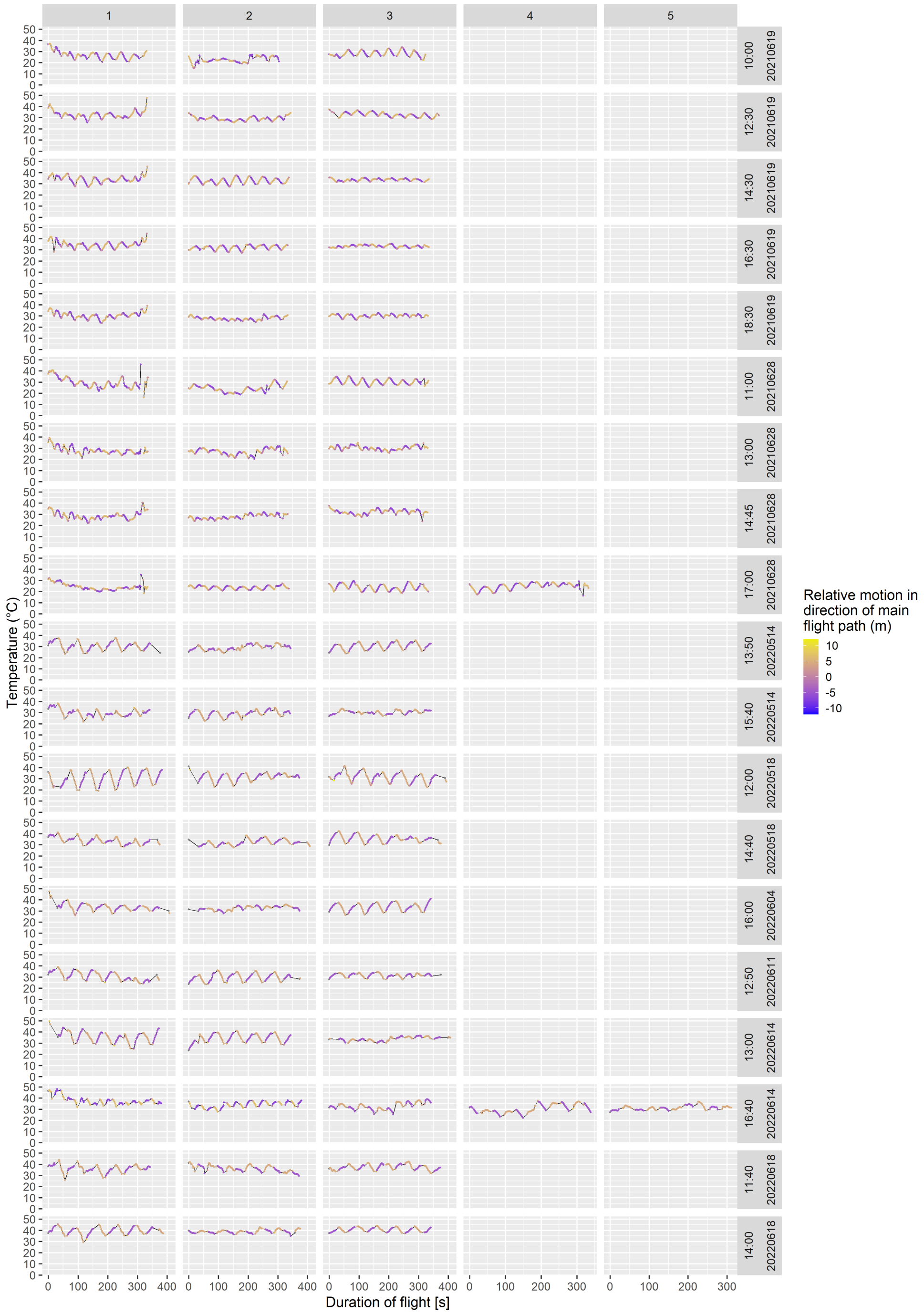

Figure S16: Estimated thermal drift of TIR measurements throughout the duration of fights for all flights of SwiVar. Flights are horizontally grouped by dates and flight times. Each row corresponds to a campaign. Columns indicate the flight order within campaigns. The colors indicate the motion in direction of the main fight path. Purple indicates fights in one direction and yellow in the opposite direction of the flight path grid. For gray points, temporal drift was modeled but there was no corresponding measurement of motion along the main fight path.

S22.3 Temporal correction applied

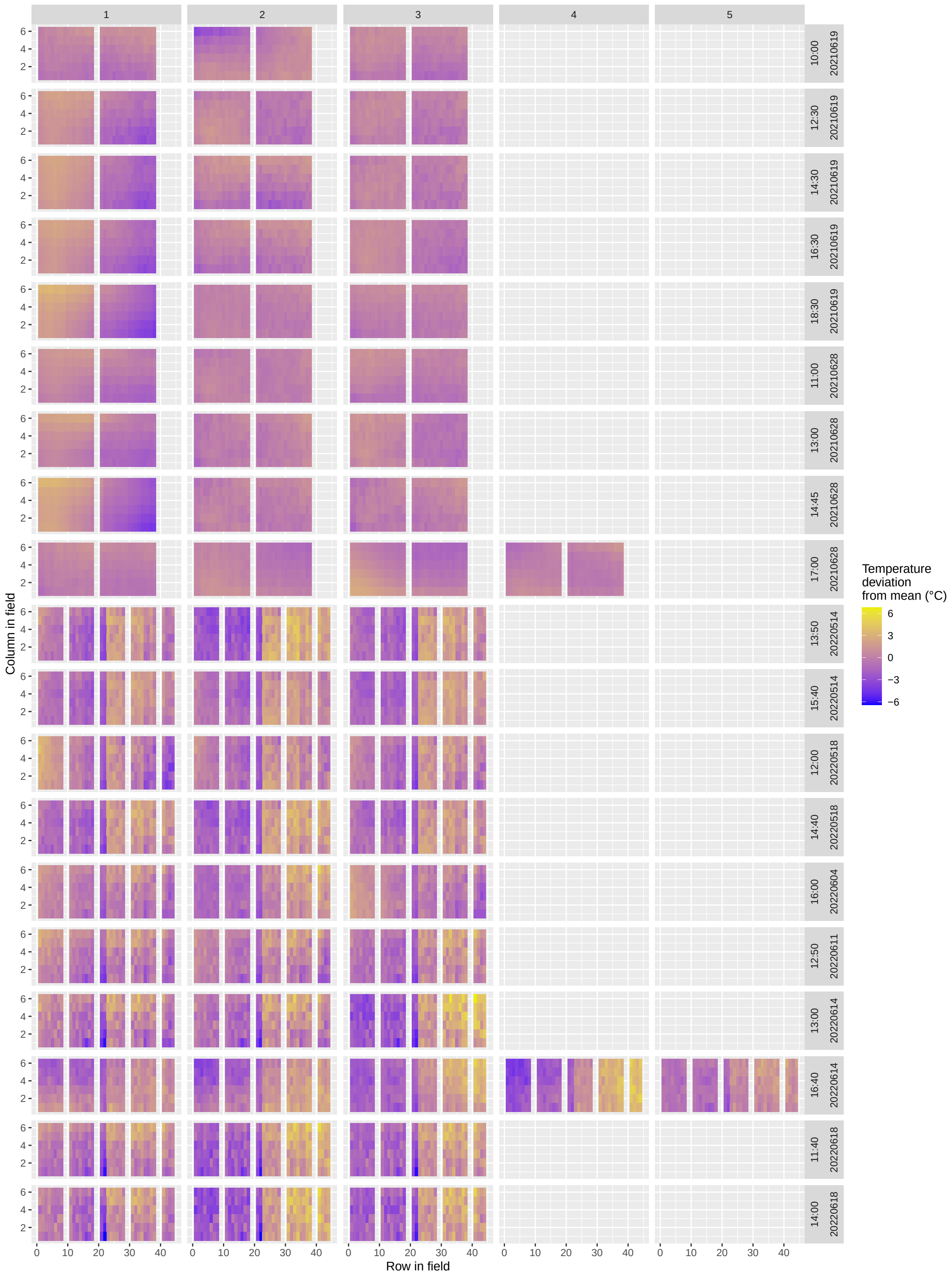

Figure S17: Adjusted plot-wise means of SwiVar after a temporal correction. Flights are horizontally grouped by dates and flight times. Each row corresponds to a campaign. Columns indicate the flight order within campaigns. "Column in field" and "Row in field" indicate the spatial position of the plot in in the field where column increases along the tractor track direction. To allow for a meaningful representation of contrasting temperature ranges, flight-wise temperature deviations from flight-wise mean values are shown.

S22.4 Temporal and spatial correction applied

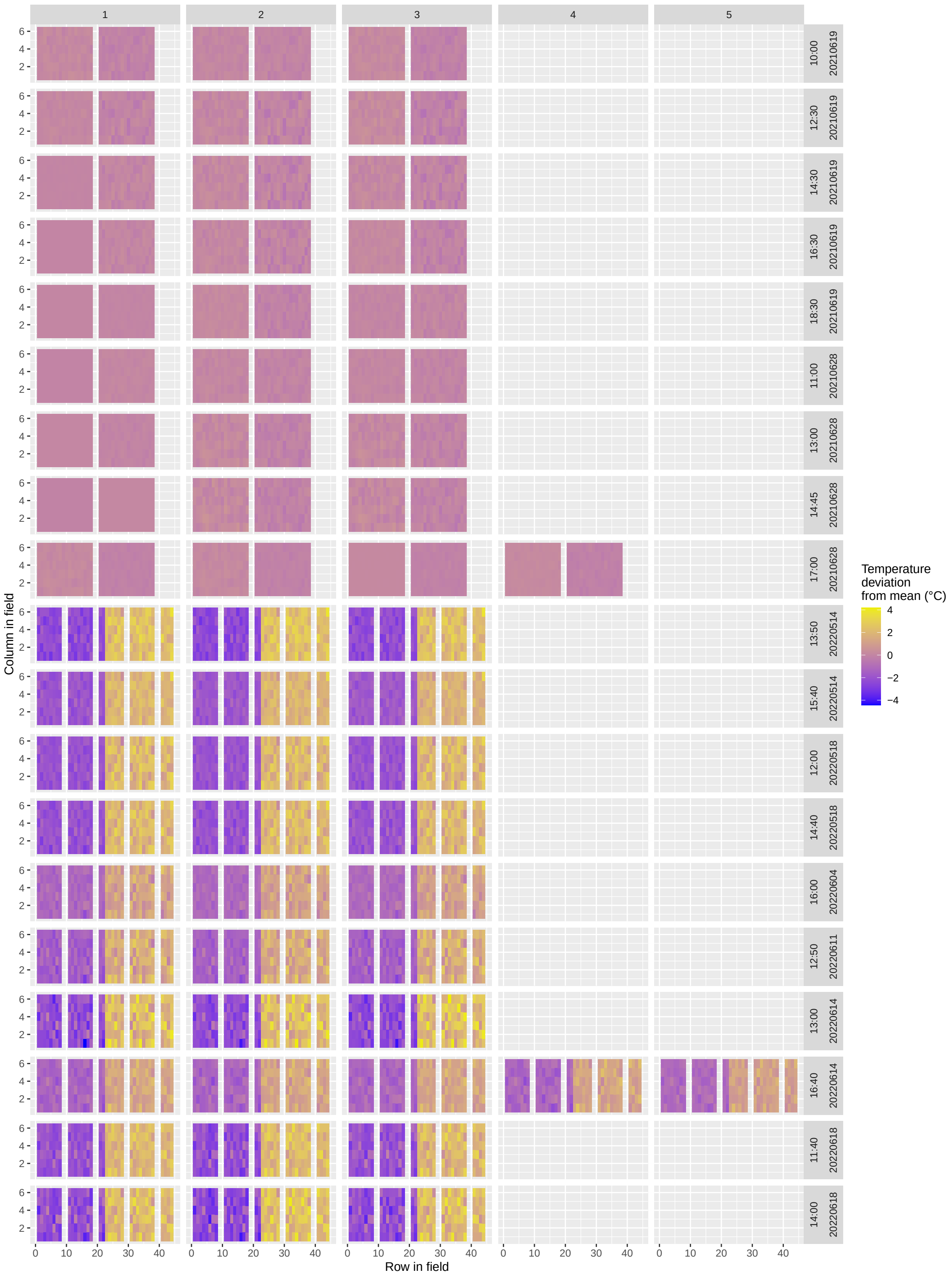

Figure S18: Adjusted plot-wise means of SwiVar after a temporal and spatial correction. Flights are horizontally grouped by dates and flight times. Each row corresponds to a campaign. Columns indicate the flight order within campaigns. "Column in field" and "Row in field" indicate the spatial position of the plot in in the field where column increases along the tractor track direction. To allow for a meaningful representation of contrasting temperature ranges, flight-wise temperature deviations from flight-wise mean values are shown.

S22.5 Genotypic effect (correction applied for effects of trigger timing, field heterogeneity, plots and treatment regimens)

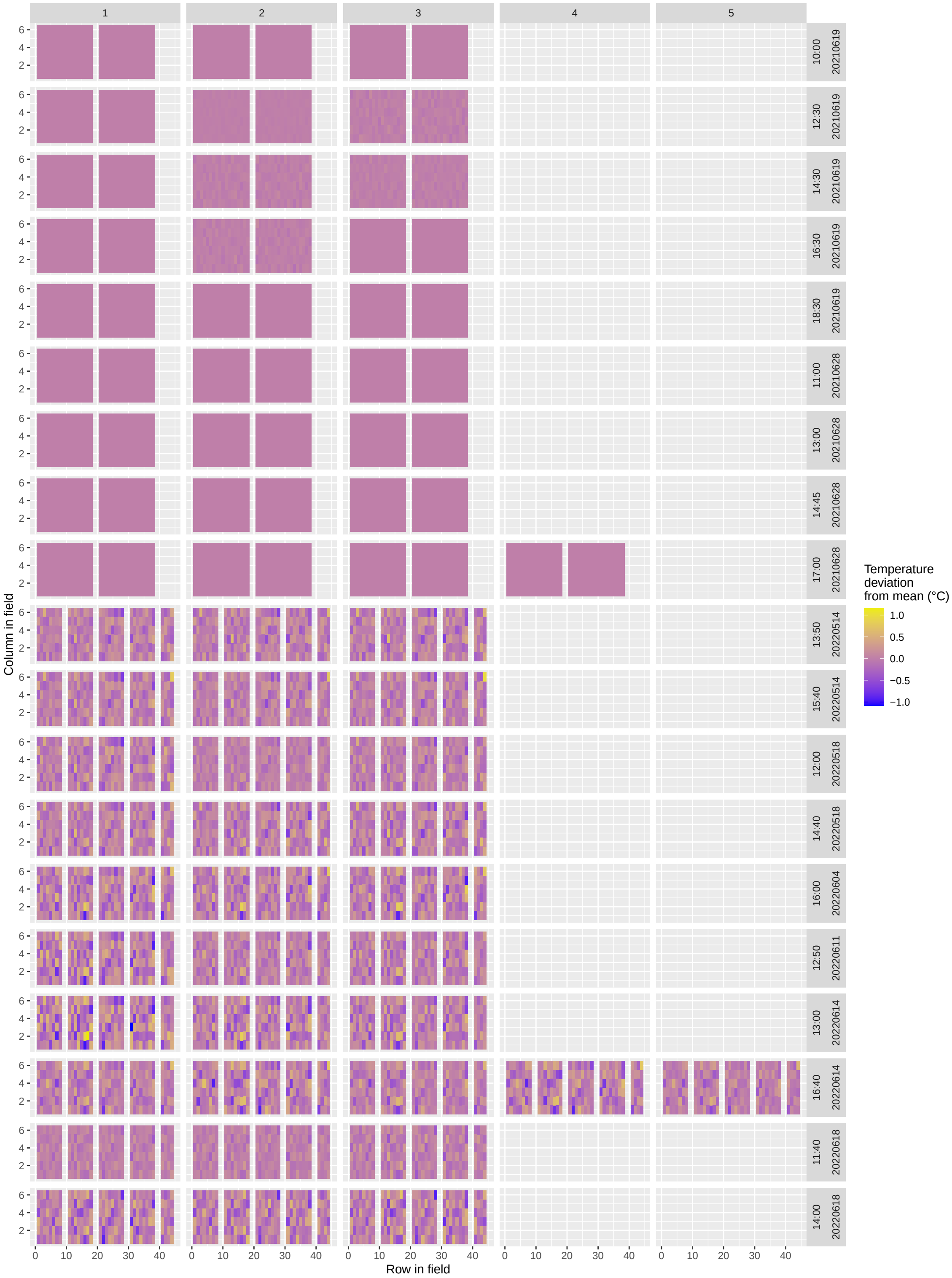

Figure S19: Estimated effect for the single genotypes and genotype-treatment interactions for all flights flown on SwiVar. Temporal correction, spatial correction and treatment deflation were applied. Flights are horizontally grouped by dates and flight times. Each row corresponds to a campaign. Columns indicate the flight order within campaigns. “Column in field” and “Row in field” indicate the spatial position of the plot in in the field where column increases along the tractor track direction. To allow for a meaningful representation of contrasting temperature ranges, flight-wise temperature deviations from flight-wise mean values are shown.

S22.6 Treatment effect (correction applied for effects of trigger timing, field heterogeneity, plots and genotypes)

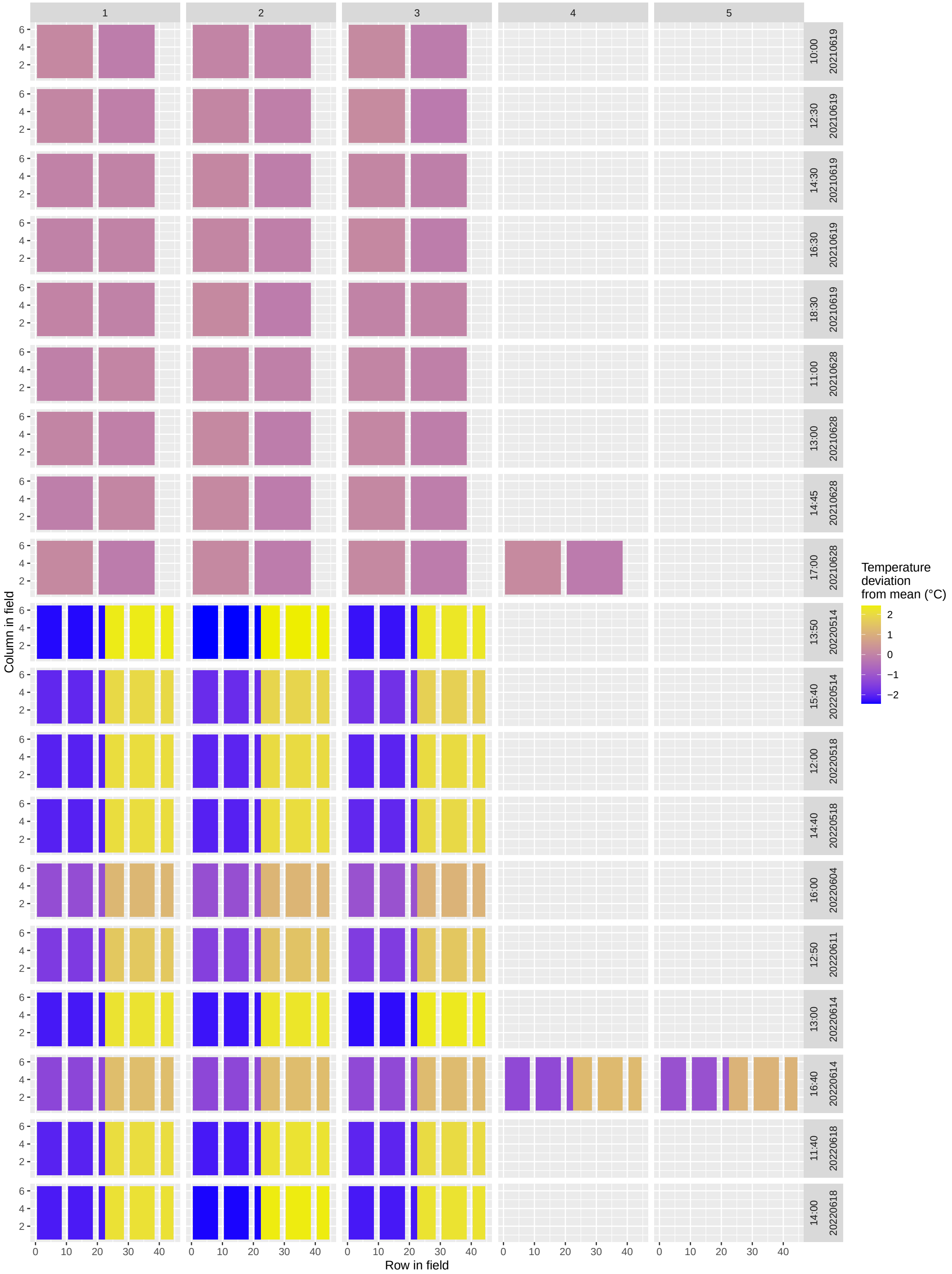

Figure S20: Estimated effect for the treatment regimens for all flights flown on SwiVar. Flights are horizontally grouped by dates and flight times. Each row corresponds to a campaign. Columns indicate the flight order within campaigns. "Column in field" and "Row in field" indicate the spatial position of the plot in in the field where column increases along the tractor track direction. To allow for a meaningful representation of contrasting temperature ranges, flight-wise temperature deviations from flight-wise mean values are shown.

S23 Uncorrected phenotypic traits

EuVar21

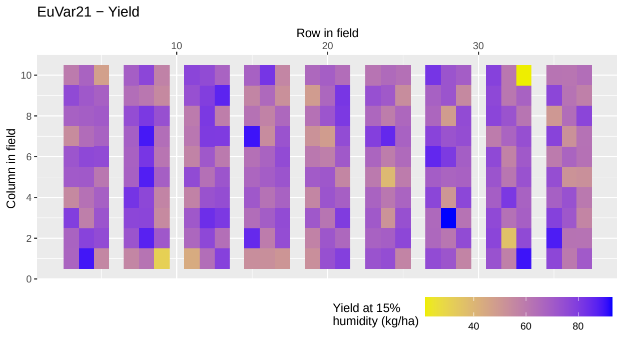

(a)

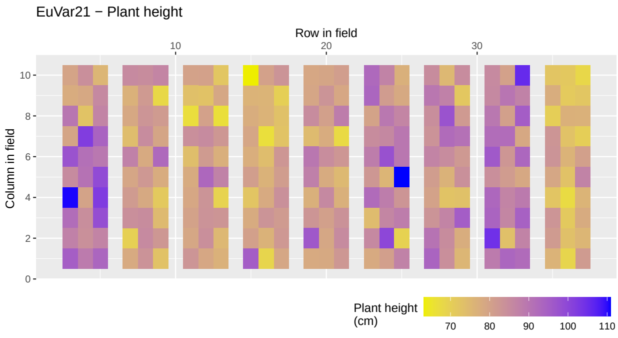

(b)

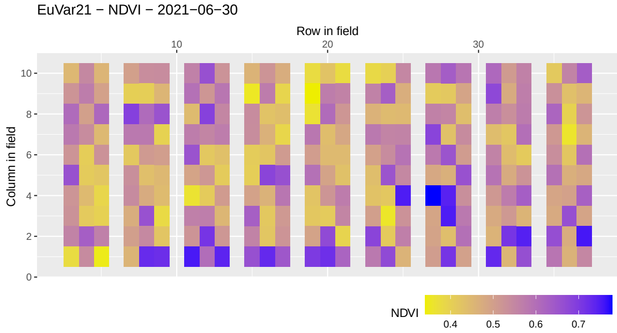

(c)

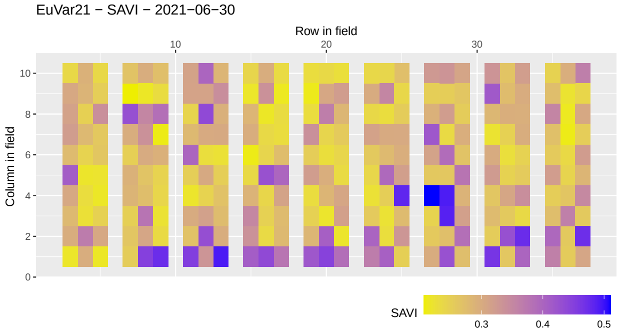

(d)

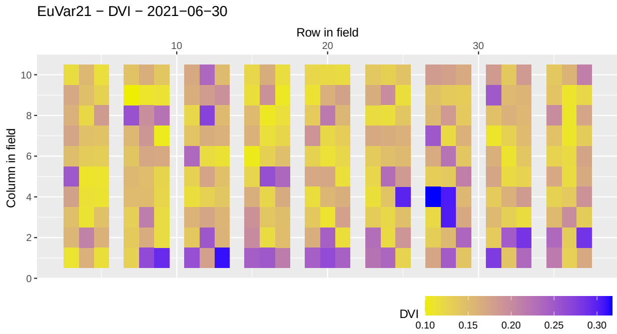

(e)

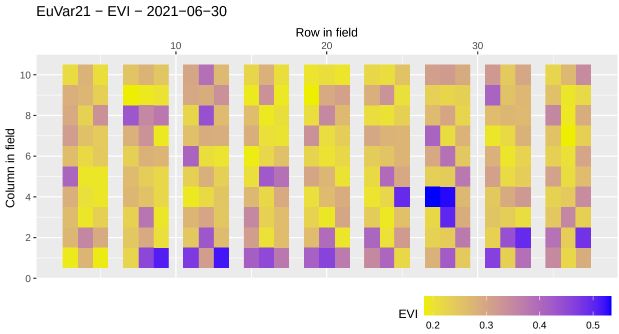

(f)

Figure S21: (a - f) show the uncorrected phenotypic traits of EuVar21. (a): Yield at 15 % water content, (b): Plant height based on five measurements per plot, (c): NDVI multispectral index, (d): SAVI multispectral index, (e): DVI multispectral index, (f): EVI multispectral index.

EuVar22

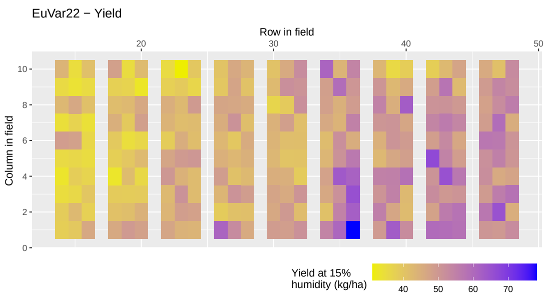

(a)

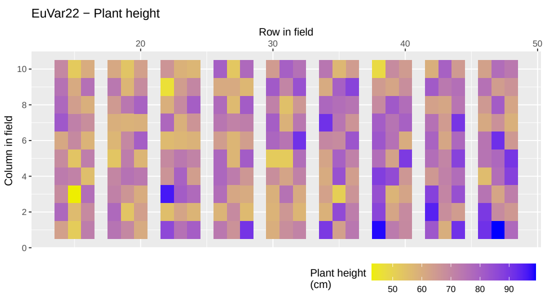

(b)

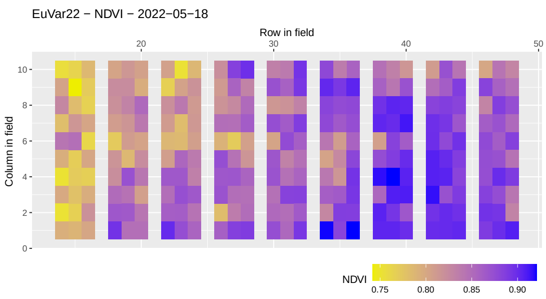

(c)

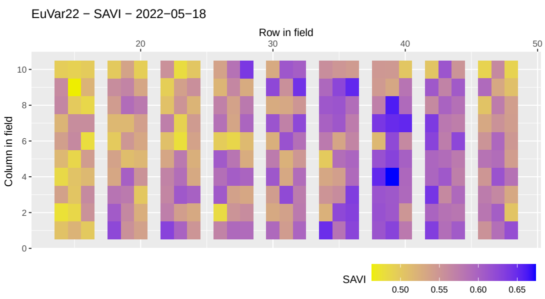

(d)

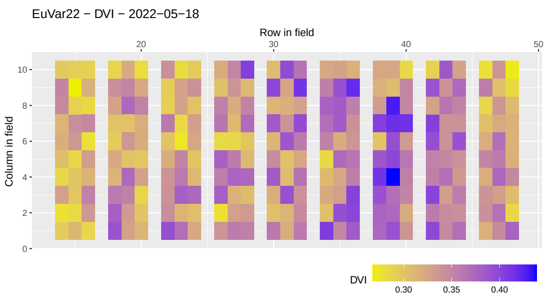

(e)

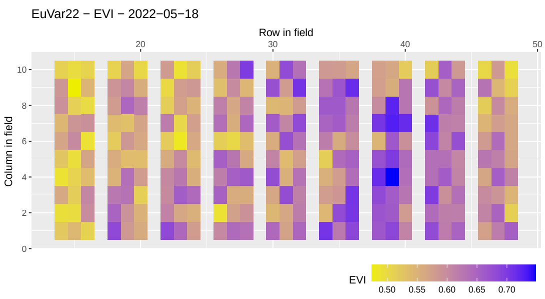

(f)

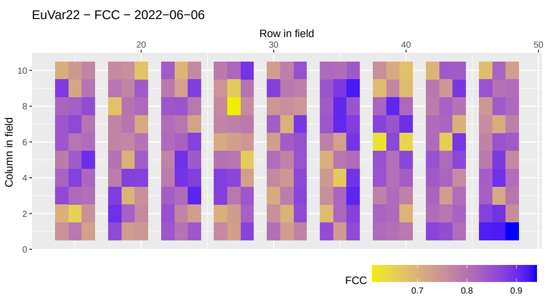

(g)

Figure S22: (a - g) show the uncorrected phenotypic traits of EuVar22. (a): Yield at 15 % water content, (b): Planth height based on five measurements per plot, (c): NDVI multispectral index, (d): SAVI multispectral index, (e): DVI multispectral index, (f): EVI multispectral index, (g): FCC.

SwiVar21

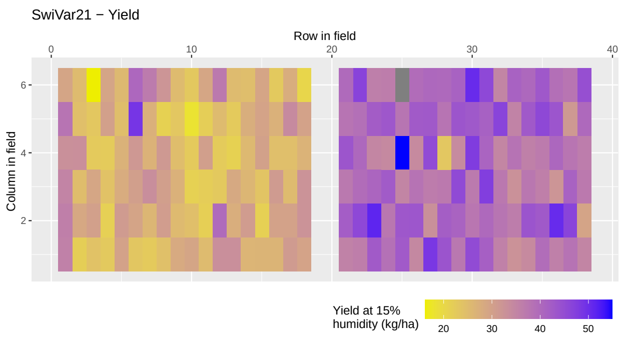

(a)

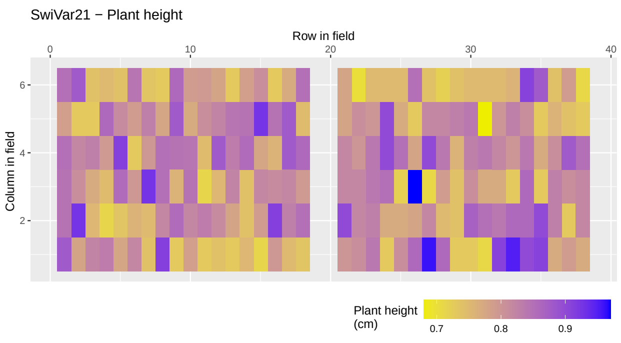

(b)

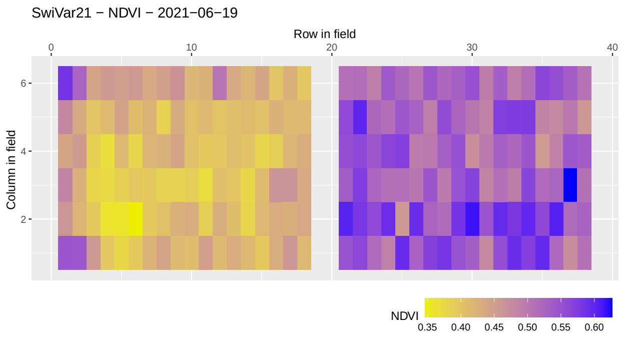

(c)

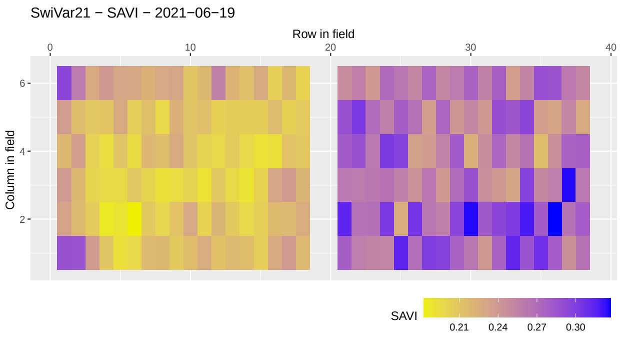

(d)

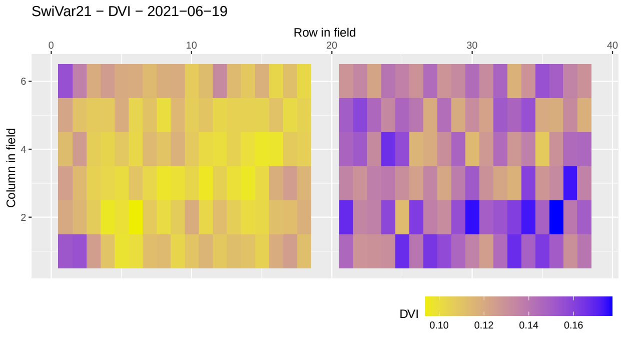

(e)

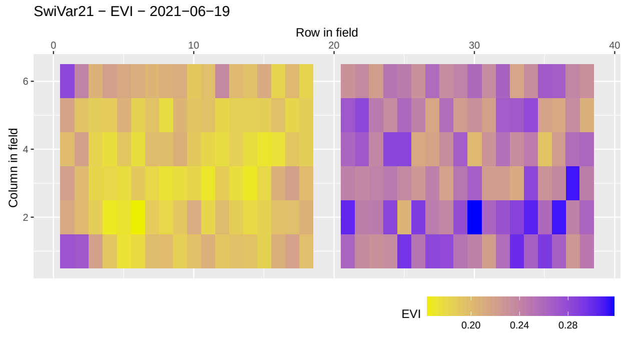

(f)

Figure S23: (a - f) show the uncorrected phenotypic traits of SwiVar21. (a): Yield at 15 % water content, (b): Planth height based on five measurements per plot, (c): NDVI multispectral index, (d): SAVI multispectral index, (e): DVI multispectral index, (f): EVI multispectral index.

SwiVar22

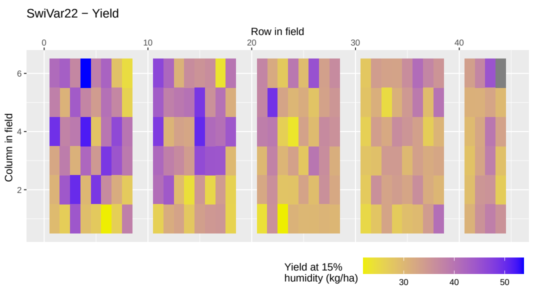

(a)

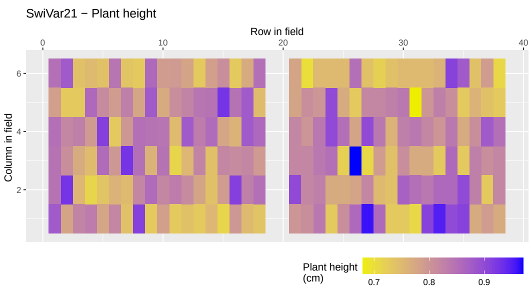

(b)

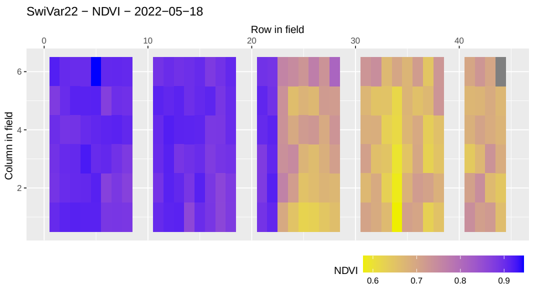

(c)

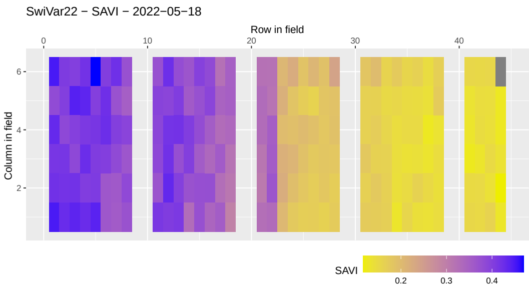

(d)

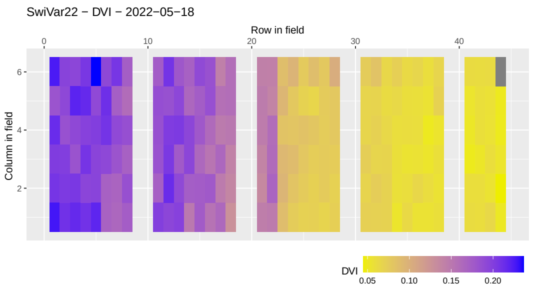

(e)

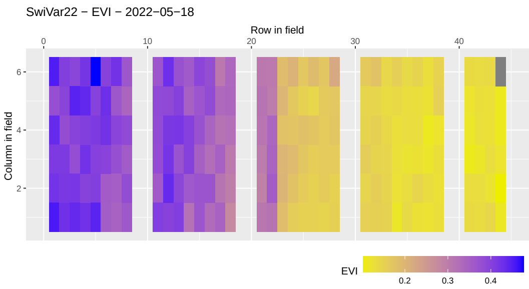

(f)

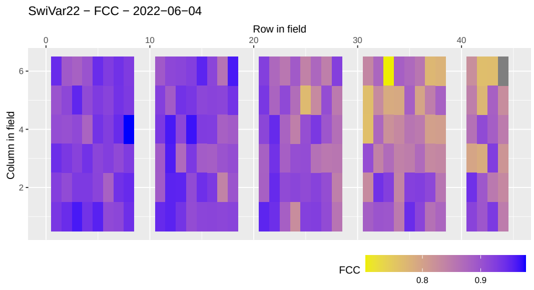

(g)

Figure S24: (a - g) show the uncorrected phenotypic traits of SwiVar22. (a): Yield at 15 % water content, (b): Planth height based on five measurements per plot, (c): NDVI multispectral index, (d): SAVI multispectral index, (e): DVI multispectral index, (f): EVI multispectral index, (g): FCC. On the plot in grey, a sowing error occurred and the plot was excluded from analysis.

S24 Correlation with yield based on campaign-wise CT estimates

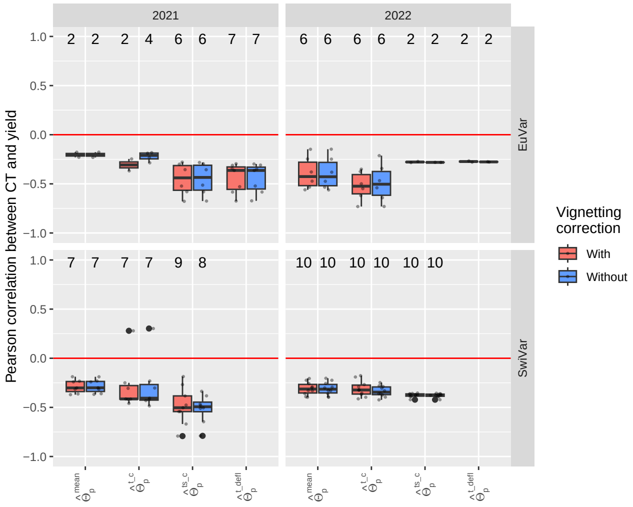

Figure S25: The CT estimates based on all flights within campaigns without and with vignetting correction were correlated to yield. Just correlations significant at $p \leq 0.01$ are shown. The number above the boxplots indicates the number of campaigns with significant correlations included in the respective box plots.

S25 Spatial trend estimates

S25.1 Spatial trend estimation EuVar - individual flights

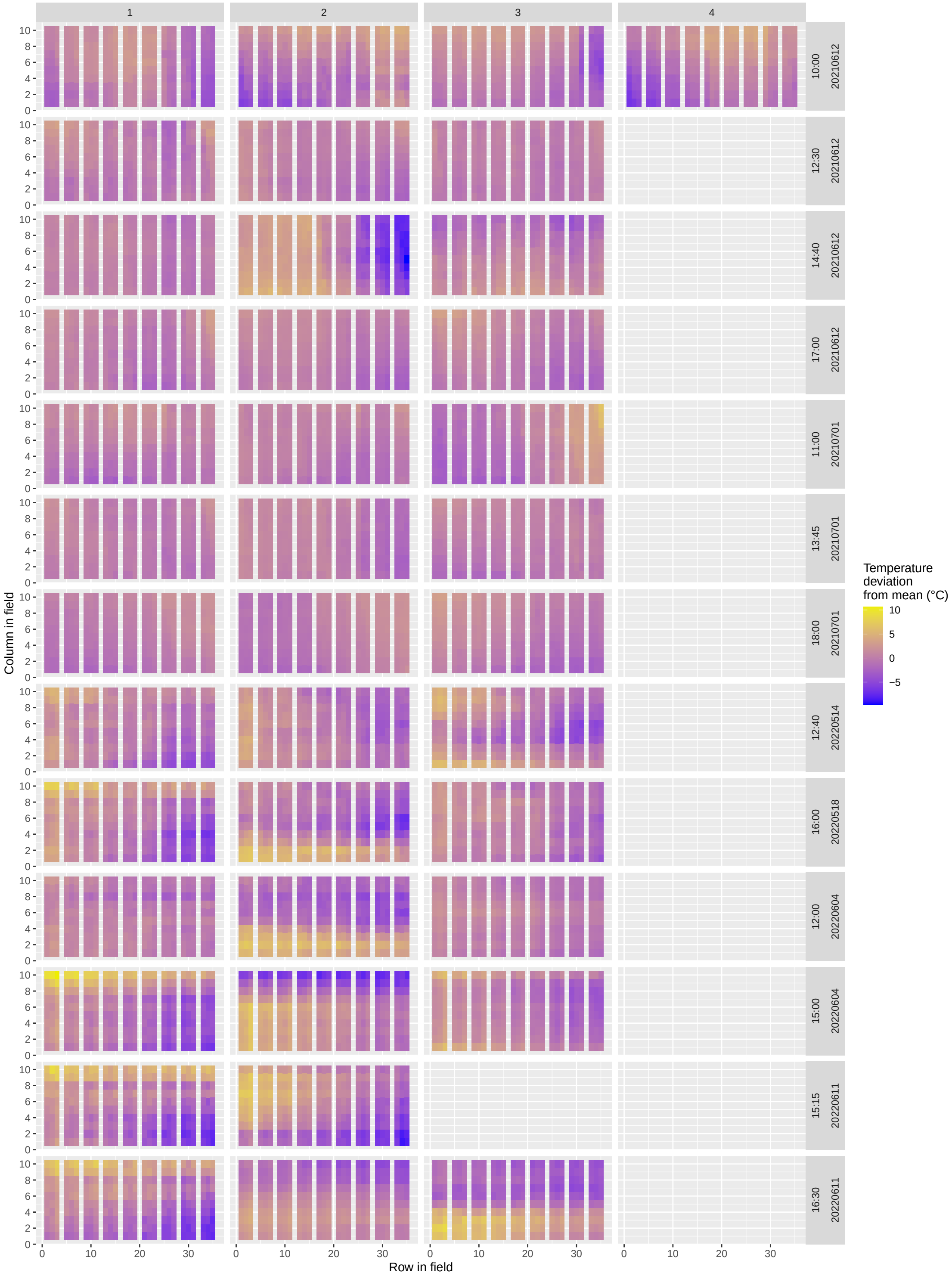

Figure S26: Spatial trend estimates for single flights based on Eq. 10 for EuVar. Flights are horizontally grouped by dates and flight times. Each row corresponds to a campaign. Columns indicate the flight order within campaigns. "Column in field" and "Row in field" indicate the spatial position of the plot in in the field where column increases along the tractor track direction. To allow for a meaningful representation of contrasting temperature ranges, flight-wise temperature deviations from flight-wise mean values are shown.

S25.2 Spatial trend estimation SwiVar - individual flights

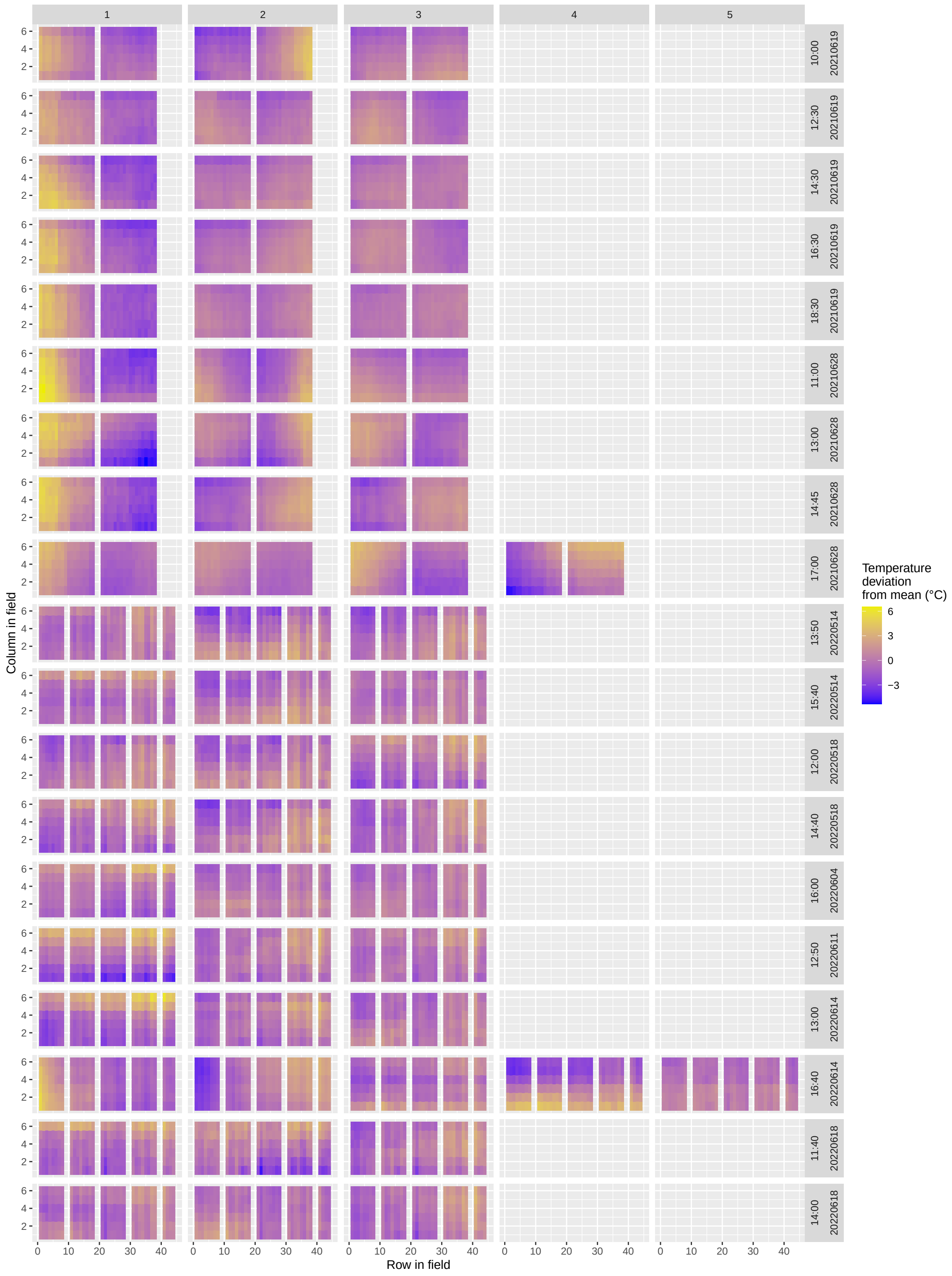

Figure S27: Spatial trend estimates for single flights based on Eq. 10 for SwiVar. Flights are horizontally grouped by dates and flight times. Each row corresponds to a campaign. Columns indicate the flight order within campaigns. "Column in field" and "Row in field" indicate the spatial position of the plot in in the field where column increases along the tractor track direction. To allow for a meaningful representation of contrasting temperature ranges, flight-wise temperature deviations from flight-wise mean values are shown.

S26 Correlation of plot-wise estimates of spatial field trends for individual flights - EuVar 2021

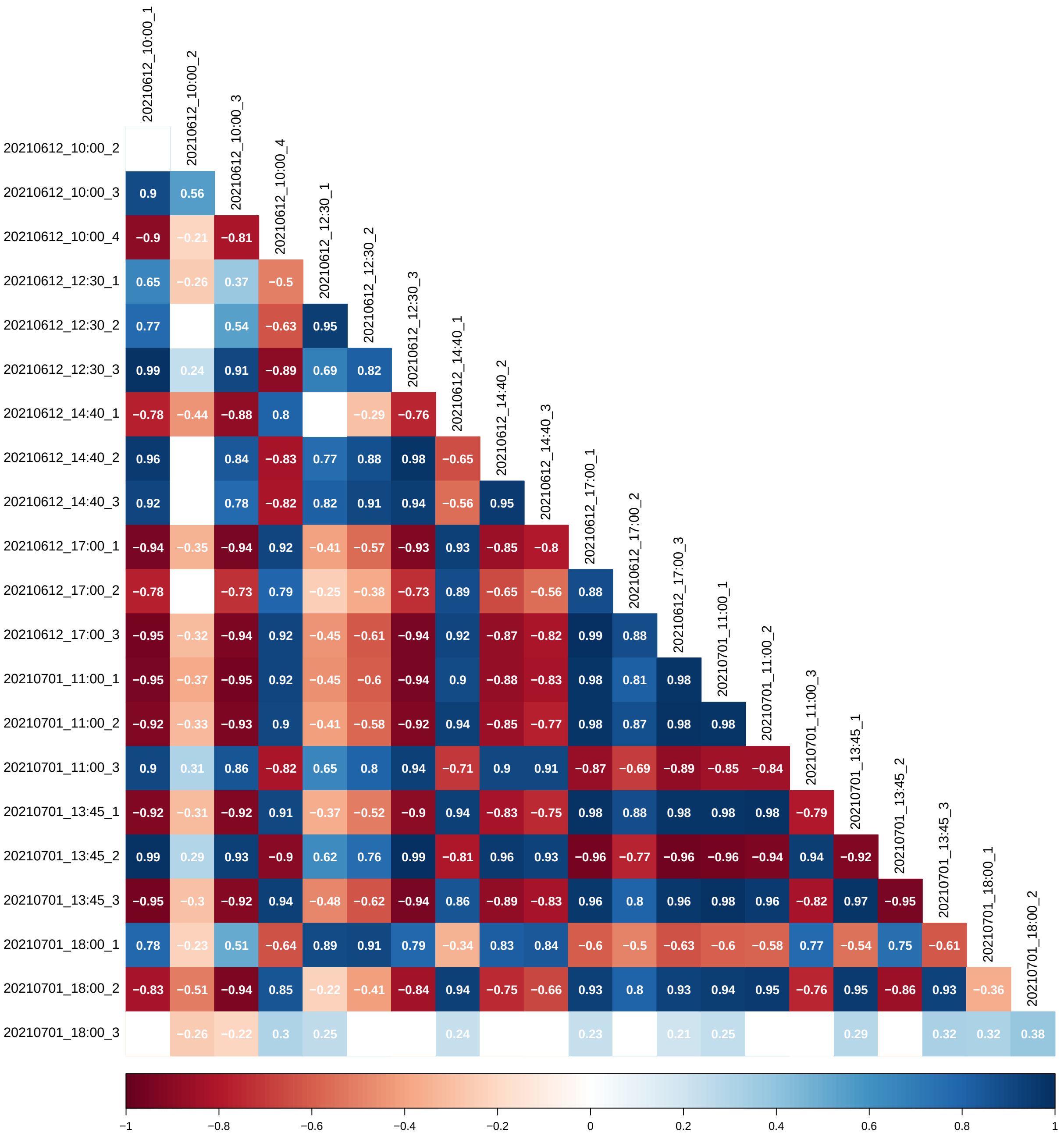

Figure S28: Pearson correlation of plot-wise estimates of spatial trends for EuVar21 which are shown in Fig. S26. Just correlations significant at $p < 0.001$ are shown.

S27 Correlation of plot-wise estimates of spatial field trends for individual flights - EuVar 2022

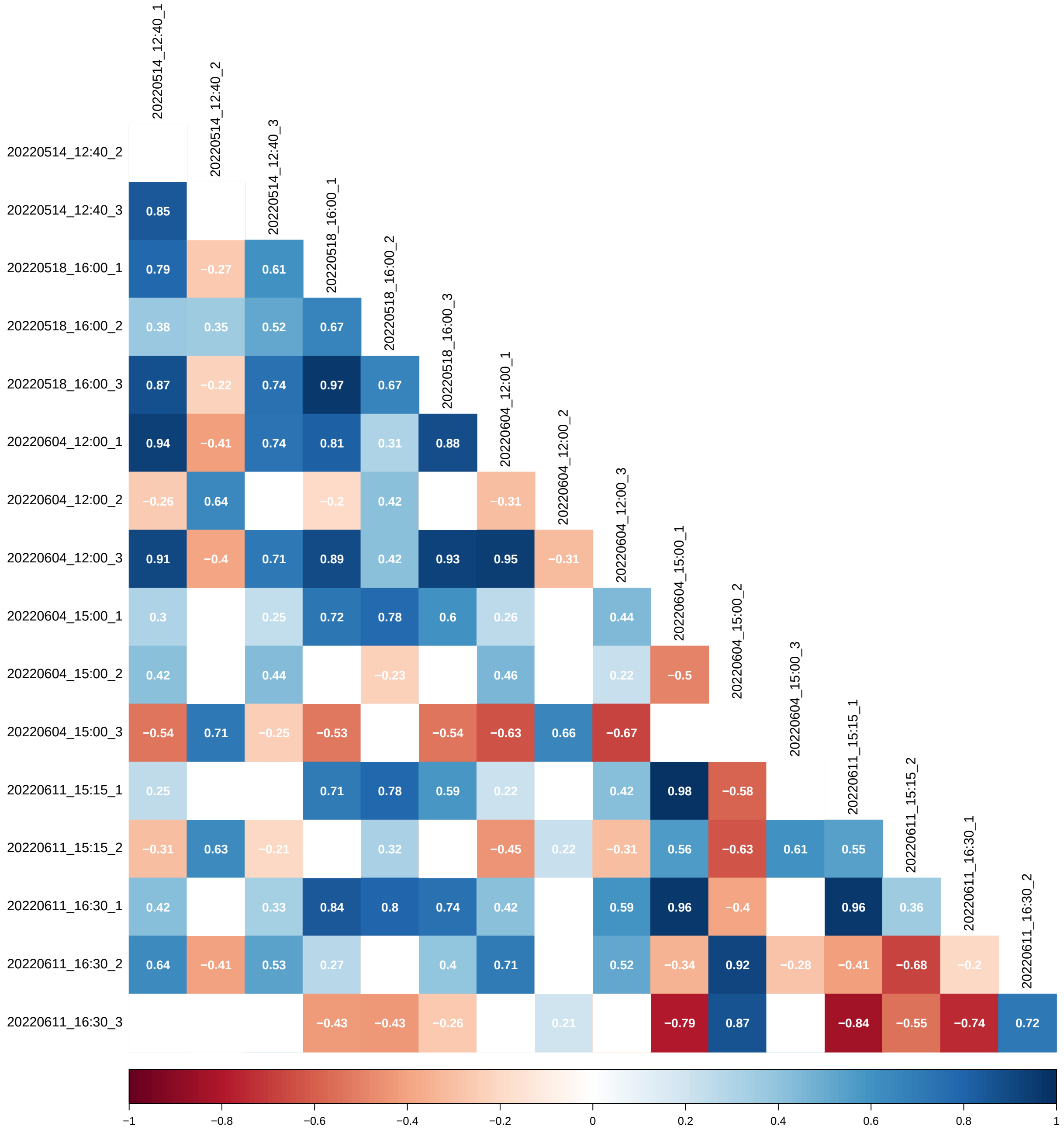

Figure S29: Pearson correlation of plot-wise estimates of spatial trends for EuVar22 which are shown in Fig. S26. Just correlations significant at $p < 0.001$ are shown.

S28 Correlation of plot-wise estimates of spatial field trends for individual flights - SwiVar 2021

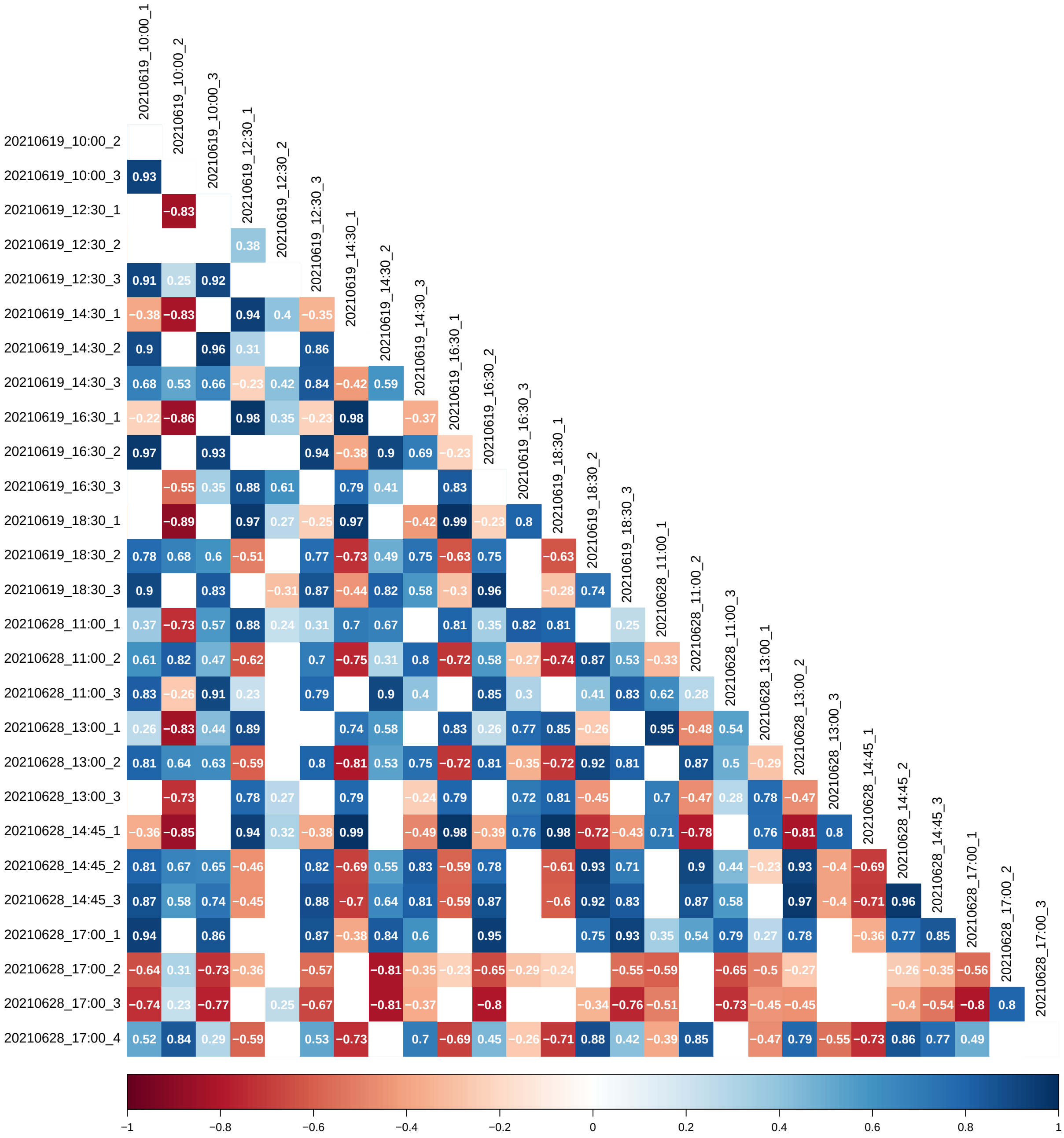

Figure S30: Pearson correlation of plot-wise estimates of spatial trends for SwiVar21 which are shown in Fig. S27. Just correlations significant at $p < 0.001$ are shown.

S29 Correlation of plot-wise estimates of spatial field trends for individual flights - SwiVar 2022

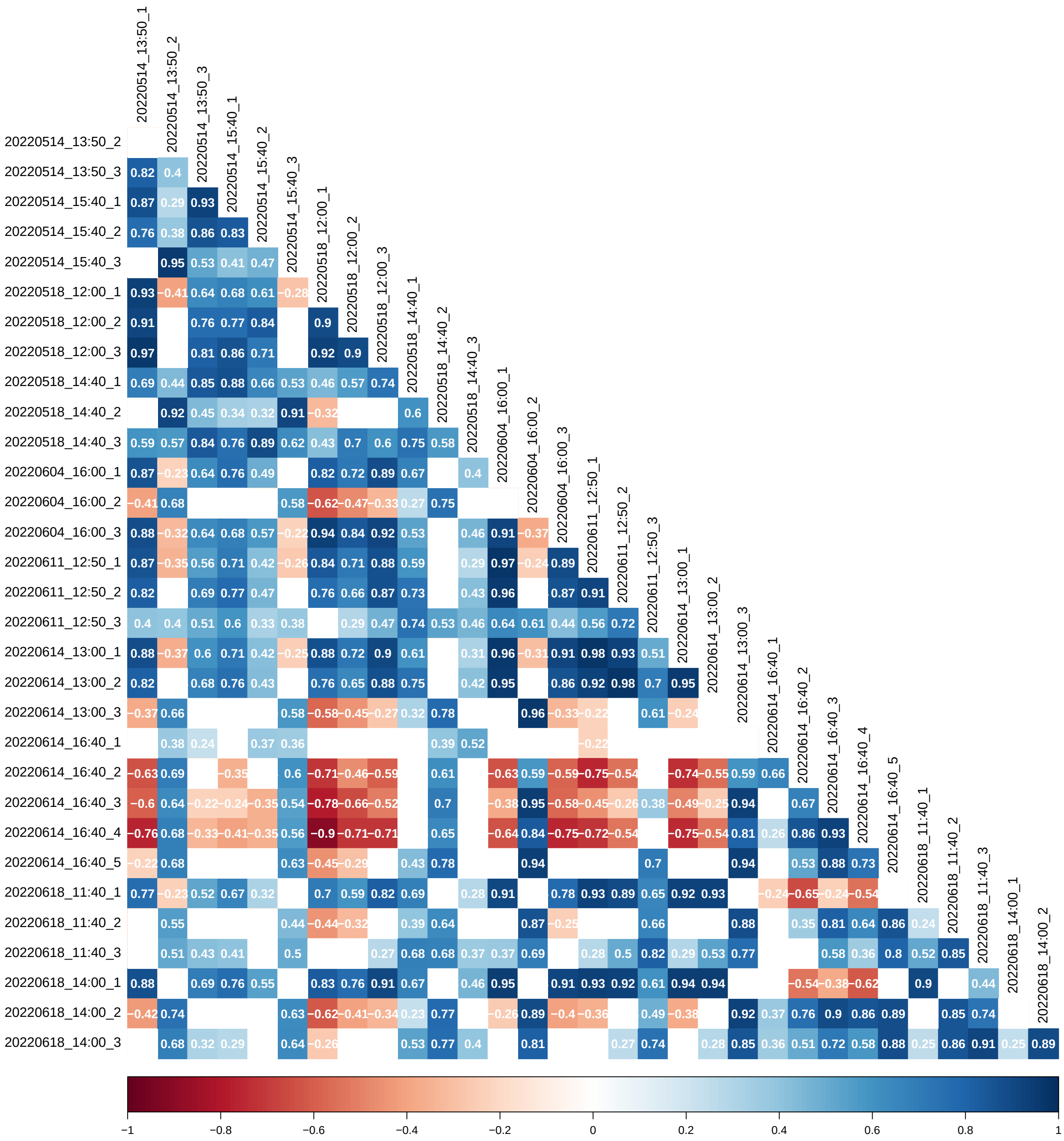

Figure S31: Pearson correlation of plot-wise estimates of spatial trends for SwiVar22 which are shown in Fig. S27. Just correlations significant at p < 0.001 are shown.

S30 CT differences from mean, arranged by flag leaf rolling ratings - EuVar22

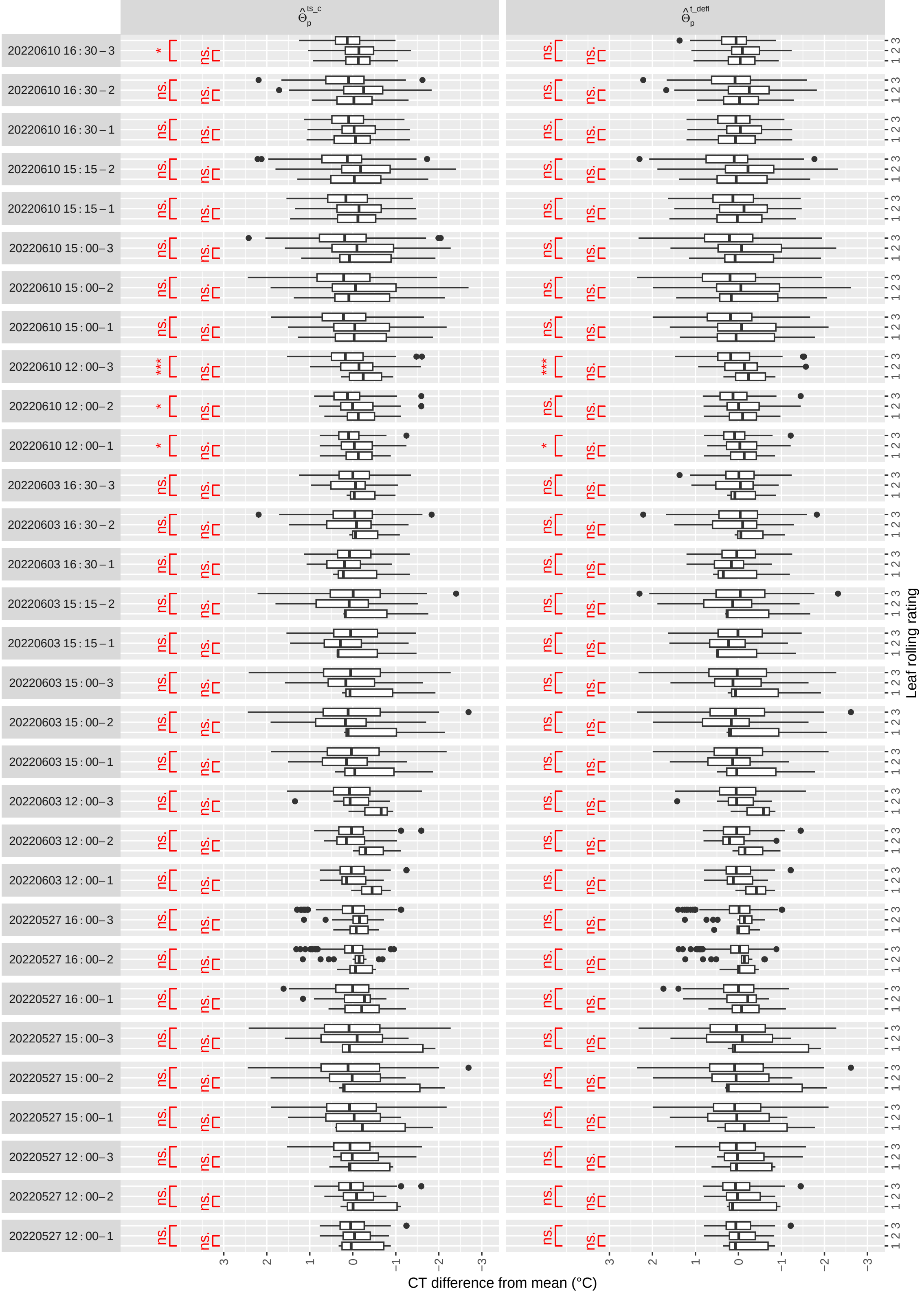

Figure S32: Corrected CT estimates of were grouped according to their flag leaf rolling score for EuVar22 before $(\hat{\theta}_p^{ts\_c})$ and after $(\hat{\theta}_p^{t\_defl})$ applying a treatment deflation on CT estimates. Significance levels of differences according to a Wilcoxon signed-rank test: ns: $p > 0.05$; *: $p < 0.05$; **: $p < 0.01$; ***: $p < 0.001$.

S31 CT differences from mean, arranged by flag leaf rolling ratings - SwiVar22 Part I

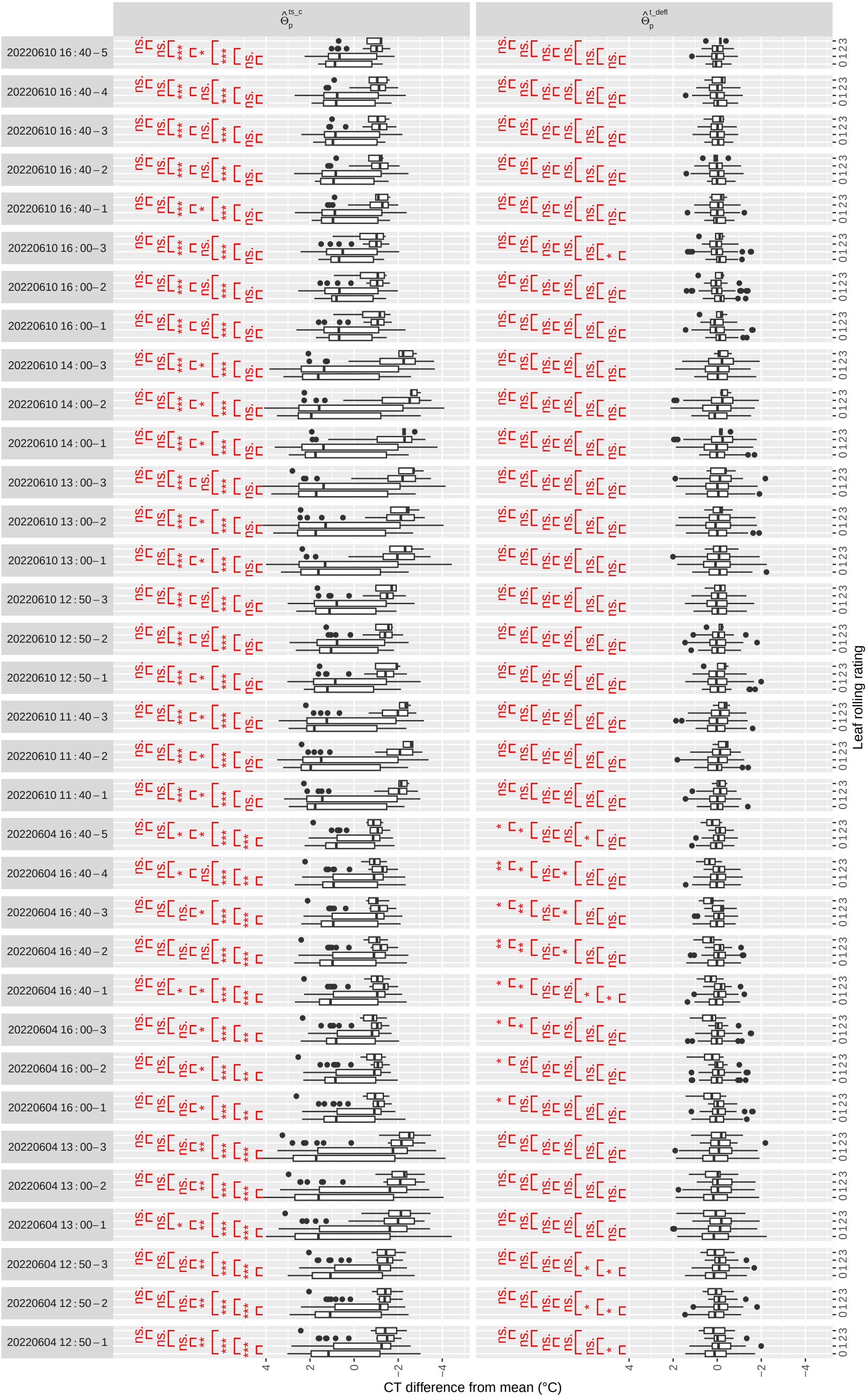

Figure S33: Corrected CT estimates were grouped according to their flag leaf rolling score for SwiVar22 (2022-06-04 & 2022-06-10) before ($\hat{\theta}_p^{ts\_c}$) and after ($\hat{\theta}_p^{t\_defl}$) applying a treatment deflation on CT estimates. Significance levels of differences according to a Wilcoxon signed-rank test: ns: $p > 0.05$; *: $p < 0.05$; **: $p < 0.01$; ***: $p < 0.001$.

S32 CT differences from mean, arranged by flag leaf rolling ratings - SwiVar22 Part II

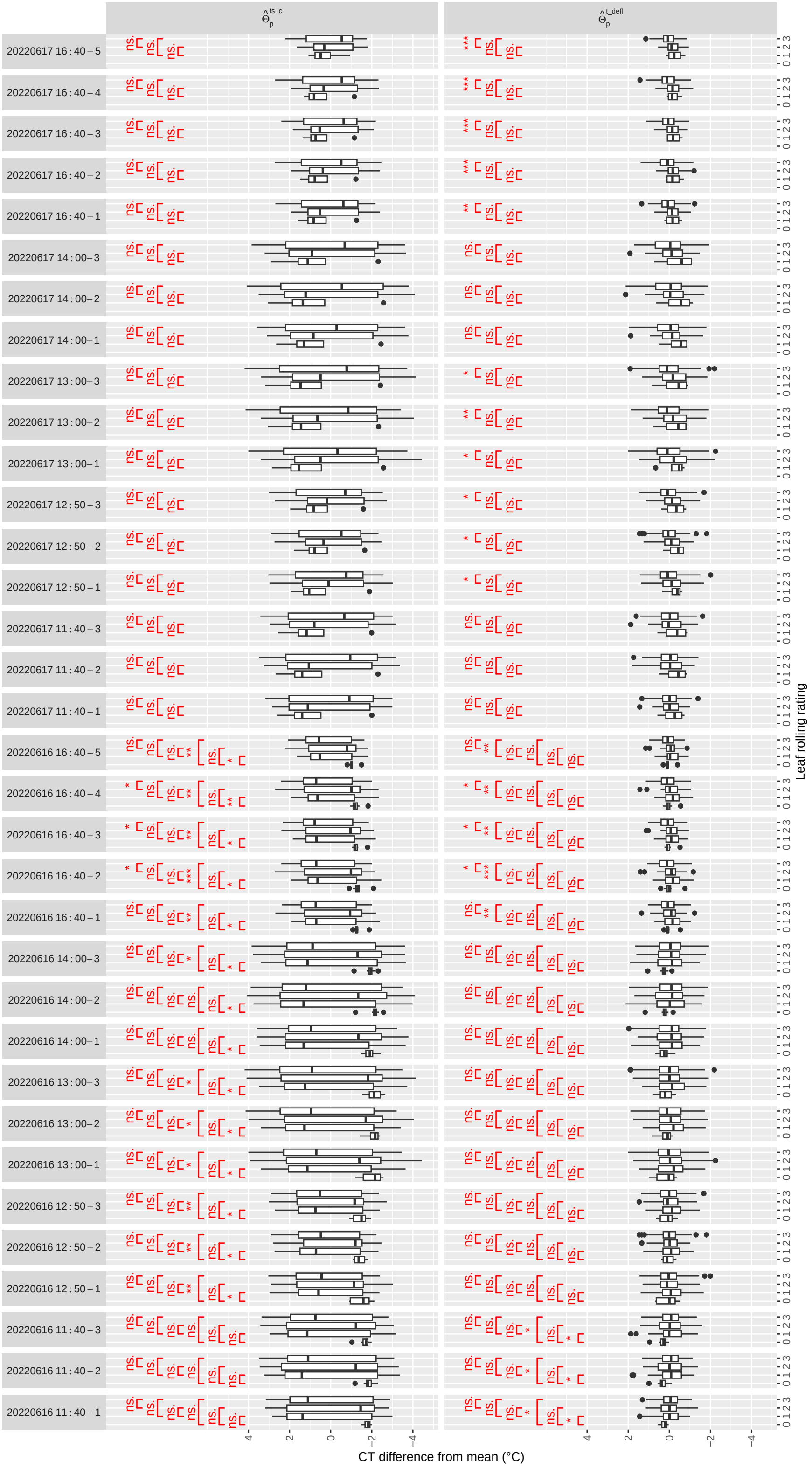

Figure S34: Corrected CT estimates were grouped according to their flag leaf rolling score for SwiVar22 (2022-06-16 & 2022-06-17) before ($\hat{\theta}_p^{ts\_c}$) and after ($\hat{\theta}_p^{t\_defl}$) applying a treatment deflation on CT estimates. Significance levels of differences according to a Wilcoxon signed-rank test: ns: $p > 0.05$; *: $p < 0.05$; **: $p < 0.01$; ***: $p < 0.001$.

S33 Flag leaf rolling ratings of SwiVar22 on 2022-06-10

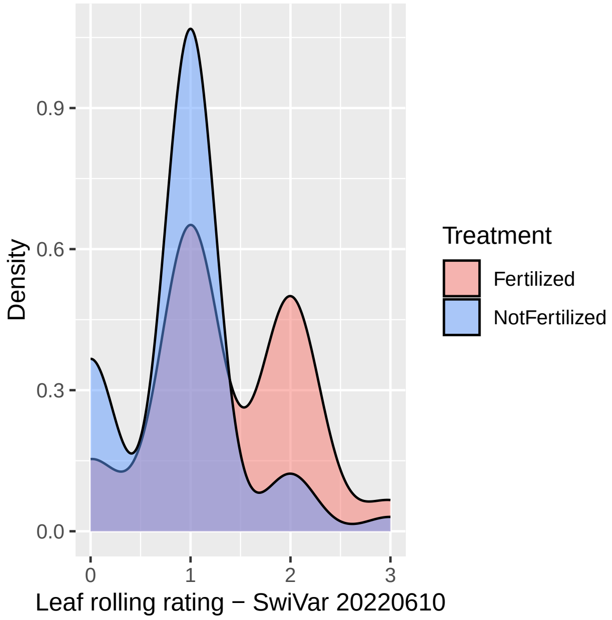

Figure S35: Flag leaf rating density distribution for SwiVar on 2022-06-10 for the two treatments "Fertilized" and "Not fertilized".

S34 Campaign-wise spatial trends EuVar21 & EuVar22

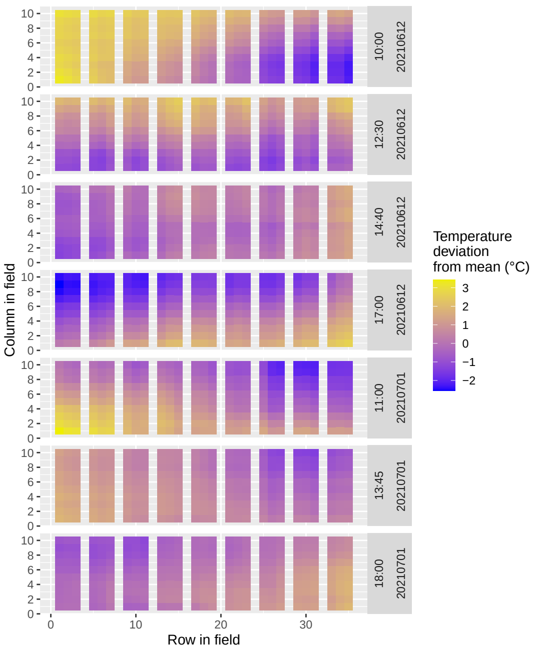

(a) .

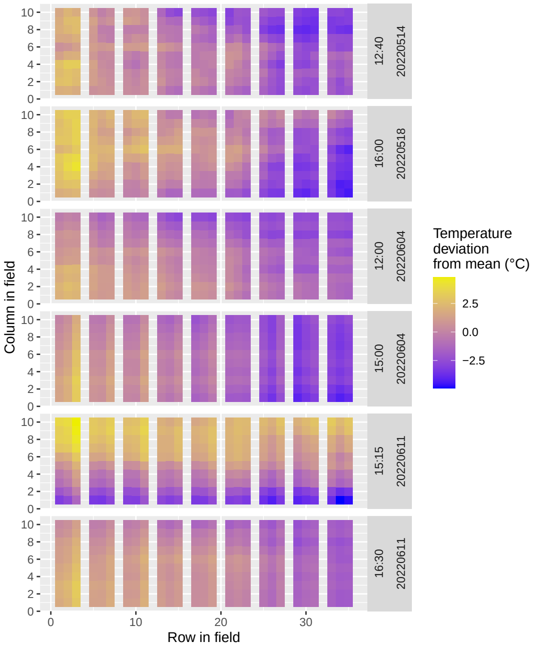

(b)

Figure S36: Spatial trend estimates for campaigns, based on Eq. 10 for EuVar21 (a) and EuVar22 (b) when processing multiple flights of a campaign with the same mixed model. Flights are horizontally grouped by dates and flight times. “Column in field” and “Row in field” indicate the spatial position of the plot in in the field where column increases along the tractor track direction. To allow for a meaningful representation of contrasting temperature ranges, flight-wise temperature deviations from flight-wise mean values are shown.

S35 Campaign-wise spatial trends SwiVar21 & SwiVar22

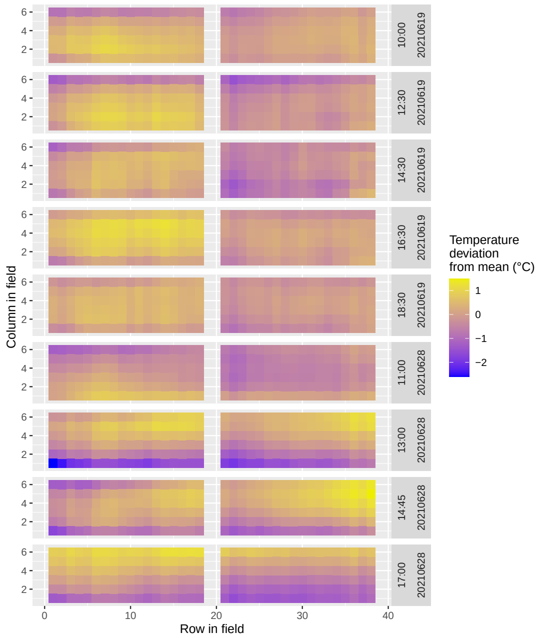

(a) .

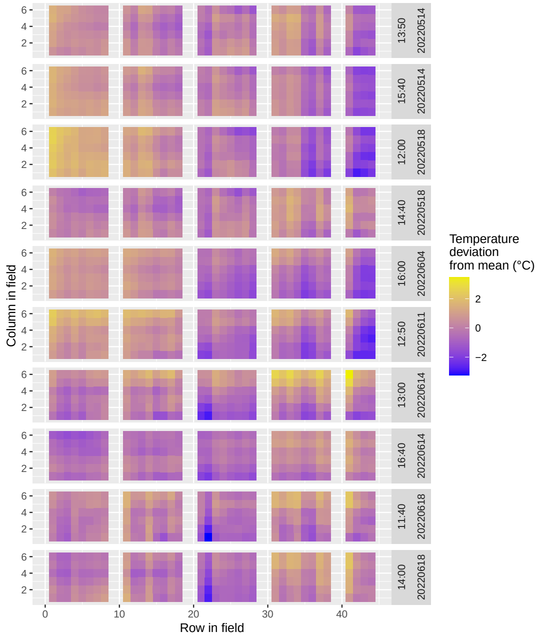

(b)

Figure S37: Spatial trend estimates for campaigns, based on Eq. 10 for SwiVar21 (a) and SwiVar22 (b) when processing multiple flights of a campaign with the same mixed model. Flights are horizontally grouped by dates and flight times. “Column in field” and “Row in field” indicate the spatial position of the plot in in the field where column increases along the tractor track direction. To allow for a meaningful representation of contrasting temperature ranges, flight-wise temperature deviations from flight-wise mean values are shown.

S36 Detailed correlation charts of campaign-wise spatial trends - EuVar21

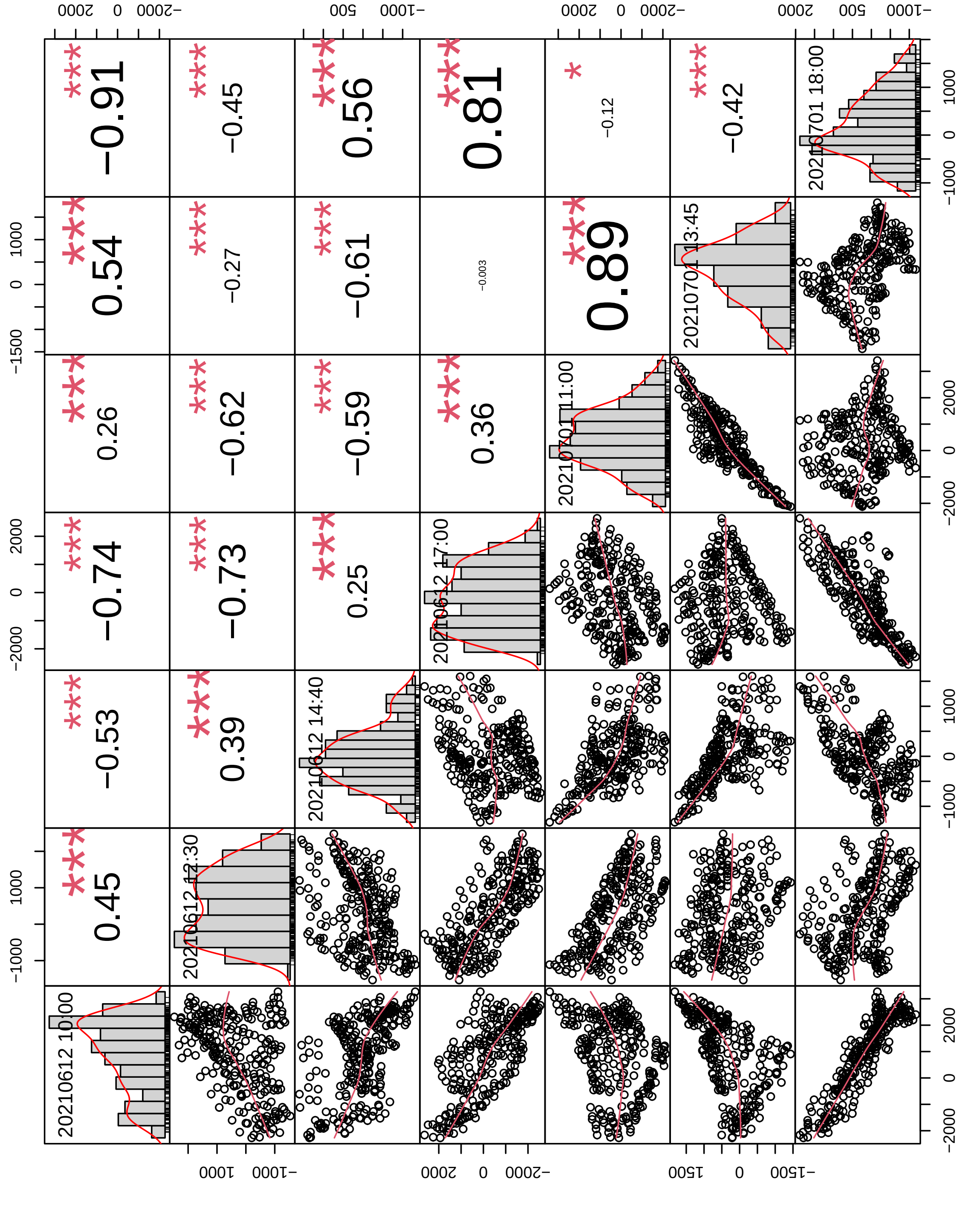

Figure S38: Pearson correlation of estimates of spatial trends according to Eq. 10 between campaigns of EuVar21 (data of Fig. S36a).

S37 Detailed correlation charts of campaign-wise spatial trends - EuVar22

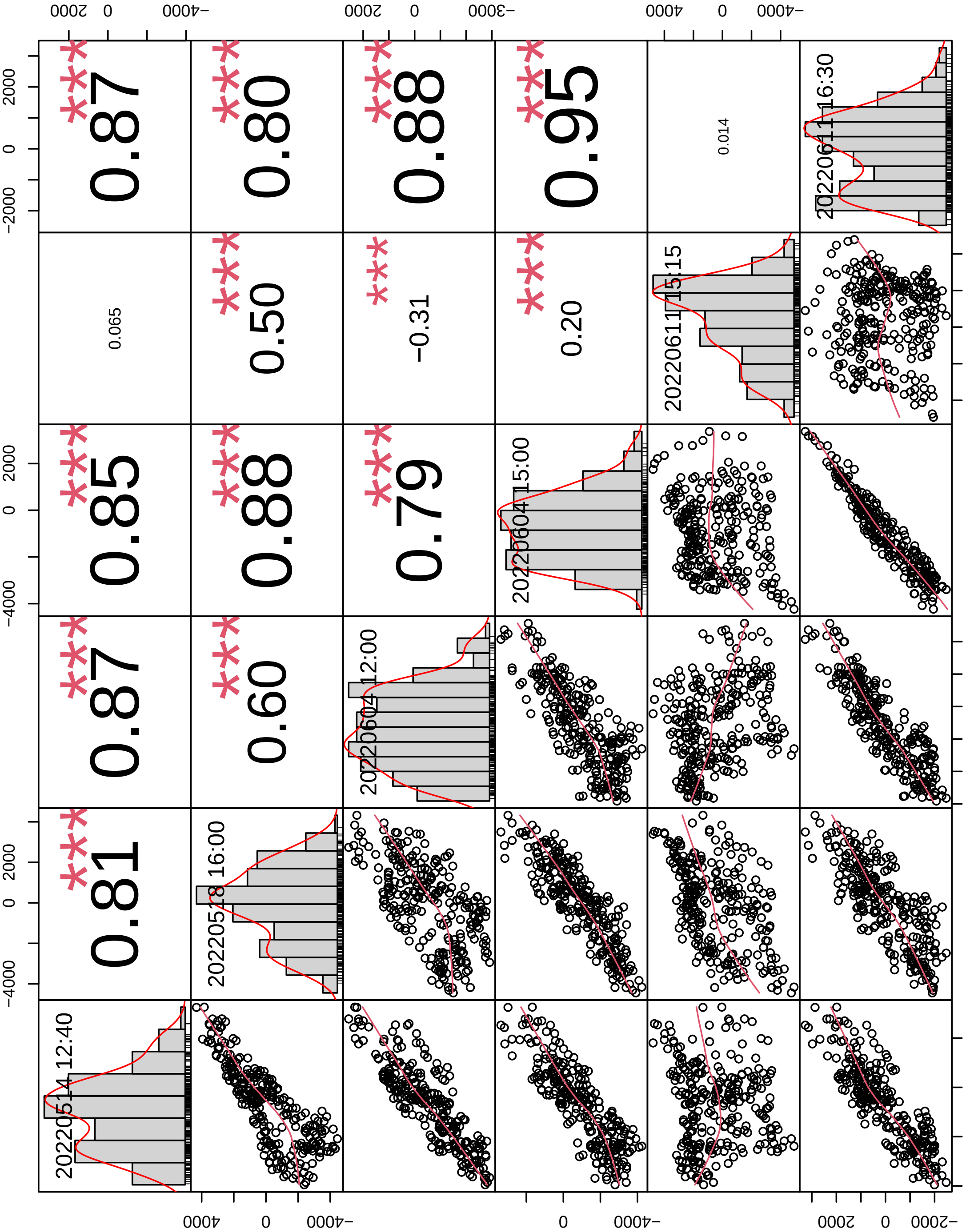

Figure S39: Pearson correlation of estimates of spatial trends according to Eq. 10 between campaigns of EuVar22 (data of Fig. S36b).

S38 Detailed correlation charts of campaign-wise spatial trends - SwiVar21

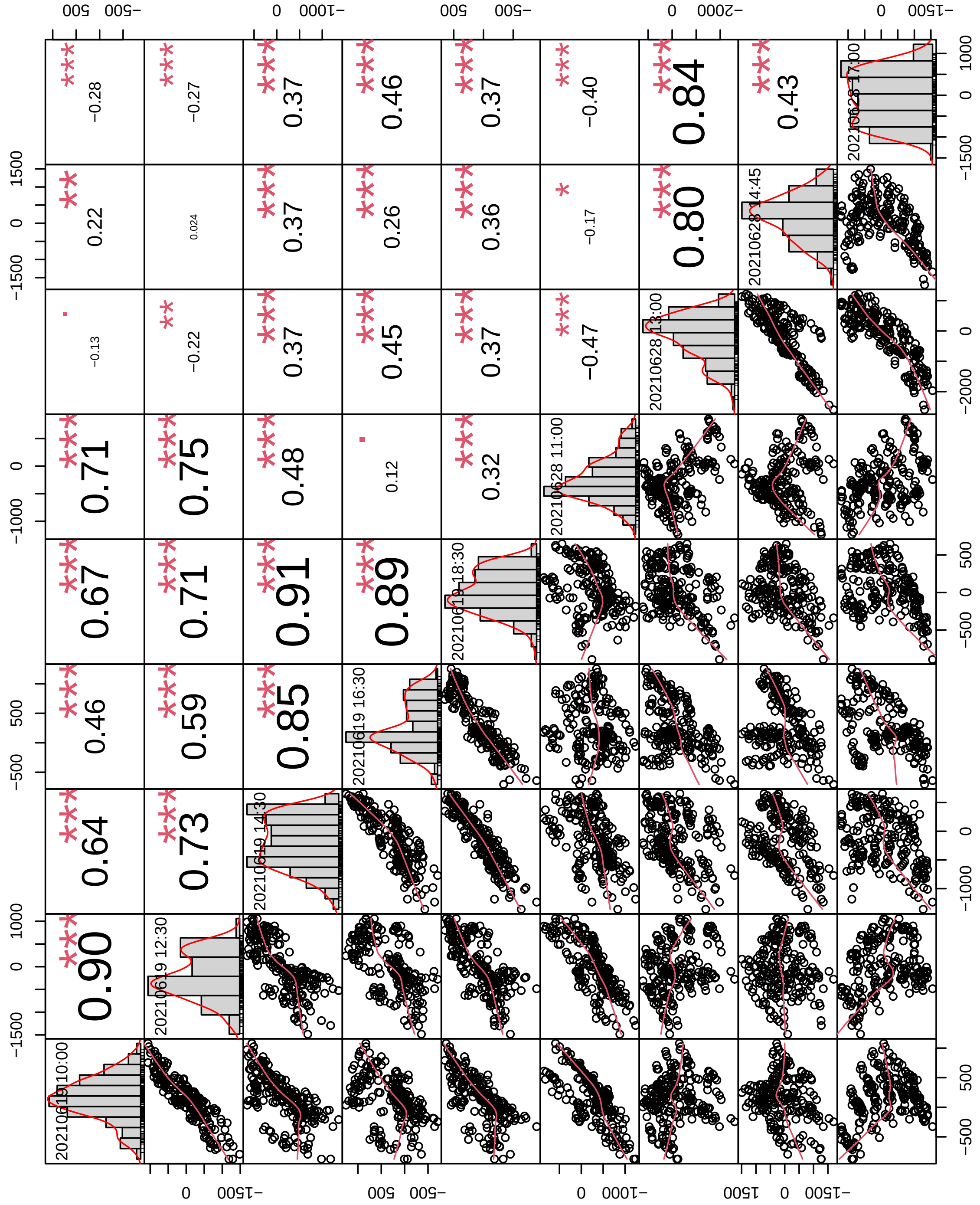

Figure S40: Pearson correlation of estimates of spatial trends according to Eq. 10 between campaigns of SwiVar21 (data of Fig. S37a).

S39 Detailed correlation charts of campaign-wise spatial trends - SwiVar22

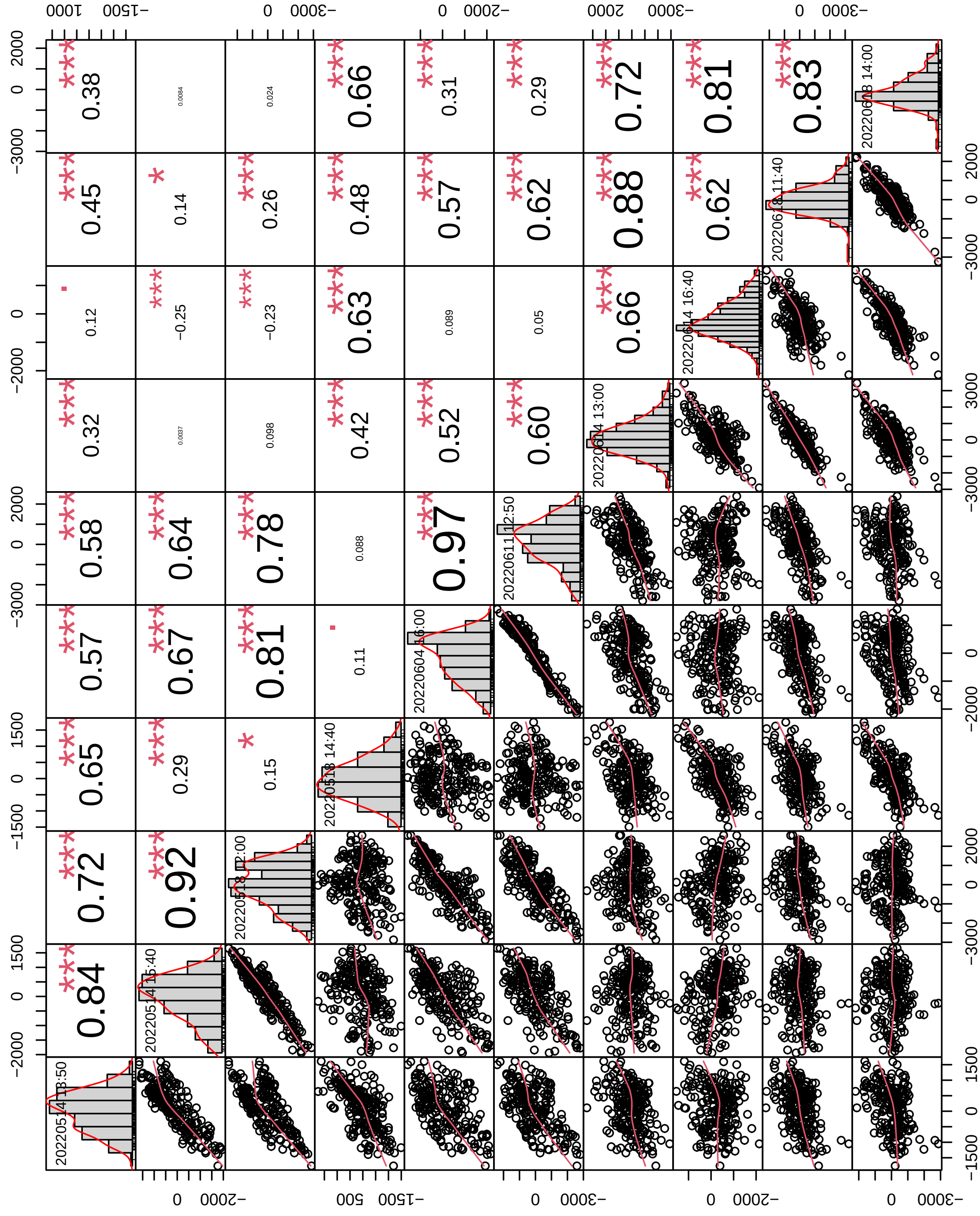

Figure S41: Pearson correlation of estimates of spatial trends according to Eq. 10 between campaigns of SwiVar22 (data of Fig. S37b).

S40 Flight-wise variance reduction by mixed models and PLSR - EuVar without vignetting correction

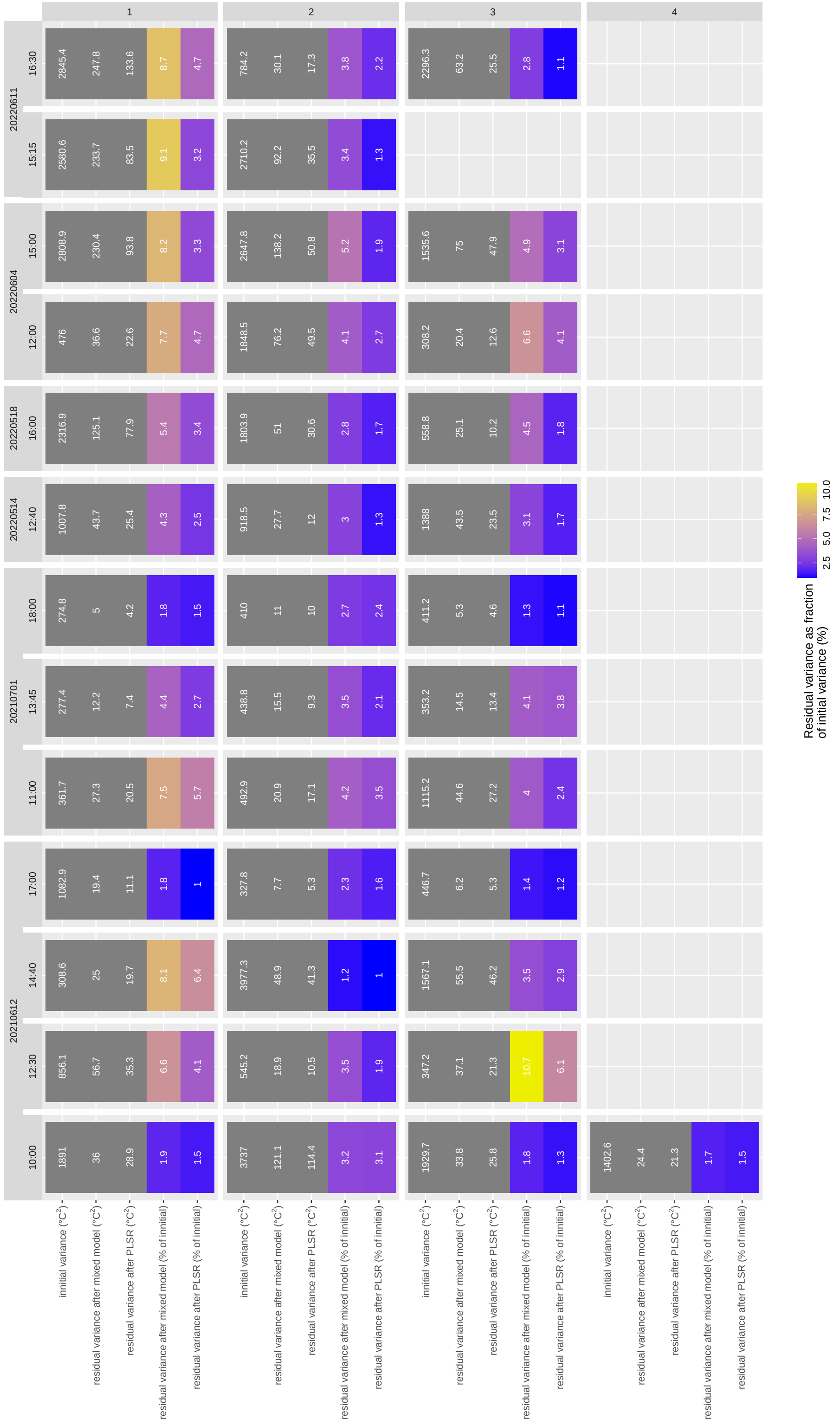

Figure S42: CT estimate variance reduction by mixed model and PLSR for EuVar without vignetting correction applied. Variance is shown for initial estimates (multiple per plot), after mixed models and after PLSR. Variance after mixed models and PLSR are also indicated as % of initial variance. Individual campaigns are arranged in columns, the rows represent the flights within the campaigns.

S41 Flight-wise variance reduction by mixed models and PLSR - EuVar with vignetting correction

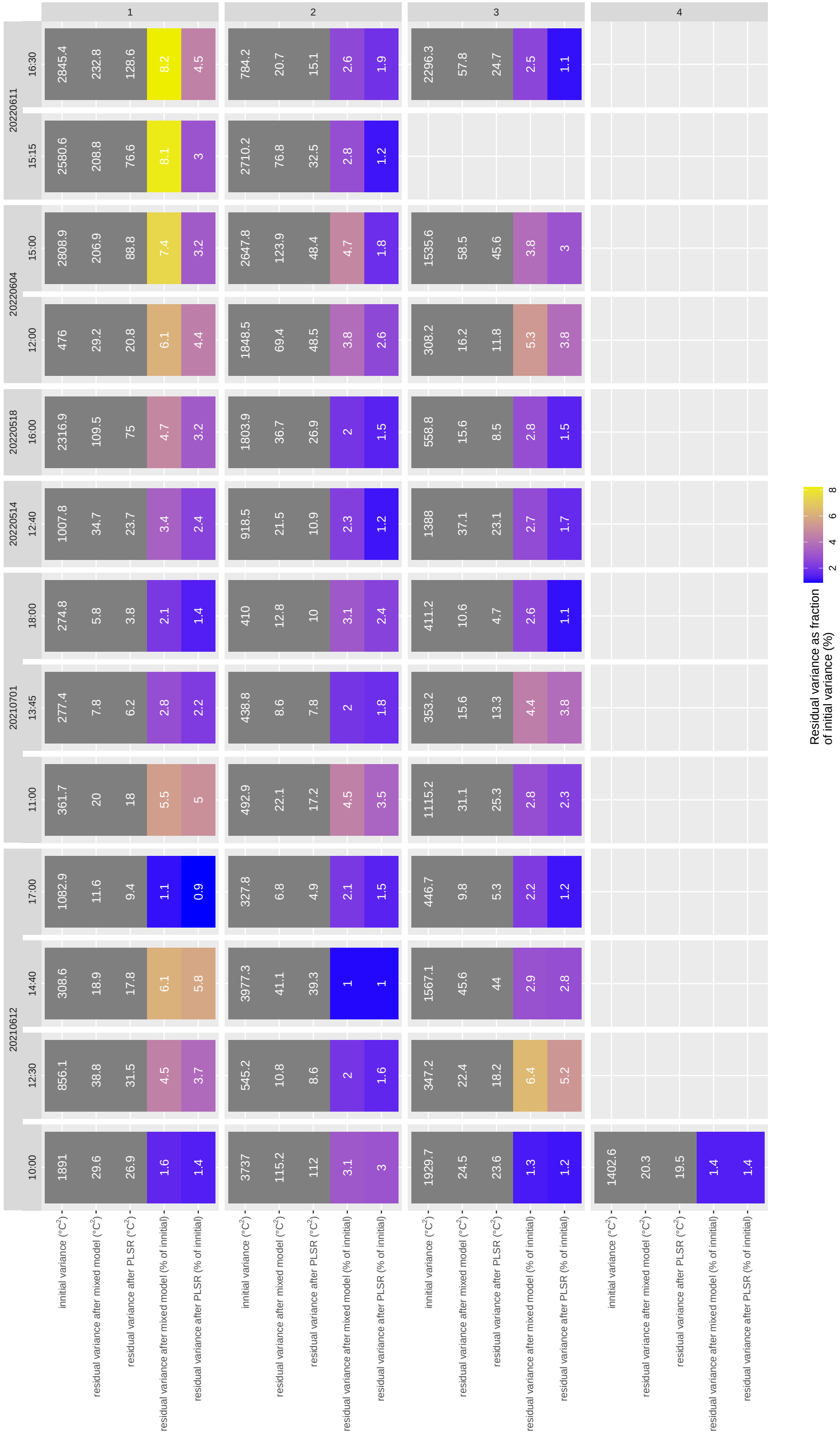

Figure S43: CT estimate variance reduction by mixed model and PLSR for EuVar with vignetting correction applied. Variance is shown for initial estimates (multiple per plot), after mixed models and after PLSR. Variance after mixed models and PLSR are also indicated as % of initial variance. Individual campaigns are arranged in columns, the rows represent the flights within the campaigns.

S42 Flight-wise variance reduction by mixed models and PLSR - SwiVar without vignetting correction

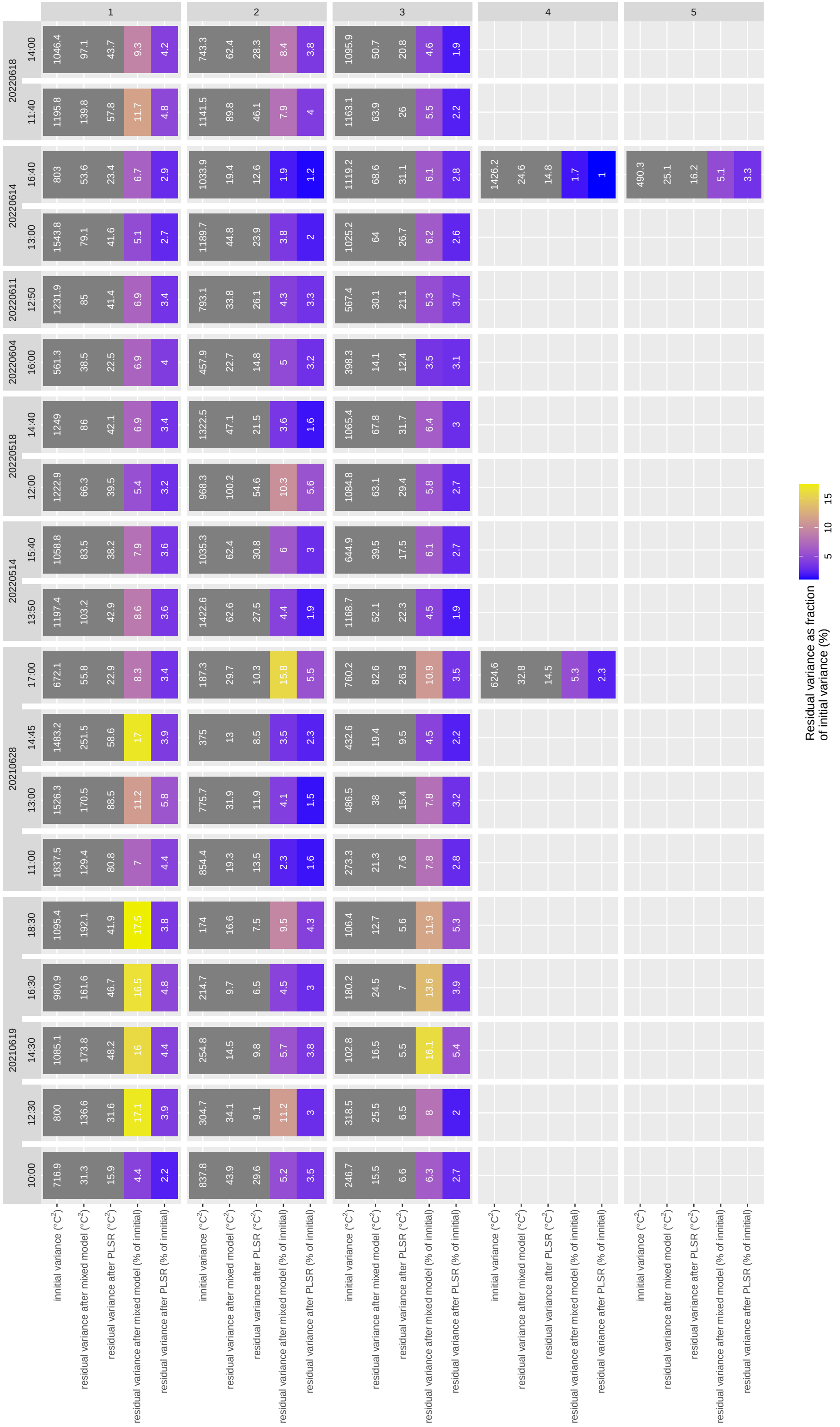

Figure S44: CT estimate variance reduction by mixed model and PLSR for SwiVar without vignetting correction applied. Variance is shown for initial estimates (multiple per plot), after mixed models and after PLSR. Variance after mixed models and PLSR are also indicated as % of initial variance. Individual campaigns are arranged in columns, the rows represent the flights within the campaigns.

S43 Flight-wise variance reduction by mixed models and PLSR - SwiVar with vignetting correction

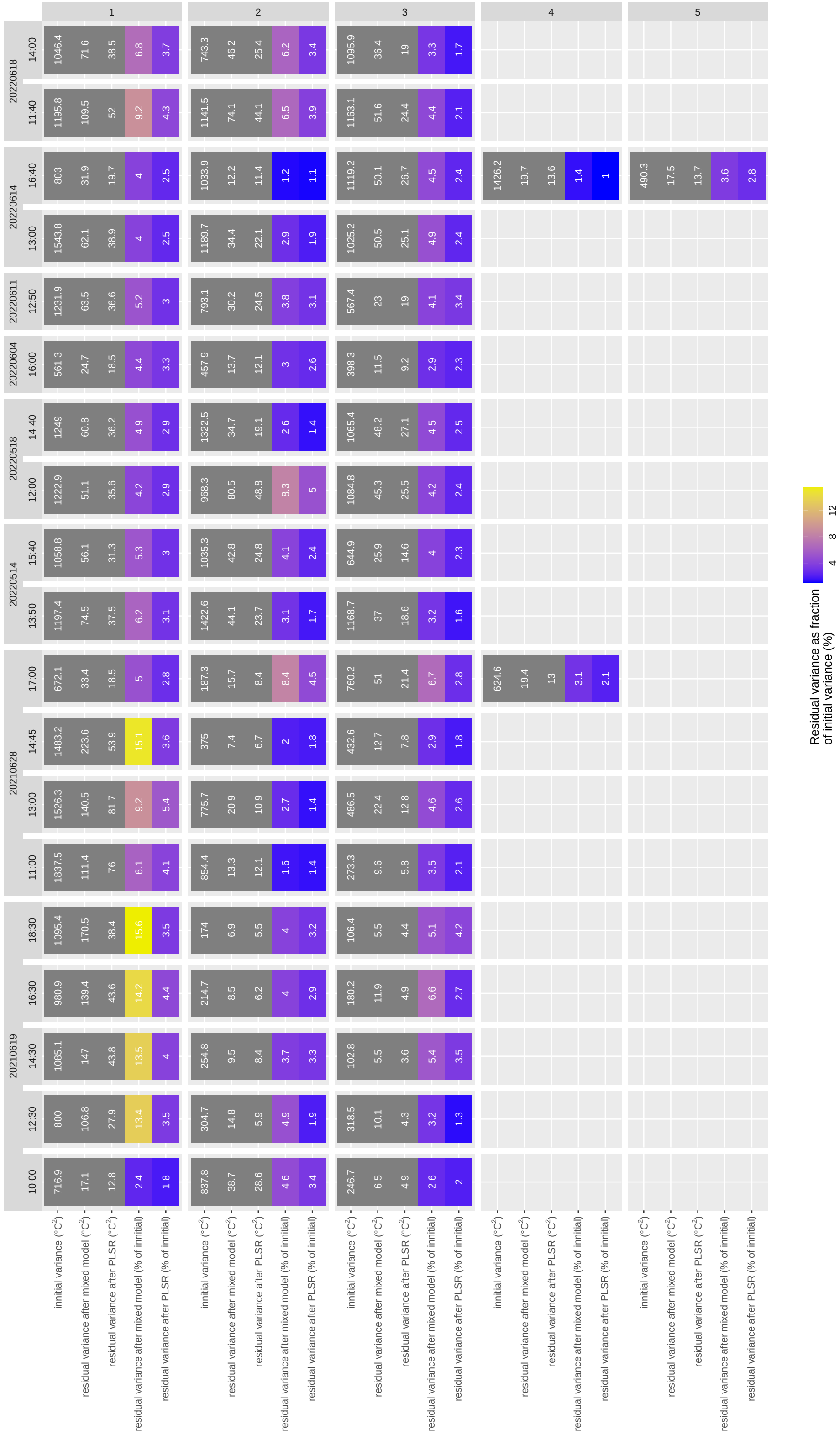

Figure S45: CT estimate variance reduction by mixed model and PLSR for SwiVar with vignetting correction applied. Variance is shown for initial estimates (multiple per plot), after mixed models and after PLSR. Variance after mixed models and PLSR are also indicated as % of initial variance. Individual campaigns are arranged in columns, the rows represent the flights within the campaigns.

Bibliography

Aragon, Bruno, Kasper Johansen, Stephen Parkes, Yoann Malbeteau, Samir Al-Mashharawi, Talal Al-Amoudi, Cristhian F. Andrade, Darren Turner, Arko Lucieer, and Matthew F. McCabe (June 2020). "A calibration procedure for field and UAV-based uncooled thermal infrared instruments". en. In: *Sensors* 20.11, p. 3316. ISSN: 1424-8220. DOI: 10.3390/s20113316. (Visited on 06/15/2023).

Benassi, Francesco, Elisa Dall'Asta, Fabrizio Diotri, Gianfranco Forlani, Umberto Morra di Cella, Riccardo Roncella, and Marina Santise (Feb. 2017). "Testing accuracy and repeatability of UAV blocks oriented with GNSS-supported aerial triangulation". en. In: *Remote Sensing* 9.2, p. 172. ISSN: 2072-4292. DOI: 10.3390/rs9020172. (Visited on 04/28/2022).

Boesch, R. (Aug. 2017). "Thermal remote sensing with UAV-based workflows". en. In: *The International Archives of the Photogrammetry, Remote Sensing and Spatial Information Sciences* XLII-2/W6, pp. 41–46. ISSN: 2194-9034. DOI: 10.5194/isprs-archives-XLII-2-W6-41-2017. (Visited on 05/17/2024).

Deery, David M., Greg J. Rebetzke, Jose A. Jimenez-Berni, Richard A. James, Anthony G. Condon, William D. Bovill, Paul Hutchinson, Jamie Scarrow, Robert Davy, and Robert T. Furbank (Dec. 2016). "Methodology for high-throughput field phenotyping of canopy temperature using airborne thermography". en. In: *Frontiers in Plant Science* 7. ISSN: 1664-462X. DOI: 10.3389/fpls.2016.01808. (Visited on 04/04/2022).

Kelly, Julia, Natascha Kljun, Per-Ola Olsson, Laura Mihai, Bengt Liljeblad, Per Weslien, Leif Klemedtsson, and Lars Eklundh (Mar. 2019). "Challenges and best practices for deriving temperature data from an uncalibrated UAV thermal infrared camera". en. In: *Remote Sensing* 11.5, p. 567. ISSN: 2072-4292. DOI: 10.3390/rs11050567. (Visited on 06/15/2023).

Malbéteau, Yoann, Kasper Johansen, Bruno Aragon, Samir K. Al-Mashhawari, and Matthew F. McCabe (Aug. 2021). "Overcoming the challenges of thermal infrared orthomosaics using a swath-based approach to correct for dynamic temperature and wind effects". en. In: *Remote Sensing* 13.16, p. 3255. ISSN: 2072-4292. DOI: 10.3390/rs13163255. (Visited on 03/23/2023).

Mesas-Carrascosa, Francisco-Javier, Fernando Pérez-Porras, Jose Meroño De Larriva, Carlos Mena Frau, Francisco Agüera-Vega, Fernando Carvajal-Ramírez, Patricio Martínez-Carricondo, and Alfonso García-Ferrer (Apr. 2018). "Drift correction of lightweight microbolometer thermal sensors on-board unmanned aerial vehicles". en. In: *Remote Sensing* 10.4, p. 615. ISSN: 2072-4292. DOI: 10.3390/rs10040615. (Visited on 06/16/2023).

Perich, Gregor, Andreas Hund, Jonas Anderegg, Lukas Roth, Martin P. Boer, Achim Walter, Frank Liebisch, and Helge Aasen (2020). "Assessment of multi-image unmanned aerial vehicle based high-throughput field phenotyping of canopy temperature". In: *Frontiers in Plant Science* 11.February, pp. 1–17. ISSN: 1664462X. DOI: 10.3389/fpls.2020.00150.

QGIS Development Team (2022). *QGIS geographic information system.* URL: https://www.qgis.org.

Rodríguez-Álvarez, María Xosé, Martin P Boer, Fred A van Eeuwijk, and Paul H C Eilers (2018). "Correcting for spatial heterogeneity in plant breeding experiments with P-splines". In: *Spatial Statistics* 23, pp. 52–71. DOI: 10.1016/j.spasta.2017.10.003.

Roth, Lukas, Helge Aasen, Achim Walter, and Frank Liebisch (July 2018). "Extracting leaf area index using viewing geometry effects—A new perspective on high-resolution unmanned aerial system photography". en. In: *ISPRS Journal of Photogrammetry and Remote Sensing* 141, pp. 161–175. ISSN: 09242716. DOI: 10.1016/j.isprsjprs.2018.04.012. (Visited on 04/17/2023).

Roth, Lukas, Moritz Camenzind, Helge Aasen, Lukas Kronenberg, Christoph Barendregt, Karl-Heinz Camp, Achim Walter, Norbert Kirchgessner, and Andreas Hund (Jan. 2020). "Repeated multiview imaging for estimating seedling tiller counts of wheat genotypes using drones". en. In: *Plant Phenomics* 2020, pp. 2020/3729715. ISSN: 2643-6515. DOI: 10.34133/2020/3729715. (Visited on 04/17/2023).

Treier, Simon, Juan M. Herrera, Andreas Hund, Norbert Kirchgessner, Helge Aasen, Achim Walter, and Lukas Roth (Dec. 2024). "Improving drone-based uncalibrated estimates of wheat canopy temperature in plot experiments by accounting for confounding factors in a multi-view analysis". en. In: *ISPRS Journal of Photogrammetry and Remote Sensing* 218, pp. 721–741. ISSN: 09242716. DOI: 10.1016/j.isprsjprs.2024.09.015. (Visited on 10/13/2024).

van Rossum, Guido and Drake, Fred L. (2009). *Python 3 Reference Manual.* Place: Scotts Valley, CA.

Zheng, Xiaopo, Zhao-Liang Li, Xia Zhang, and Guofei Shang (Dec. 2019). "Quantification of the adjacency effect on measurements in the thermal infrared region". en. In: *IEEE Transactions on Geoscience and Remote Sensing* 57.12, pp. 9674–9687. ISSN: 0196-2892, 1558-0644. DOI: 10.1109/TGRS.2019.2928525. (Visited on 06/20/2023).
